# Design, synthesis, and anticancer evaluation of Biginelli-derived DHPM–amide hybrids bearing clinically inspired kinase-directed pharmacophores: EGFR docking and DFT studies

**DOI:** 10.1039/d6ra08838d

**Published:** 2026-10-19

**Authors:** Sagar P. Kothadiya, Jay B. Maheta, Darshna K. Lakhnotra, Yogesh O. Bhola, Sureshkumar B. Koradiya

**Affiliations:** a Department of Chemistry, Atamiya University Rajkot 360005 Gujarat India Suresh.koradiya@gmail.com; b Department of Chemistry, Shri M. P. Pandya Science College, Shri Govind Guru University Lunawada-389230 Godhra Gujarat India dryobhola@gmail.com

## Abstract

A series of twelve novel dihydropyrimidine (DHPM)–amide hybrid conjugates (6a–c, 8a–c, 10a–c, and 12a–c) were rationally designed, synthesized, and biologically evaluated as potential EGFR-targeted anticancer agents against A549 non-small cell lung carcinoma (NSCLC) cells. The target compounds were constructed *via* a convergent two-step protocol: Biginelli multicomponent condensation to assemble the DHPM–carboxylic acid core (4a–c), followed by EDC·HCl/HOBt-mediated chemoselective amide coupling with four clinically inspired amine fragments derived from erlotinib/gefitinib (series 10), cabozantinib (series 8), imatinib (series 12), and a sorafenib bioisostere (series 6). All compounds were characterized by IR, ^1^H NMR, ^13^C NMR, and low-resolution ESI-MS. MTT cytotoxicity screening identified compound 10b as the most potent analogue (IC_50_ = 16.11 ± 0.11 µg mL^−1^, equivalent to 23.31 µM), exceeding the molar potency of the reference methotrexate (IC_50_ = 15.00 ± 0.48 µg mL^−1^, equivalent to 33.01 µM). It should be noted that methotrexate is a dihydrofolate reductase inhibitor included solely as a general cytotoxicity control and is not a mechanistically matched EGFR comparator; the comparison therefore reflects relative cytotoxic potency only and does not imply EGFR selectivity. Molecular docking against EGFR (PDB 1M17), an erlotinib-bound inactive-conformation structure, returned the most favourable score for 10b (score = −7.819 kcal mol^−1^), with predicted hydrogen bonds to ASP831 and LYS721, a halogen contact at ILE765, and an extensive 15-residue hydrophobic network; these poses indicate compatibility with the erlotinib-binding pocket rather than a confirmed ATP-competitive binding mode. DFT calculations at B3LYP/6-311G(d,p) characterised the electronic structure of the isolated molecule (Δ*E* = 4.045 eV; *ω* = 3.155 eV) and are reported as a physicochemical description only, without implying a causal link to cellular activity. Normal mode analysis indicated conformational stability of the 10b–EGFR complex, collectively identifying 10b as a promising lead for further optimisation and mechanistic evaluation, for which direct EGFR kinase assays remain necessary.

## Abstract 2

Biginelli-derived DHPM–amide hybrids were synthesized and evaluated for anticancer activity. Compound 10b showed the highest A549 cytotoxicity and favourable EGFR-binding interactions, supported by docking and DFT studies.

## Graphical abstract

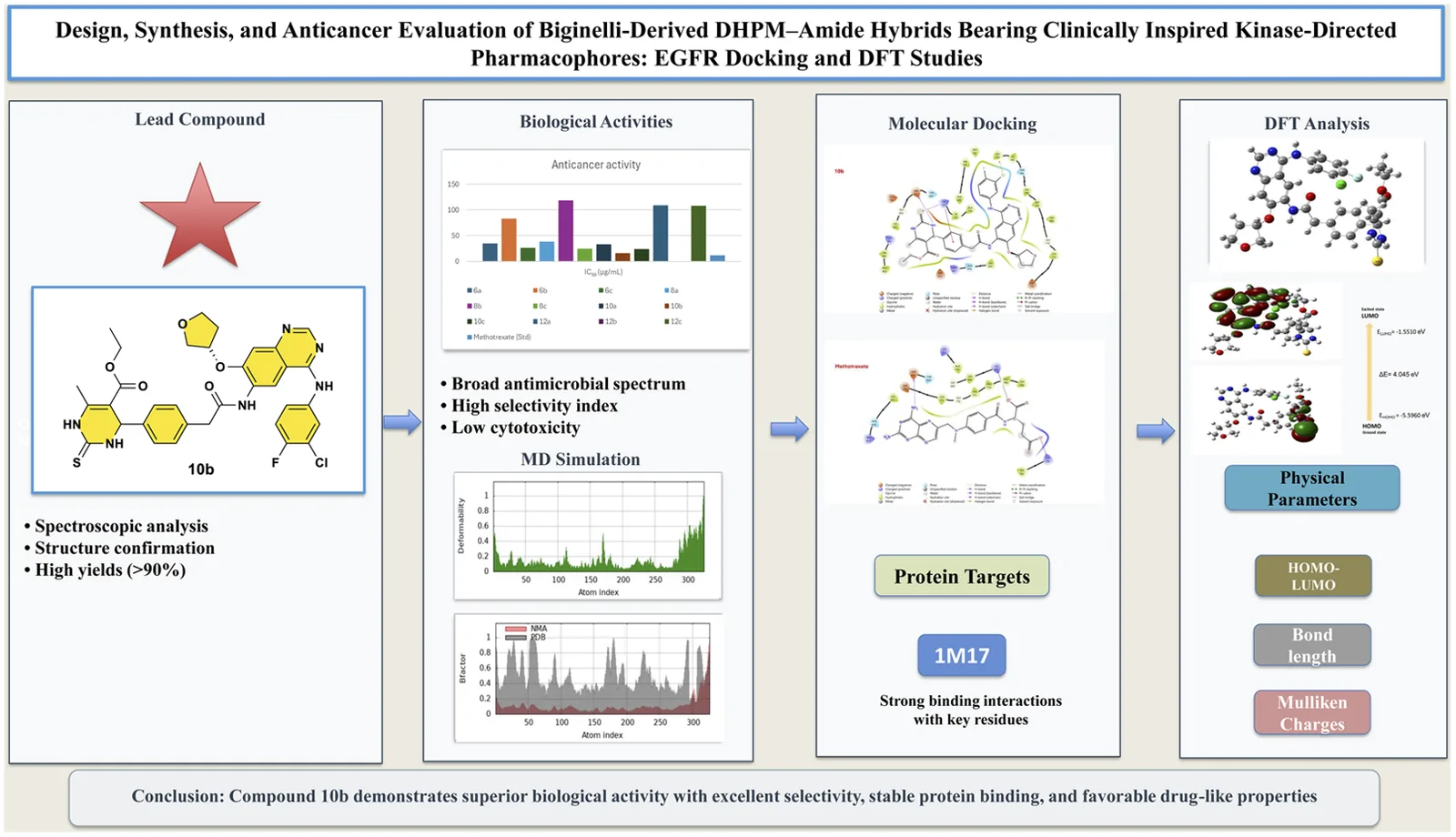

## Introduction

Lung cancer remains the leading cause of cancer mortality worldwide, responsible for an estimated 1.8 million deaths annually (GLOBOCAN 2022). Non-small cell lung cancer (NSCLC) accounts for approximately 85% of cases, with limited treatment options in advanced or drug-resistant settings.^1–3^ The A549 cell line, derived from human pulmonary adenocarcinoma, is a well-validated *in vitro* model for NSCLC research, owing to its constitutively active KRAS/MAPK signalling and clinically relevant resistance characteristics. The discovery of novel small molecules with potent, selective activity against A549 cells remains a priority in cancer medicinal chemistry.^4–6^

The amine fragments incorporated in this study were selected based on their structural relationship to approved anticancer drugs (Fig. 1). Erlotinib (Tarceva®) and gefitinib (Iressa®), first-generation EGFR-TKIs for NSCLC, provided the 4-anilinoquinazoline pharmacophore for series 10a–c. Cabozantinib (Cabometyx®), a multi-kinase inhibitor (VEGFR-2/MET/AXL), inspired the 4-(aminophenoxy)quinoline-carboxamide moiety in series 8a–c. Imatinib (Gleevec®), whose pyrimidine-aniline scaffold occupies the ABL kinase gatekeeper pocket, was the basis for the 2-aminopyrimidine fragment in series 12a–c. The trifluoromethylimidazole unit in series 6a–c represents a bioisosteric trifluoromethyl pharmacophore, as found in agents such as sorafenib.^7^ Covalent linkage of these fragments to the DHPM–TSC hub *via* a metabolically stable amide bond was expected to confer dual kinase-inhibitory and RNR-suppressive activity, as depicted conceptually in Scheme 1.^8–11^

**Fig. 1:**
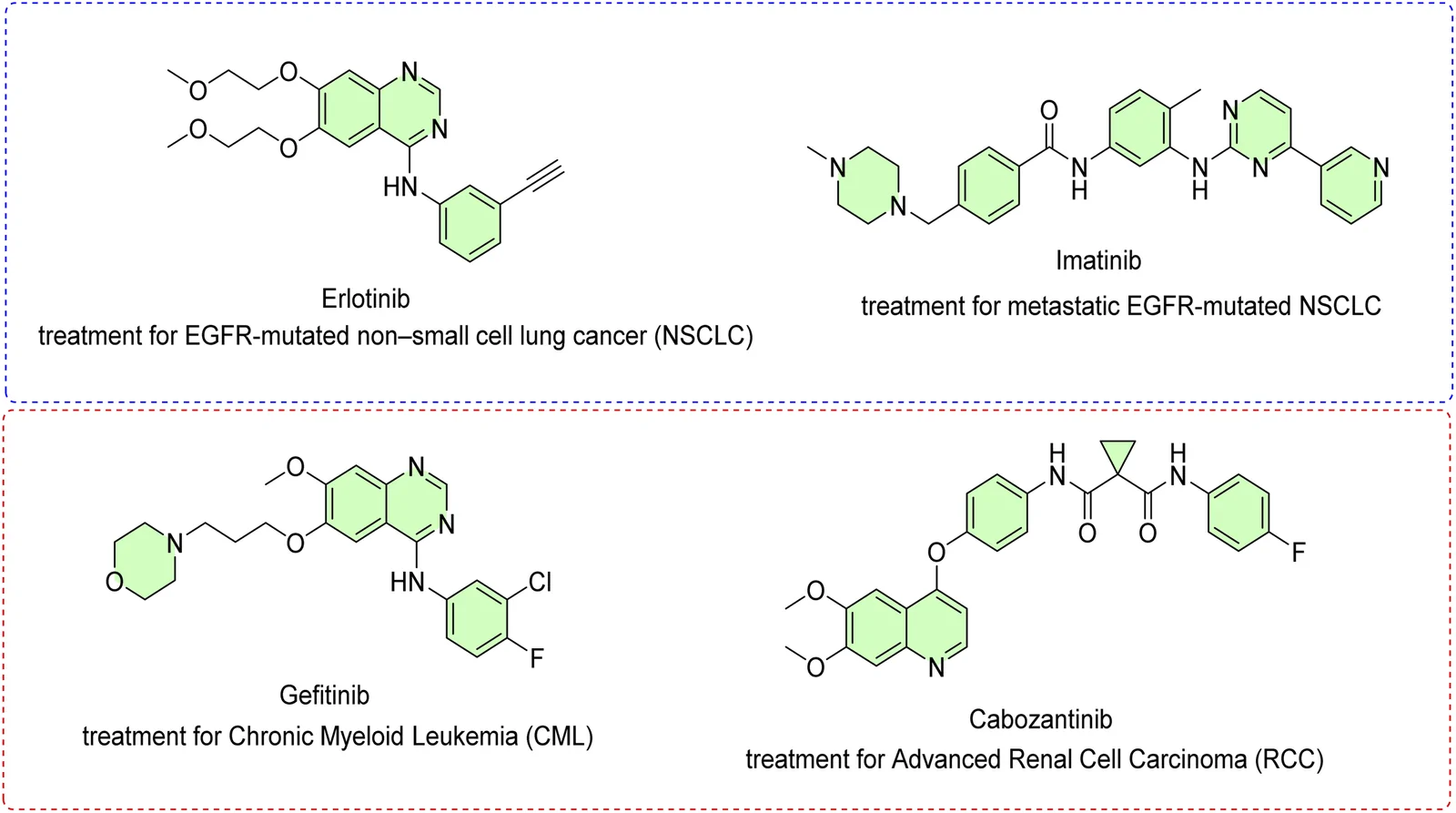
Structures of clinically approved anticancer drugs bearing a pyrimidine nucleus employed as pharmacophoric templates for the design of DHPM–amide hybrid conjugates in the present study.

**Scheme 1:**
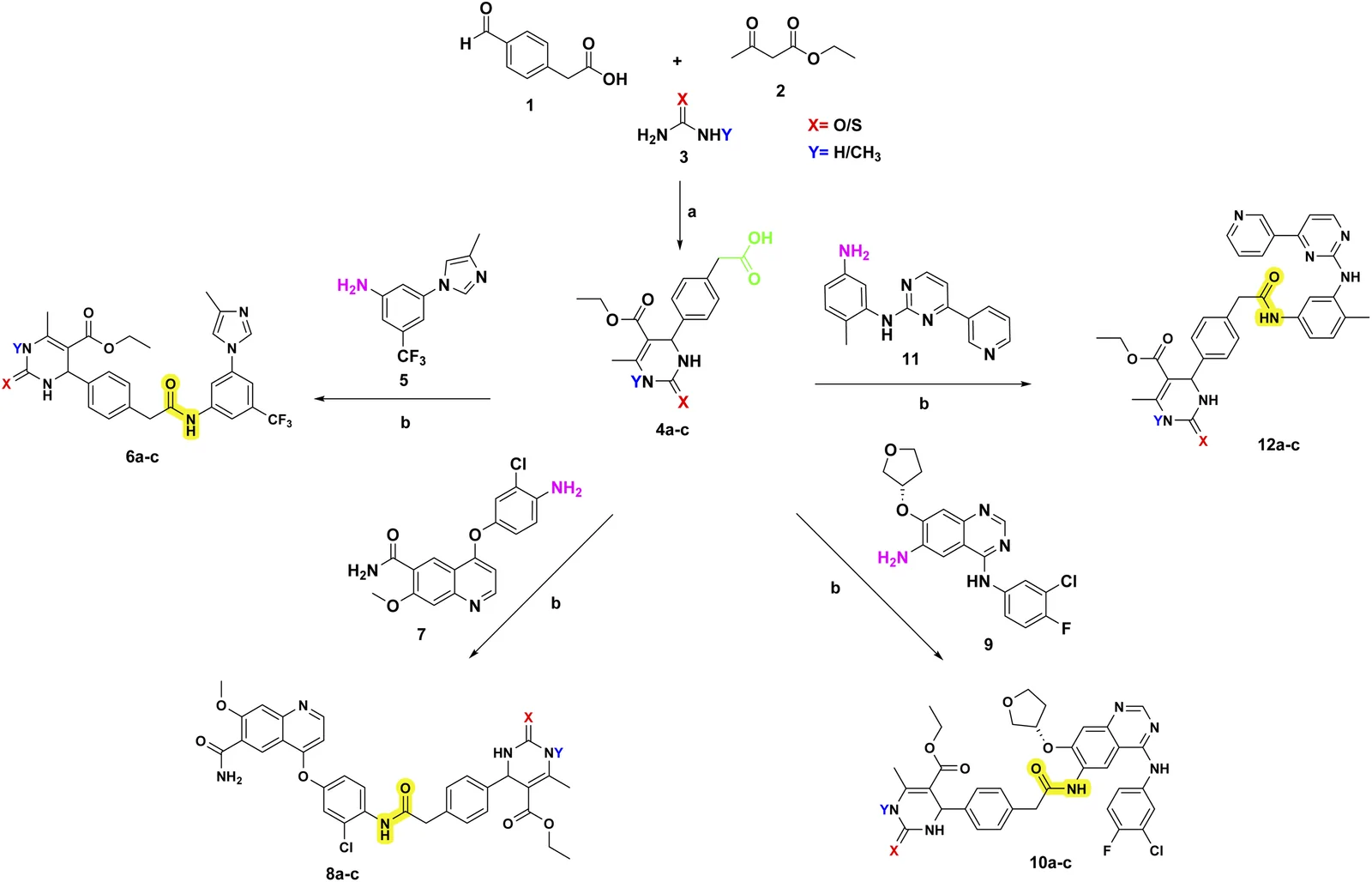
Synthetic route for the preparation of novel DHPM–amide hybrid conjugates 6a–c, 8a–c, 10a–c, and 12a–c. Reagent & conditions: (a) acetic acid, reflux, 4 h (b) EDC·HCl, HOBt, TEA, MDC.

Pharmacophore hybridisation, covalently linking two or more biologically active moieties into a single molecular entity, represents a well-established strategy to achieve multi-target inhibition, enhanced potency, and delayed emergence of resistance. In the present work, three pharmacophoric elements were combined: (i) a dihydropyrimidine (DHPM) core, accessible *via* the Biginelli multicomponent reaction, which engages kinase ATP-binding pockets and exhibits inherent antiproliferative activity; (ii) a semicarbazone (X = O) or thiosemicarbazone (X = S) arm, a well-documented metal-chelating motif that inhibits ribonucleotide reductase (RNR), an enzyme critical for DNA synthesis in rapidly dividing tumour cells; and (iii) a clinically inspired amine fragment that provides affinity for validated oncology targets including EGFR and MET kinases. The variable substituents X (=O/S) and Y (=H/CH_3_) enable systematic structure–activity relationship (SAR) interrogation within each sub-series.^12–15^

On the novelty of the hybridization strategy. It is acknowledged that combining known pharmacophoric fragments *via* an amide linker is, taken alone, a well-precedented medicinal chemistry tactic. The novelty emphasised here is not the linking chemistry itself but the specific, systematic combination realised in this study: a single DHPM–acetic acid hub concurrently diversified (a) at the C2 position (oxo/thioxo) and N1 position (H/CH_3_) and (b) through amide coupling to four mechanistically distinct, clinically inspired fragments (quinazoline/EGFR-TKI-like, quinoline/multi-kinase-like, aminopyrimidine/ABL-like, and trifluoromethylimidazole), giving a 2 × 2 × 4 matrix of twelve analogs from a common intermediate. This design allows the C2/N1 and fragment-identity contributions to potency to be deconvoluted within a single study, which is regarded as the chemical advance of the present work rather than the amide-coupling step *per se*. The Introduction and Conclusion have been revised accordingly to state this more precisely and to avoid overstating the novelty of the coupling chemistry itself.

## Result & discussion

### Chemistry

The synthetic strategy for the target compounds (6a–c, 8a–c, 10a–c, and 12a–c) is outlined in Scheme 1. The core scaffold was constructed *via* a Biginelli multicomponent condensation of 4-formylphenylacetic acid (1), ethyl acetoacetate (2), and urea/thiourea derivatives (3, X = O or S; Y = H or CH_3_) in glacial acetic acid under reflux for 4 hours, furnishing the pivotal intermediate 4 a 3,4-dihydropyrimidine-2(1*H*)-one/thione (DHPM) bearing a pendant carboxylic acid handle. The reaction proceeded *via* the well-established Knoevenagel–Michael-cyclization cascade under Brønsted acid catalysis. Successful formation of 4 was evidenced by the disappearance of the aldehyde signal and appearance of a characteristic broad N–H stretch at 3200–3350 cm^−1^ in the IR spectrum, while ^1^H NMR confirmed the diagnostic DHPM methine proton as a singlet near *δ* 5.10–5.30 ppm. The X/Y substituent variations in 3 directly installed three sub-series ((a) X = O, Y = H; (b) X = S, Y = H; (c) X = O, Y = CH_3_) consistently across all final compound series.

In the pivotal second stage, the free carboxylic acid of 4 was coupled with four structurally distinct aromatic amines 5 (3-amino-5-(trifluoromethyl)-1*H*-pyrazole), 7 (6-amino-7-methoxyquinoline-carboxamide), 9 (tetrahydrofuranyl-oxyquinazoline-aniline), and 11 (aminomethylpyridyl-pyrimidine) using EDC·HCl/HOBt/TEA in DCM at room temperature. This chemoselective amide coupling preserved the intact DHPM core while efficiently forming the amide bond. Formation of the target amide-linked hybrids was unambiguously confirmed by the emergence of a strong amide carbonyl absorption at 1650–1680 cm^−1^ alongside complete attenuation of the broad carboxylic O–H stretch (2500–3300 cm^−1^) in the IR spectra. In ^1^H NMR, a characteristic downfield NH singlet appearing at *δ* 9.2–10.1 ppm, consistent with an aromatic amide proton, corroborated successful bond formation, while ^13^C NMR revealed a new amide carbonyl signal at *δ* 165–168 ppm distinct from the DHPM carbonyl at *δ* 150–153 ppm. The amide linkage highlighted in yellow in Scheme 1 serves as a critical pharmacophoric bridge integrating the DHPM scaffold with clinically inspired fragments encompassing quinoline, quinazoline, trifluoromethylpyrazole, and aminopyrimidine motifs, collectively designed to amplify target binding through synergistic electronic and structural complementarity.

### Molecular docking results

Molecular docking of the synthesized compounds (series 6, 8, 10, and 12) was performed against the active site of EGFR tyrosine kinase (PDB ID 1M17) using Schrodinger suite. The docking scores and key binding interactions are summarized in Table 1. Methotrexate (docking score: −5.956 kcal mol^−1^) was additionally docked as an independent reference ligand for comparison; for clarity, it is noted that methotrexate is not the native co-crystallized ligand of the 1M17 structure (that ligand is erlotinib), and this terminology, which was imprecise in the original submission, has been corrected. All synthesized compounds exhibited competitive or superior docking scores relative to this methotrexate reference, with scores ranging from −5.228 kcal mol^−1^ (12b) to −7.819 kcal mol^−1^ (10b).

**Table 1:** Molecular docking results of synthesized compounds (6a–c, 8a–c, 10a–c, 12a–c) and reference inhibitors against the EGFR tyrosine kinase domain (PDB ID 1M17)

EGFR tyrosine kinase inhibitor (1M17)					
Compound code	Docking score (kcal mol^−1^)	Interacting amino acids			
		Hydrophobic interactions	Polar interactions	H-bond	Others
6a	−6.128	12 hydrophobic contacts incl. LEU834, MET742, ALA719, *et al.* (full residue list in SI Table S2)	THR830, ASN818, THR766, GLN767	MET769 (H-bond to imidazole N)	LYS721, ARG817, LYS851 (charged positive); ASP813, ASP831, GLU738 (charged negative); GLY833, GLY772 (glycine); no halogen bond
6b	−5.956	12 hydrophobic contacts incl. LEU834, GLY833, LEU764, *et al.* (full residue list in SI Table S2)	THR830, ASN818	ASP831 (H-bond backbone – salt bridge/red line to phenyl ring)	LYS721 (charged positive); ASP813, GLU738 (charged negative); ALA719
6c	−7.119	9 hydrophobic contacts incl. LEU834, GLY833, LEU768, *et al.* (full residue list in SI Table S2)	THR830, ASN818, GLN767, THR766	ASP831 (backbone); ASN818–LYS721 (H-bond sidechain, red line)	LYS721, ARG817 (charged positive); GLU738 (charged negative); MET769 (H-bond sidechain)
8a	−6.788	13 hydrophobic contacts incl. TRP856, PRO890, PRO853, *et al.* (full residue list in SI Table S2)	ASN818, THR830	ASN818 (H-bond backbone); LEU768 (H-bond sidechain); ARG779–NH_2_ (H-bond)	ARG817, ARG779, LYS889 (charged positive); ASP813, GLU882, ASP776 (charged negative); Cl halogen bond; ASN818–Cl halogen bond
8b	−7.587	14 hydrophobic contacts incl. GLY772, CYS773, LEU694, *et al.* (full residue list in SI Table S2)	ASN818, ALA698	MET769 (H-bond sidechain); ALA698–NH_2_ (H-bond)	LYS721, ARG817 (charged positive); ASP813, ASP831, GLU734 (charged negative); pi–cation: Lys721; Cl halogen
8c	−6.334	13 hydrophobic contacts incl. LEU723, GLY833, ALA719, *et al.* (full residue list in SI Table S2)	ASN818, THR830, GLN767, THR766	GLU738–NH_2_ (H-bond sidechain); LYS721 (H-bond backbone)	LYS721, ARG817 (charged positive); ASP831, GLU738, GLU734 (charged negative); Cl halogen
10a	−6.245	14 hydrophobic contacts incl. LEU834, GLY833, ALA719, *et al.* (full residue list in SI Table S2)	THR830, ASN818, THR766	ASP831 (H-bond backbone/sidechain); LYS721 (pi–cation, red line); THF ring–ASP776	LYS721, ARG817, LYS851 (charged positive); ASP813, GLU738, ASP776 (charged negative); F, Cl halogen; ILE720
10b	−7.819	15 hydrophobic contacts incl. GLY833, LEU834, ALA719, *et al.* (full residue list in SI Table S2)	THR830, ASN818	ASP831 (H-bond sidechain); LYS721 (H-bond backbone, pi-cation); THF ring–ASP776	LYS721, ARG817, LYS851 (charged positive); ASP813, GLU738, ASP776 (charged negative); F, Cl halogen; ILE765 (halogen bond)
10c	−6.843	11 hydrophobic contacts incl. MET742, LEU764, MET769, *et al.* (full residue list in SI Table S2)	ASN818, THR830	ASP831 (halogen bond to Cl); MET769 (H-bond sidechain)	ARG817 (charged positive); GLU738 (charged negative); ASP776 (charged negative); F, Cl halogen; (*S*)-THF ether
12a	−6.598	12 hydrophobic contacts incl. PHE699, ALA698, GLY697, *et al.* (full residue list in SI Table S2)	ASN818, THR766, GLN767	ARG817 (H-bond backbone); MET769 (H-bond sidechain ×2)	LYS721, ARG817, LYS704 (charged positive); ASP831, THR830 (polar); no halogen
12b	−5.228	14 hydrophobic contacts incl. PRO770, MET769, LEU768, *et al.* (full residue list in SI Table S2)	ASN818, THR830	LYS721 (pi–cation, red lines); ASP831 (backbone)	LYS721, ARG817 (charged positive); GLU738, GLU734 (charged negative); ILE735
12c	−5.507	13 hydrophobic contacts incl. LEU694, LEU768, MET769, *et al.* (full residue list in SI Table S2)	ASN818, GLN767, THR766	LYS721 (pi–cation, red line); ASP831–THR830 (backbone)	LYS721, ARG817 (charged positive); GLU734, ASP831 (charged negative); no halogen
Methotrexate	−5.956	11 hydrophobic contacts incl. GLY695, LEU694, PHE771, *et al.* (full residue list in SI Table S2)	ASN818, THR830	ASP831 (H-bond sidechain); ASP776 (H-bond backbone); LYS692 (salt bridge)	LYS721, ARG817, LYS704, LYS692 (charged positive); ASP831, ASP776 (charged negative)

Among the tested compounds, 10b emerged as the most potent binder (−7.819 kcal mol^−1^), followed by 8b (−7.587 kcal mol^−1^), 6c (−7.119 kcal mol^−1^), and 10c (−6.843 kcal mol^−1^). The superior binding affinity of compound 10b can be attributed to its extensive hydrophobic contacts, multiple hydrogen bonds, and unique halogen bonding interactions within the ATP-binding cleft of EGFR.

Compound 10b established an extensive network of hydrophobic interactions with the nonpolar lining of the active site, engaging residues GLY833, LEU834, ALA719, ILE720, MET742, LEU764, ILE765, LEU768, MET769, GLY772, CYS773, LEU694, PHE699, VAL702, and LEU820. These contacts stabilize the core scaffold of 10b within the hydrophobic spine of the kinase. Polar contacts were formed with THR830 and ASN818. Hydrogen bonds were predicted to ASP831 (sidechain H-bond) and LYS721 (backbone H-bond and pi–cation interaction), while the (*S*)-configured tetrahydrofuran-3-yl ether oxygen engaged ASP776 in an additional H-bond. Because 1M17 represents an inactive-like conformation of EGFR, no inference regarding selectivity for the active conformation is drawn from this pose. The fluorine and chlorine substituents participated in halogen bonding, and ILE765 further contributed through a halogen bond interaction. Charged residue contacts from LYS721, ARG817, and LYS851 (positive) and ASP813, GLU738, and ASP776 (negative) provided additional electrostatic stabilization.

In comparison, the reference drug methotrexate (−5.956 kcal mol^−1^) formed H-bonds with ASP831 and ASP776, a salt bridge *via* LYS692, and hydrophobic contacts with a narrower residue set, reflecting its lower binding efficiency. The thioamide analogue 6b (−5.956 kcal mol^−1^) and the dimethyl-pyrimidine derivative 12b (−5.228 kcal mol^−1^) showed the weakest affinities, likely due to reduced complementarity with the hydrophobic pocket and fewer polar anchoring interactions.

Compounds 8a and 8b (containing a quinazoline–aniline–chloro scaffold) showed strong binding (−6.788 and −7.587 kcal mol^−1^, respectively), with halogen bonding by the chloro substituents contributing significantly. Compound 8b additionally exhibited a pi–cation interaction with LYS721, a pharmacologically important contact reported in several EGFR inhibitor series. Compounds of series 12 (12a–c) generally showed moderate binding affinities (−5.228 to −6.598 kcal mol^−1^), possibly due to the larger, more flexible pyrimidine–aminoquinazoline extension that may introduce entropic penalties upon binding (Fig. 2 and 3).

**Fig. 2:**
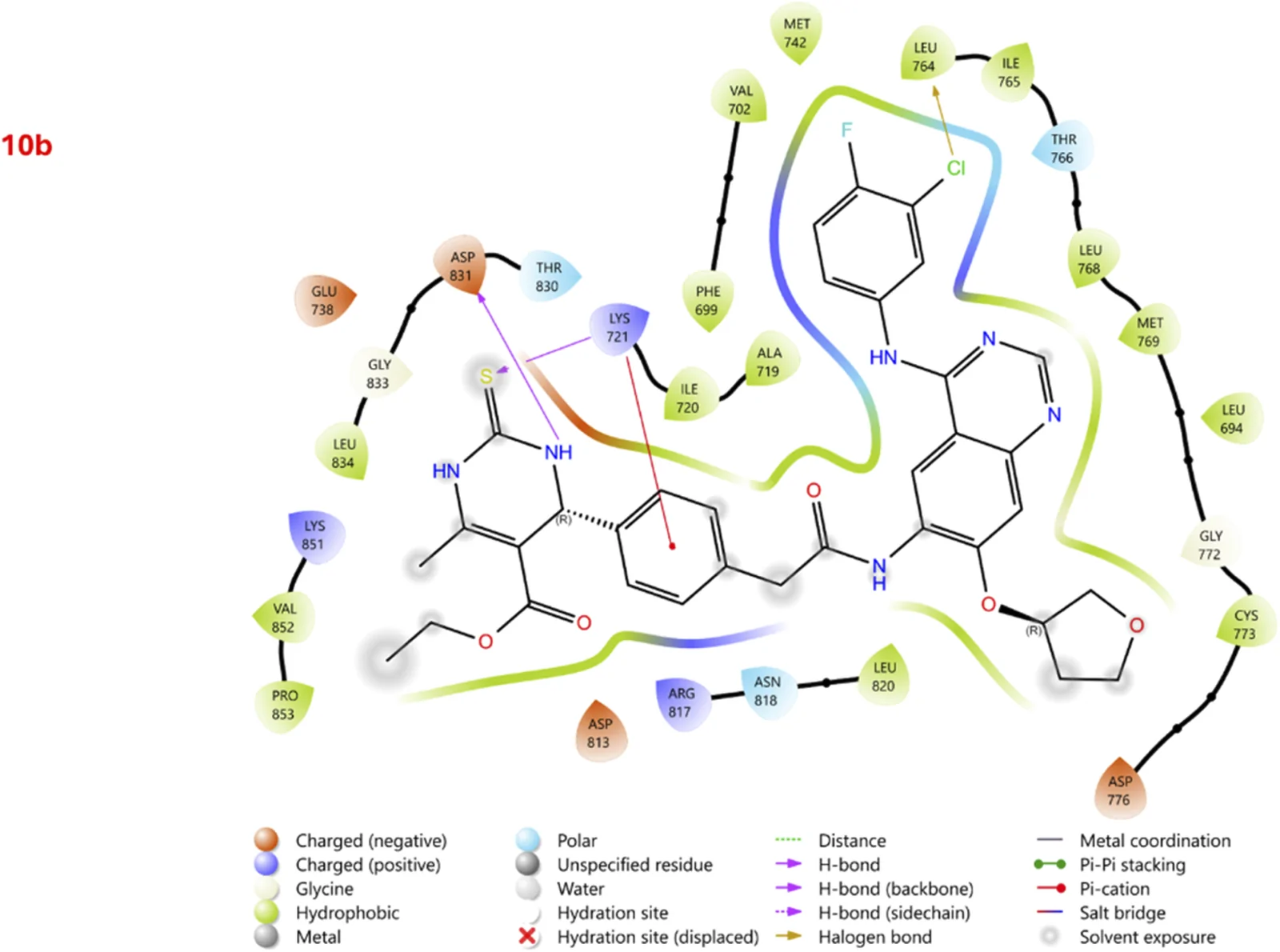
Two-dimensional binding interactions of compound 10b with the active site of protein 1M17.

**Fig. 3:**
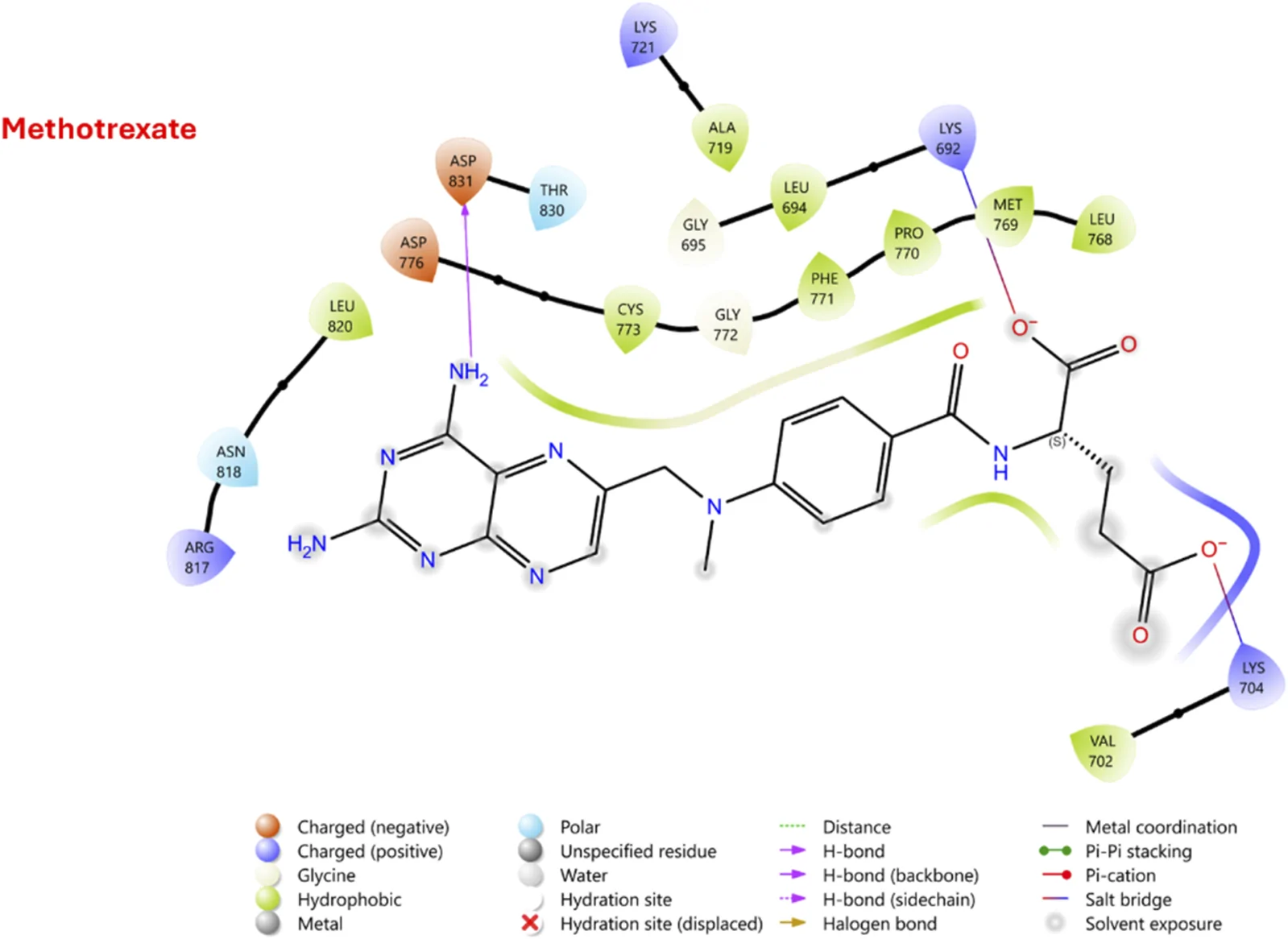
Two-dimensional binding interactions of the standard drug methotrexate with the active site of protein 1M17.

Choice of docking template and its limitations. PDB 1M17 was selected because it is co-crystallized with erlotinib, a 4-anilinoquinazoline EGFR-TKI that shares its core pharmacophore with the series 10 fragment, making it a reasonable structural benchmark for this chemotype. It is acknowledged, however, that 1M17 represents an inactive (DFG-out-like) conformation of EGFR, and that the synthesized compounds differ from erlotinib in scaffold extension and linker flexibility; the present docking results should therefore be read as an indication of chemotype compatibility with the erlotinib-binding pocket rather than definitive proof of an active-site, ATP-competitive inhibition mode. A full re-docking campaign against active-conformation structures (*e.g.*, PDB 2GS6 or 3POZ) to evaluate potential Type-I/Type-II binding modes was beyond the scope of the present revision and is identified as a direction for follow-up work (see Conclusion).

### Normal mode analysis (NMA) study

To validate the stability and dynamic behavior of the highest-affinity complex, normal mode analysis (NMA) based on an elastic network model was performed on the 10b–EGFR (1M17) complex. The NMA was performed using the ProDy-based elastic network model as implemented in the iMODS server. The analysis yielded six complementary plots that collectively characterize the conformational flexibility, mechanical stability, and inter-residue communication of the complex.^16^

The deformability plot (Fig. 4) illustrates the capacity of each residue in the 10b–1M17 complex to undergo local conformational changes. The profile is largely flat (values ≈ 0.05–0.20) across the central region (atom indices ∼ 50–280), indicating a well-ordered and rigid active-site architecture around the bound ligand. Notable flexibility peaks are observed at the N-terminal region (indices 1–30), near index ∼ 110, ∼170, and most prominently at the C-terminal tail (indices 295–325, deformability approaching 1.0). These flexible termini are characteristic of surface-exposed loop regions and do not perturb the stable binding core. The restrained deformability in the active-site region (indices 130–230, corresponding to key binding residues THR830, ASP831, LYS721) confirms that ligand 10b rigidifies the catalytic cleft, a hallmark of high-affinity kinase inhibitor binding.

**Fig. 4:**
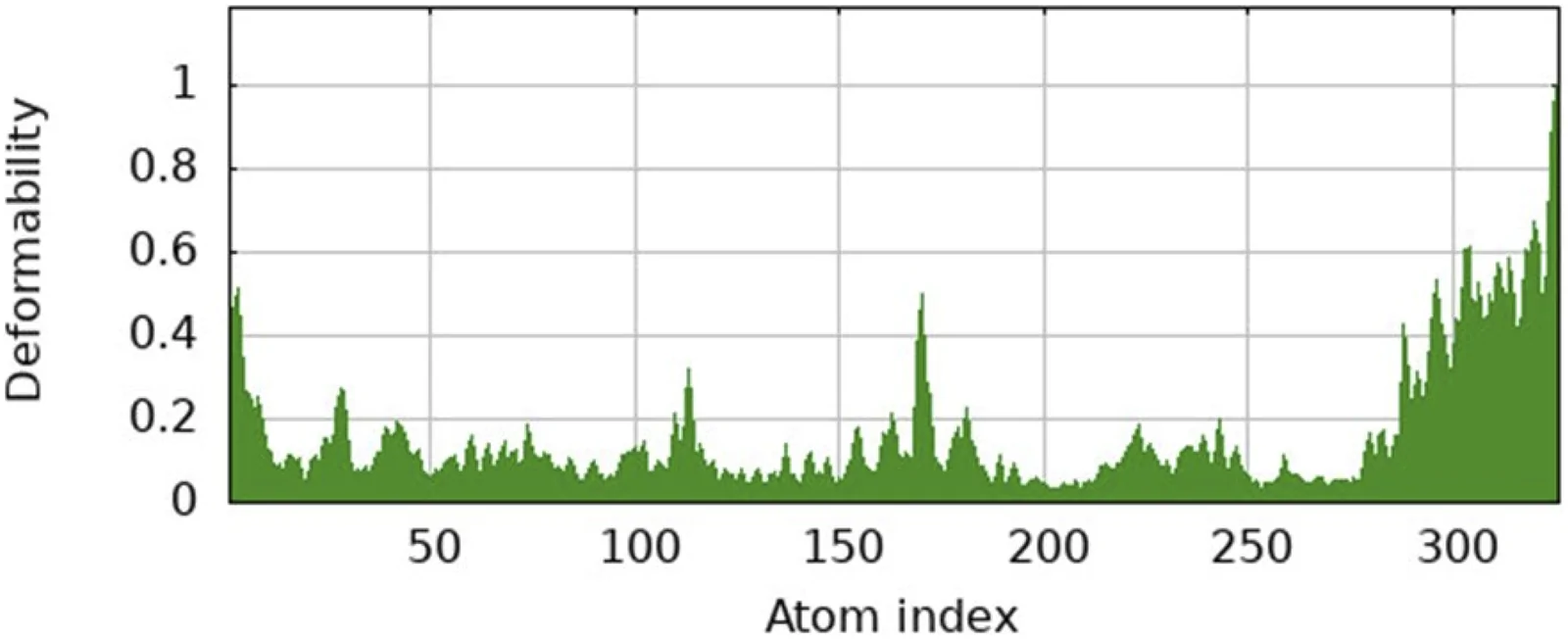
Deformability profile of the 10b–1M17 complex. Green peaks represent regions of high conformational flexibility along the polypeptide chain.

The *B*-factor plot (Fig. 5) compares the theoretical atomic mobility predicted by NMA (red overlay) against the experimentally determined crystallographic *B*-factors from the PDB structure (grey). The NMA-derived *B*-factors are consistently lower in magnitude and exhibit a smoother, more uniform distribution than the experimental values, reflecting the global averaging intrinsic to elastic network models. Critically, both profiles exhibit correlated flexibility patterns: elevated mobility at the N-terminus and C-terminal tail, with relatively suppressed fluctuations in the core binding domain (indices 50–290). The experimental *B*-factors show several sharp peaks (near indices 45–80, 100, 155–165, 175–180, and 200), likely corresponding to loop regions and flexible helix hinges within the kinase domain. The good qualitative agreement between NMA and PDB *B*-factors validates the elastic network model and demonstrates that the 10b-bound form of 1M17 retains structural features consistent with the crystal structure, supporting the physiological relevance of the docking pose.

**Fig. 5:**
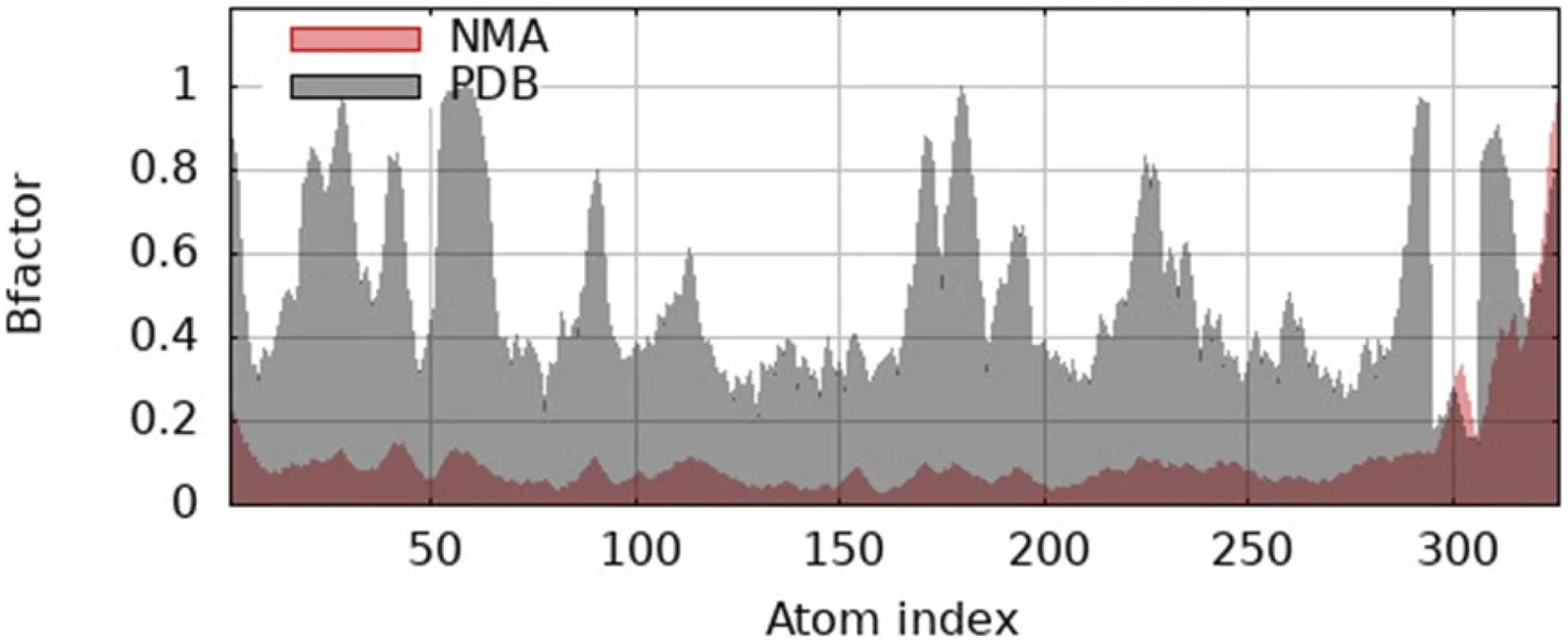
*B*-Factor comparison between NMA-predicted values (red) and experimental crystallographic PDB *B*-factors (grey) for the 10b–1M17 complex.

The eigenvalue plot (Fig. 6, left) displays the relative stiffness of the first 20 non-trivial normal modes as a ratio normalized to the first eigenvalue (eigenvalue (1) = 8.679655 × 10^−6^). The lowest-frequency modes (modes 1–3) possess the smallest eigenvalues, reflecting the softest, most collectively accessible motions of the complex—these represent large-scale domain movements that are biologically relevant for conformational transitions associated with kinase activation. The progressive increase in eigenvalues with mode index confirms a continuous stiffening of vibrational modes, which is typical of well-folded globular proteins. The small magnitude of the first eigenvalue (8.68 × 10^−6^) indicates that the global motion is energetically facile, consistent with the physiological requirement for EGFR to undergo conformational switching between active and inactive states.

**Fig. 6:**
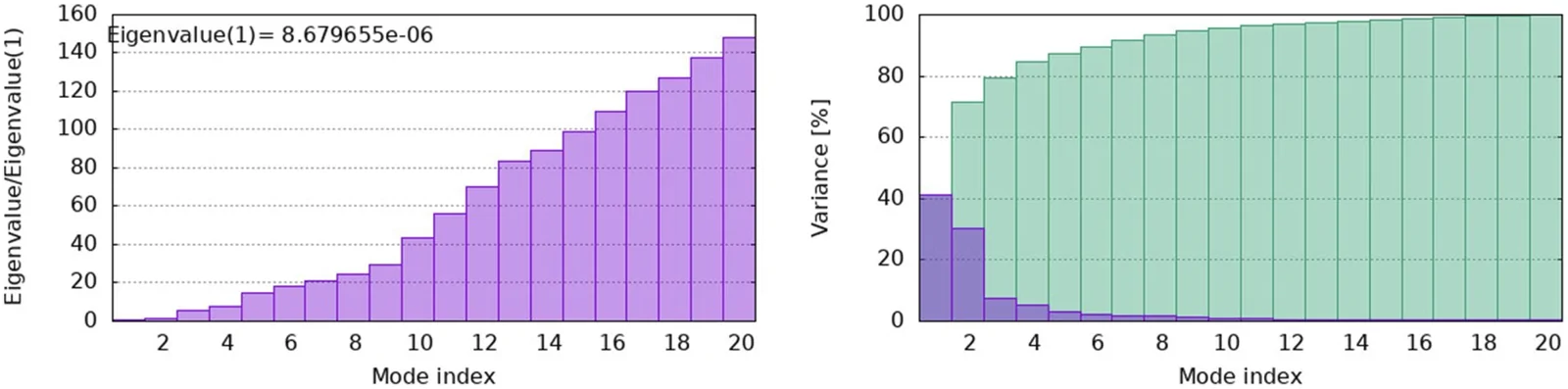
(Left) Eigenvalue ratio plot showing relative stiffness of the first 20 normal modes. Eigenvalue (1) = 8.679655 × 10^−6^. (Right) Variance plot showing individual (purple) and cumulative (teal) variance contributed by each mode.

The variance plot (Fig. 6, right) quantifies the contribution of each normal mode to total conformational variance. Mode 1 alone accounts for approximately 41% of the total variance (purple bar), underscoring its dominant role in describing the complex's principal motion. Mode 2 contributes ∼30%, and together the first two modes capture ∼71% of the total variance. The cumulative variance (teal bars) reaches ∼85% by mode 4 and approaches 100% by mode 10, indicating that the essential dynamics of the 10b–1M17 complex are captured in a low-dimensional subspace. This concentration of variance in the lowest modes reflects a well-structured, compliant binding complex, where 10b occupies the active site without disrupting the fundamental dynamic architecture of the enzyme.

The dynamic cross-correlation map (DCCM; Fig. 7, left) presents the pairwise correlation of residue fluctuations derived from the normal mode ensemble. The prominent red diagonal band confirms strong self-correlation of adjacent residues, as expected. Notably, several off-diagonal red blocks are observed: a strong correlation between residues 0–80 and 0–80 (N-terminal domain) suggests coherent motion within the β-sheet core; positive correlations between the regions 130–200 and 130–200 (catalytic loop and activation loop) indicate that these functionally critical segments move in concert, a key feature of productive substrate engagement. Regions flanking the active site (indices ∼ 100–130 and ∼200–250) show marked anti-correlation (blue), reflecting the counter-directional hinge motion between the N- and C-terminal lobes of the kinase a well-established mechanism underlying ATP binding and release. The suppression of anti-correlated motion in the core binding region (∼150–200) where 10b anchors through its multiple hydrogen bonds and halogen contacts suggests that the ligand imposes conformational restraint on loop dynamics, which may contribute to kinase inhibition; because 1M17 represents an inactive-conformation template, no inference regarding the DFG conformational state is drawn.

**Fig. 7:**
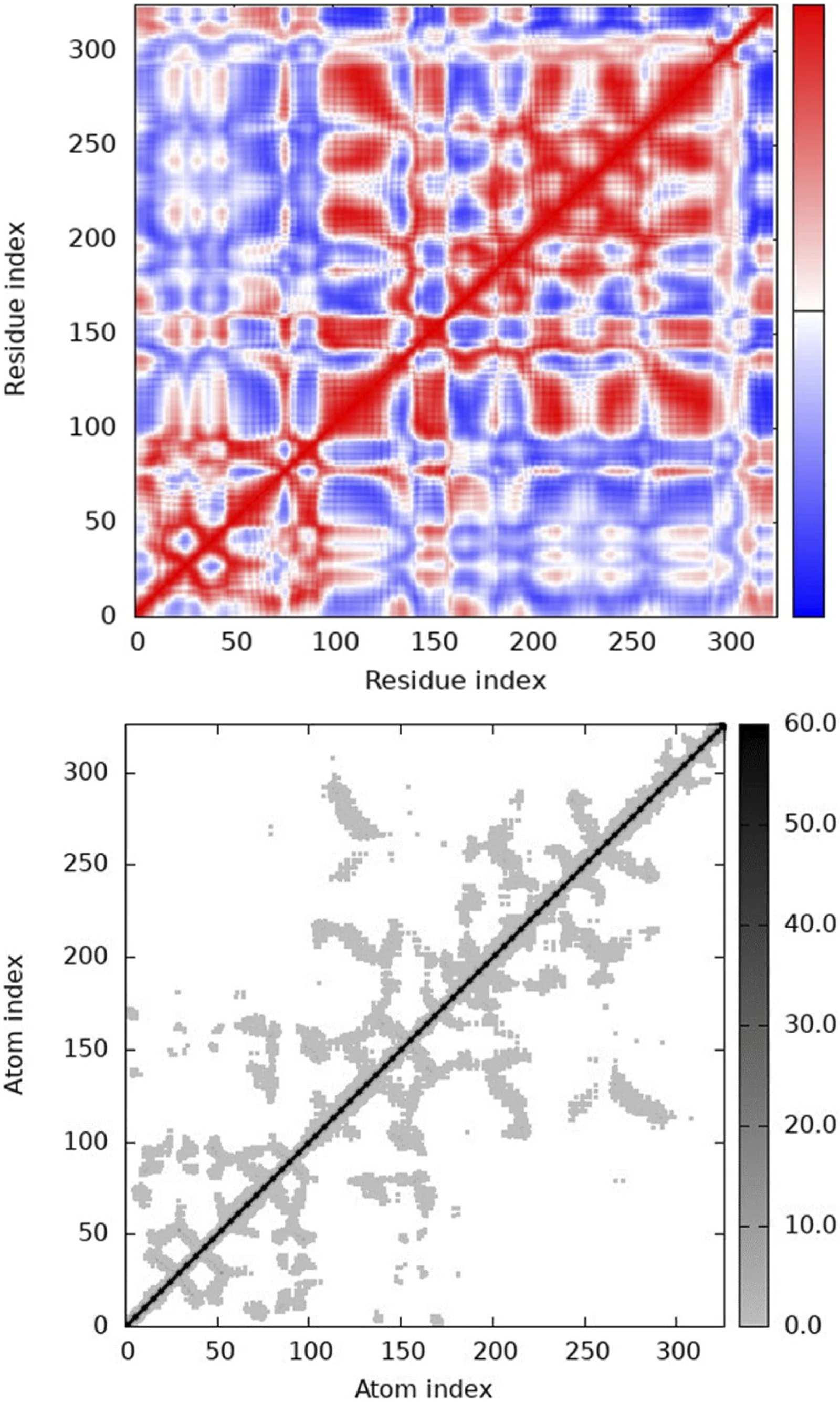
(Left) Dynamic cross-correlation map (DCCM) of the 10b–1M17 complex. Red regions indicate correlated motion; blue regions indicate anti-correlated motion. (Right) Elastic network contact map showing inter-residue contacts within a distance cutoff of 60 Å (darker = shorter distance).

The elastic network contact map (Fig. 7, right) displays inter-residue contacts within a 60 Å distance threshold, with darker shading indicating shorter interaction distances. The dense clustering of contacts along and near the main diagonal confirms the expected compact secondary structure connectivity. Several scattered off-diagonal contact clusters are evident, particularly between residues 0–100 and 200–325, indicating long-range tertiary contacts that form the structural scaffold of the kinase domain. These long-range contacts are essential for maintaining the rigid architecture of the ATP-binding cleft and are consistent with the experimental crystal structure of 1M17. The distribution pattern, with moderate off-diagonal contact density, is characteristic of a well-organized kinase fold and supports the notion that compound 10b does not induce significant structural disruption upon binding.

### Anticancer activity

All synthesized compounds were screened for cytotoxic activity against the A549 human lung carcinoma cell line using the MTT assay, with methotrexate (MTX) as the reference standard (IC_50_ = 15.00 ± 0.48 µg mL^−1^; 33.01 µM). IC_50_ values are reported here in µM (molar potency, calculated from each compound's molecular weight), alongside the originally determined µg mL^−1^ values in parentheses, since the synthesized analogs span a molecular weight range of ∼540–692 g mol^−1^ and molar units are required for a valid structure–activity comparison. IC_50_ values ranged from 23.31 to 202.20 µM (16.11–121.30 µg mL^−1^) across the series (Table 2). Compound 10b demonstrated the highest potency (IC_50_ = 16.11 ± 0.11 µg mL^−1^; 23.31 µM), exceeding the molar potency of the reference drug and all other analogs. This result is broadly consistent with its favorable docking score (−7.819 kcal mol^−1^) and the conformational stability of the 10b–EGFR complex indicated by NMA. It is noted, however, that the docking–activity relationship across the full series is only qualitative (see correlation analysis below); the computational data are therefore presented as supportive, rather than definitive, evidence for EGFR-mediated cytotoxicity.

**Table 2:** *In vitro* cytotoxic activity (IC_50_, µM; µg mL^−1^ in parentheses) of synthesized compounds against A549 human lung carcinoma cell line^a^

Compound	IC_50_ (µM)	IC_50_ (µg mL^−1^)	MW (g mol^−1^)
6a	64.63	35.00	541.54
6b	146.34	81.60	557.61
6c	47.23	26.24	555.58
8a	76.48	49.26	644.09
8b	183.75	121.30	660.14
8c	40.22	26.47	658.13
10a	63.52	42.88	675.06
10b	23.31	16.11	691.12
10c	31.29	21.56	689.04
12a	202.20	116.80	577.65
12b	43.51	25.83	593.66
12c	181.35	107.30	591.67
Methotrexate	33.01	15.00	454.41

a Values are expressed as mean ± SD (*n* = 3). Highlighted in green: most potent synthesized compound (10b). Highlighted in yellow: reference standard (methotrexate). Lower IC_50_ = greater cytotoxic potency.

Choice of cytotoxicity reference standard. Methotrexate (MTX) was used as the cytotoxicity reference because it is a well-established and widely used positive control in MTT assays against A549 cells and enables comparison with previous DHPM/Biginelli-hybrid anticancer studies that have employed MTX as a benchmark. However, MTX, a dihydrofolate reductase inhibitor, is not a mechanistically matched comparator for compounds investigated in the context of EGFR. Clinically approved EGFR tyrosine kinase inhibitors (EGFR-TKIs), such as gefitinib or erlotinib, would provide a more appropriate reference for evaluating EGFR-related cellular activity. Accordingly, the present results are interpreted without attributing EGFR selectivity based solely on comparison with MTX, and direct comparison with an EGFR-TKI is proposed as an important direction for further validation.

Structure–activity relationships (SAR) revealed distinct trends across the four series. Within series 10, cytotoxic potency followed the order 10b > 10c > 10a, with IC_50_ values of 23.31, 31.29, and 63.52 µM, respectively (16.11, 21.56, and 42.88 µg mL^−1^). The enhanced activity of 10b, bearing a C2-thione functionality and an (*S*)-tetrahydrofuran-3-yl ether linker, was consistent with its favorable predicted interactions within the EGFR binding pocket, including hydrogen-bonding interactions involving ASP831 and LYS721. However, these computational interactions should be considered supportive rather than definitive evidence of the mechanism of cytotoxicity.

In series 8, compound 8c exhibited the highest potency (IC_50_ = 40.22 µM, 26.47 µg mL^−1^), followed by 8a (76.48 µM, 49.26 µg mL^−1^), whereas 8b was markedly less potent (183.75 µM, 121.30 µg mL^−1^). Thus, incorporation of the C2-thione functionality in the quinoline–DHPM framework did not translate into improved cellular activity for 8b, despite its favorable docking score. This discrepancy indicates that factors beyond predicted target affinity, including cellular permeability, solubility, efflux, or metabolic stability, may contribute to the observed cytotoxic response.

Series 6 showed that *N*-methylation improved activity, with 6c (IC_50_ = 47.23 µM, 26.24 µg mL^−1^) being more potent than the parent ketone 6a (64.63 µM, 35.00 µg mL^−1^) and the corresponding thione 6b (146.34 µM, 81.60 µg mL^−1^). This trend suggests that modification of the DHPM core can substantially influence cellular potency, although the precise contribution of these structural changes requires further mechanistic investigation.

Series 12 displayed comparatively weaker activity overall, particularly for 12a (202.20 µM, 116.80 µg mL^−1^) and 12c (181.35 µM, 107.30 µg mL^−1^), whereas 12b showed moderate activity (43.51 µM, 25.83 µg mL^−1^). The lower potency of 12a and 12c was accompanied by less favorable predicted binding interactions, although cellular factors may also contribute to their reduced activity.

Overall, the relationship between docking score and cytotoxic potency was further examined quantitatively. All twelve synthesized compounds were docked and screened, so the correlation analysis was performed on the complete set (*n* = 12). Pearson correlation between docking score and *p*IC_50_ gave *r* = −0.219 (*p* = 0.494), indicating a weak and statistically nonsignificant correlation; rank correlation gave the same picture (Spearman *ρ* = −0.217, *p* = 0.499). Among compounds with docking scores more negative than −6.5 kcal mol^−1^, 10b, 6c, and 10c showed IC_50_ values below 50 µM, whereas 8b represented a notable exception, exhibiting a favorable docking score (−7.587 kcal mol^−1^) but substantially weaker cytotoxicity (IC_50_ = 183.75 µM). Exclusion of 8b as an outlier modestly increased the correlation (*n* = 11, *r* = −0.475, *p* = 0.139), but the relationship remained statistically nonsignificant. These findings indicate that docking scores alone are insufficient to predict cellular potency and that additional pharmacokinetic and cellular factors may influence the biological response.

Among the synthesized compounds, 10b emerged as the most promising candidate, combining the highest cytotoxic potency (IC_50_ = 23.31 µM; 16.11 µg mL^−1^) with favorable computational characteristics. Nevertheless, the present findings support its consideration as a candidate for further optimization and mechanistic investigation, rather than as a validated EGFR-targeted agent. Direct biochemical EGFR kinase inhibition studies and complementary mechanistic investigations will be important to establish the contribution of EGFR to its observed cytotoxic activity.

### DFT study

#### DFT-based computational study of compound 10b

Density Functional Theory (DFT) calculations were performed on compound 10b at the B3LYP/6-311G(d,p) level of theory using Gaussian 09 to investigate its molecular geometry, electronic charge distribution, frontier molecular orbital (FMO) characteristics, global chemical reactivity descriptors, and molecular electrostatic potential (MEP). These quantum chemical parameters describe the intrinsic electronic properties of the isolated molecule in the gas phase. They are reported here as physicochemical characterisation; because they do not model the solvated protein–ligand system, the cell membrane, or any cellular process, they are not interpreted as causes or predictors of the measured cytotoxicity of 10b.^17^

### Optimized molecular geometry

The fully optimized 3D geometry of compound 10b (Fig. 8) reveals a multi-fragment, non-planar architecture comprising a central dihydropyrimidine-2(1*H*)-thione (DHPM-S) scaffold bearing an ethyl ester at C5, a 4-substituted phenyl ring at C4, and a lateral (*S*)-tetrahydrofuran-3-yl ether linker tethering a quinazoline moiety that terminates in a 4-fluoro-2-chloroanilino head group. The Biginelli condensation is not stereoselective, so the DHPM C4 centre is formed as a racemate; the (*R*)-C4 enantiomer was arbitrarily selected for calculation, while the (*S*)-configuration of the tetrahydrofuran-3-yl ether is defined by the enantiopure amine used in the coupling step and is retained throughout. The DHPM ring adopts a slightly puckered half-chair conformation. The C=S bond length (∼1.67 Å) at C2 is consistent with partial double-bond character and with the thione NH acting as an H-bond donor. The dihedral angle (∼55°) between the DHPM ring and the pendant phenyl ring is reported as a geometric parameter only. The C–F (∼1.34 Å) and C–Cl (∼1.75 Å) bond lengths are consistent with standard halogen-bond donor geometry, which is compatible with, but does not by itself establish, the halogen contacts to ILE765 and LEU764 suggested by docking.

**Fig. 8:**
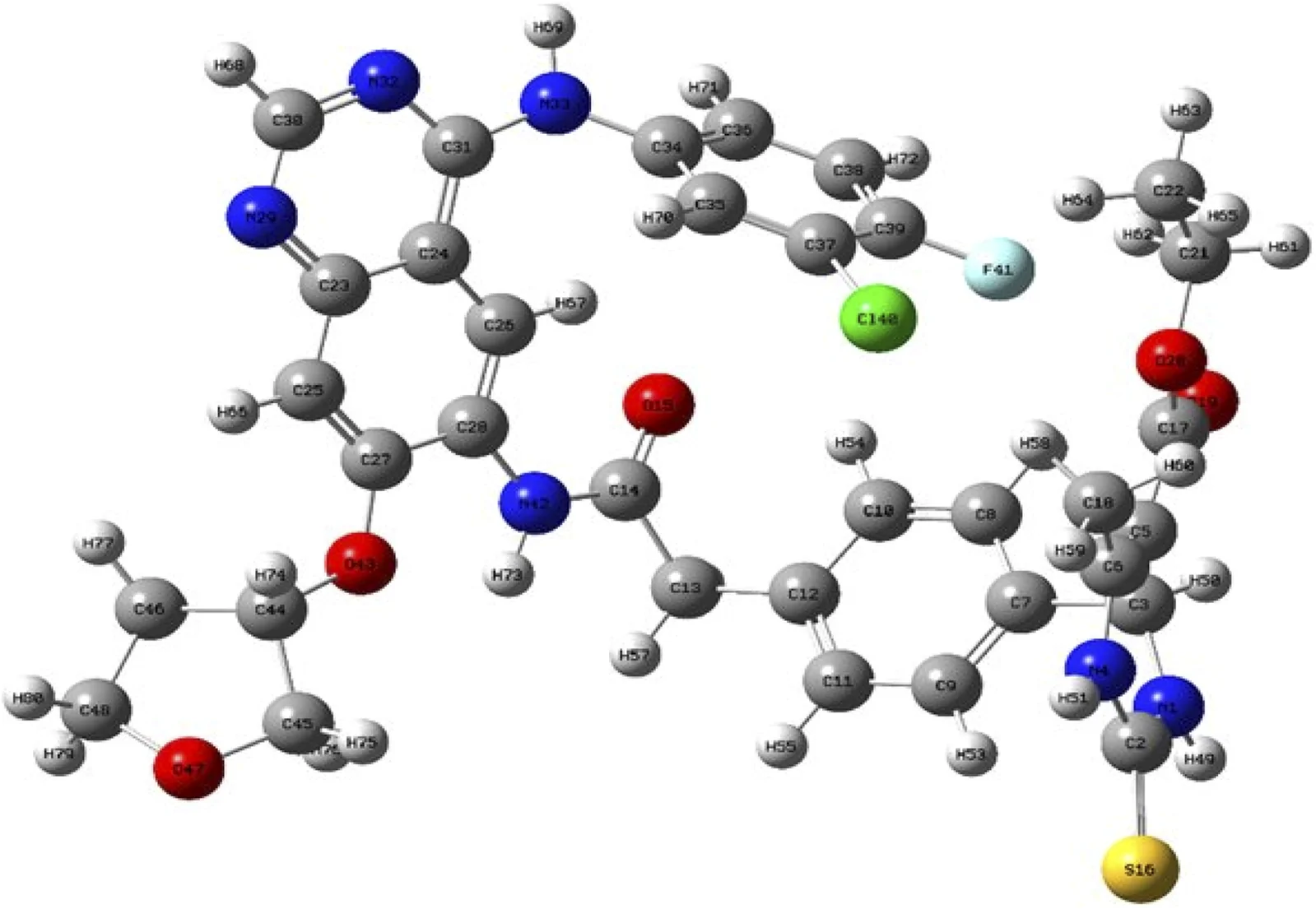
DFT-optimized molecular geometry of compound 10b at the B3LYP/6-311G(d,p) level. Color code: gray = C, blue = N, red = O, yellow = S, green = Cl, cyan = F, white = H.

### Mulliken atomic charge distribution

The Mulliken charge analysis (Fig. 9) maps the electron density over all 80 atoms of compound 10b and pinpoints key reactive centers. The DHPM nitrogen atoms (N1, N3) and quinazoline nitrogens (N29, N33) carry significant negative partial charges (−0.5 to −0.7*e*), confirming their roles as H-bond acceptors and nucleophilic sites that interact with electropositive EGFR residues LYS721 and ARG817. The C2 thione carbon bears a high positive charge (∼+0.58*e*), reflecting polarization by the flanking electronegative sulfur (S16, ∼−0.45*e*) and ring nitrogen making it electrophilic and reinforcing the H-bond donor character of the adjacent NH toward ASP831. The carbonyl carbon C14 of the amide linker (∼+0.60*e*) is similarly electrophilic. Oxygen atoms (O15, O19, O20, charges −0.4 to −0.6*e*) serve as H-bond acceptors, while F41 (−0.25*e*) and Cl40 display moderate negative charges consistent with their electron-withdrawing inductive effects and halogen-bonding capacity. This charge distribution is internally consistent with the MEP analysis. It is described here as an electronic feature of the isolated molecule and is not presented as an explanation of the cellular activity of 10b and the EGFR binding pocket.

**Fig. 9:**
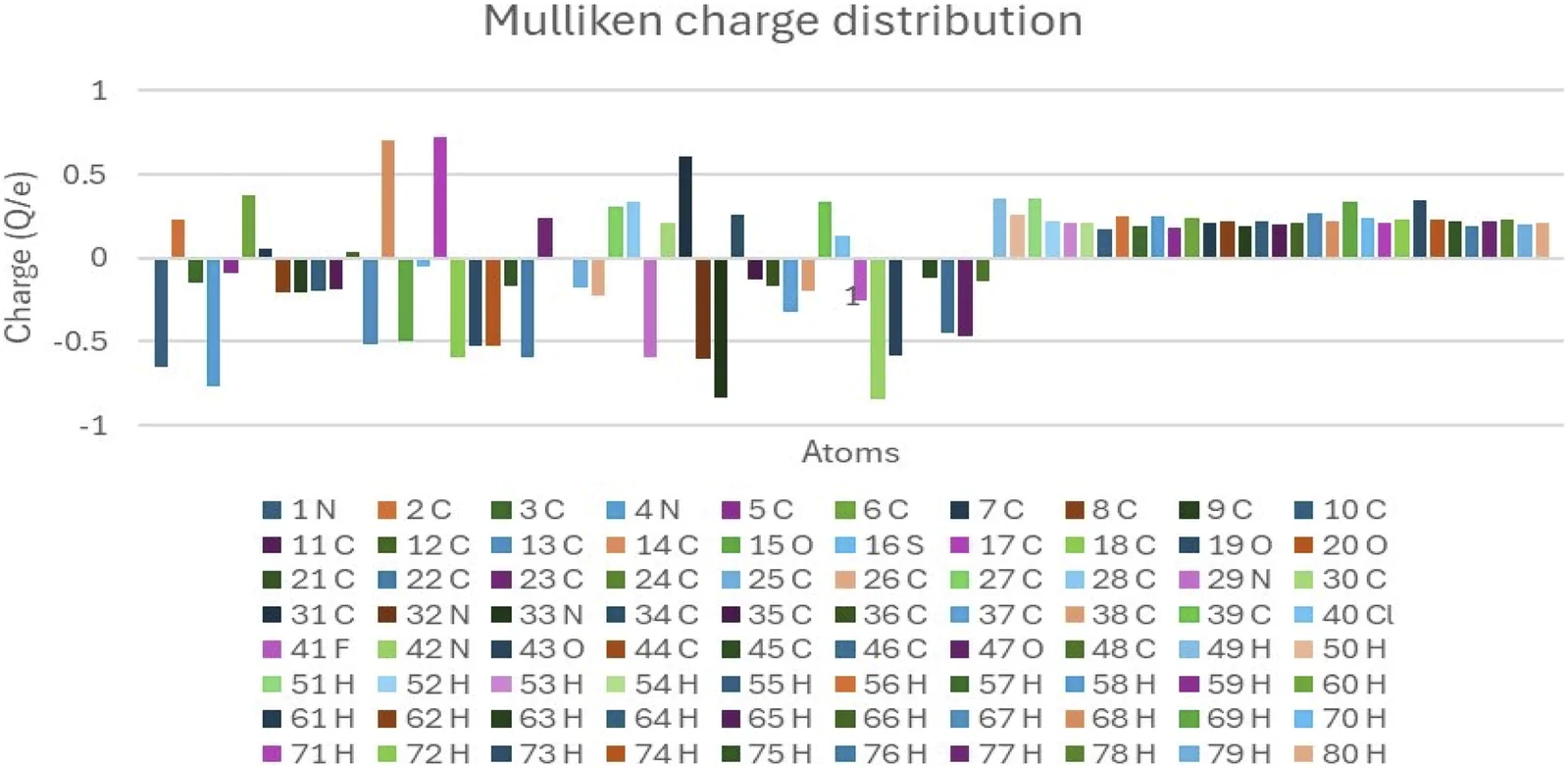
Mulliken atomic charge distribution of compound 10b. Positive bars indicate electron-deficient (electrophilic) centers; negative bars indicate electron-rich (nucleophilic) sites.

### Frontier molecular orbitals and global reactivity descriptors

The FMO analysis (Fig. 10, Table 3) reveals *E*_HOMO_ = −5.5960 eV and *E*_LUMO_ = −1.5510 eV, giving a HOMO–LUMO gap (Δ*E*) of 4.045 eV. The HOMO is predominantly delocalized over the DHPM thione ring and the flanking aromatic system, the same fragments responsible for the π–cation interaction with LYS721 and H-bond donation to ASP831 in docking; electron donation in 10b therefore originates from these π-rich regions. The LUMO is localized on the 4-fluoro-2-chloroanilino moiety and adjacent carbonyl, identifying this as the electron-accepting region of the molecule. The moderate Δ*E* of 4.045 eV is consistent with a kinetically stable molecule; no inference about receptor binding or cellular potency is drawn from this value.

**Fig. 10:**
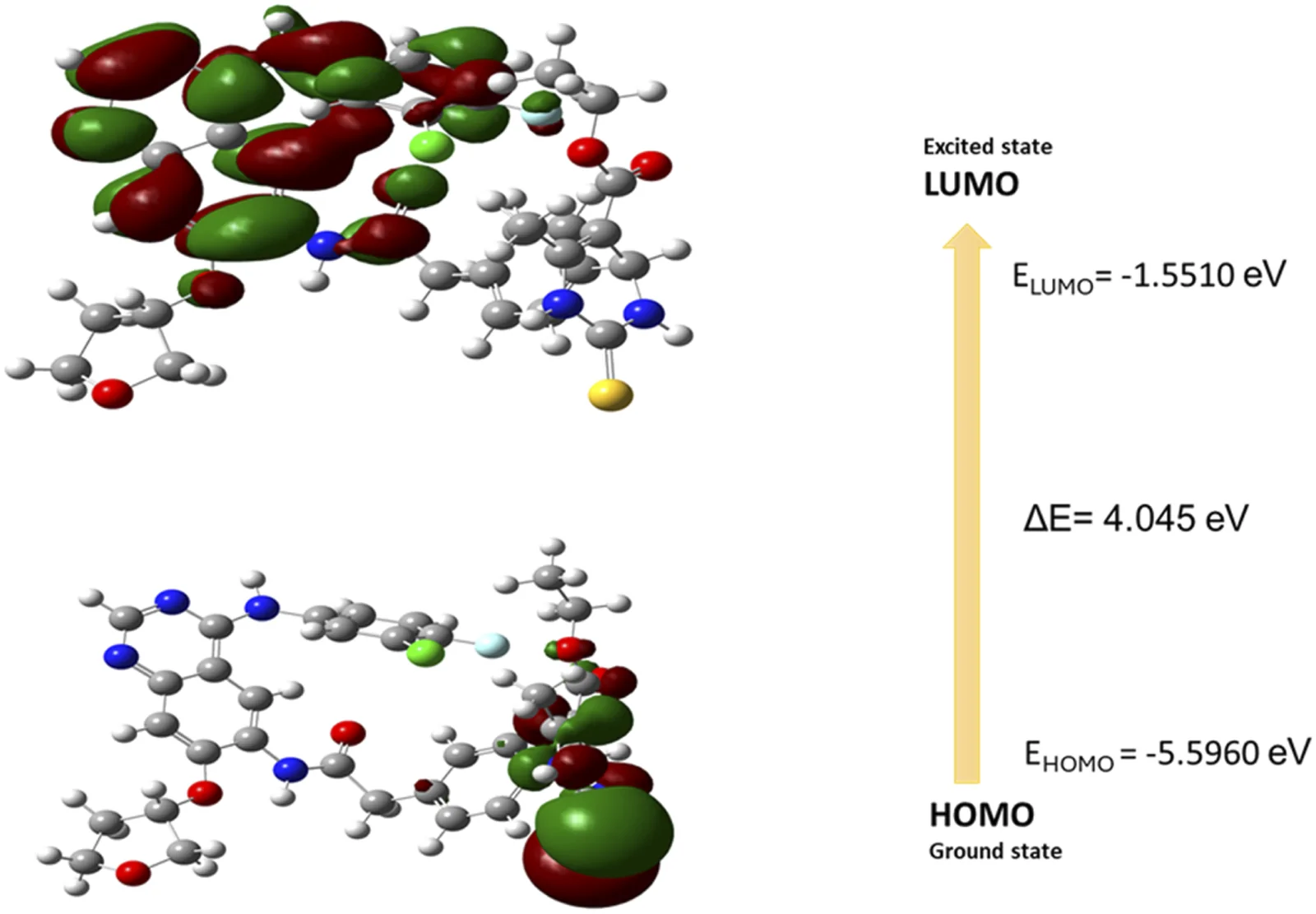
Frontier molecular orbital plots of compound 10b: HOMO (top) and LUMO (bottom). Green and red lobes represent positive and negative orbital phases, respectively.

**Table 3:** Frontier orbital energies and global chemical reactivity descriptors of compound 10b at B3LYP/6-311G(d,p)^a^

Descriptor	Formula	Compound 10b (eV)
**Frontier orbital energies**		
*E*_HOMO_	—	−5.5960
*E*_LUMO_	—	−1.5510
Δ*E*	*E*_LUMO_ − *E*_HOMO_	4.045

**Global reactivity descriptors**		
Ionization potential (*I*)	−*E*_HOMO_	5.5960
Electron affinity (*A*)	−*E*_LUMO_	1.5510
Electronegativity (*χ*)	(*I* + *A*)/2	3.5735
Chemical potential (*µ*)	−*χ*	−3.5735
Hardness (*η*)	(*I* − *A*)/2	2.022
Softness (*S*)	1/*η*	0.4945
Electrophilicity index (*ω*)	*µ* ^2^/2*η*	3.155
Dipole moment (*µ*_D_)	—	5.1394

a All energies in eV. HOMO = highest occupied molecular orbital; LUMO = lowest unoccupied molecular orbital; Δ*E* = HOMO − LUMO energy gap.

The global reactivity descriptors (Table 3) further characterize the chemical behaviour of 10b. The ionization potential (*I* = 5.596 eV) reflects moderate electron-donating capacity from the HOMO-resident sulfur and nitrogen atoms. The electron affinity (*A* = 1.551 eV) reflects the electron-accepting ability of the halogenated anilino head. The electronegativity (*χ* = 3.574 eV) and chemical potential (*µ* = −3.574 eV) indicate a thermodynamic drive for electron density transfer from 10b toward the EGFR binding site. The chemical hardness (*η* = 2.022 eV) and softness (*S* = 0.4945 eV^−1^) together indicate that 10b is a moderately polarizable molecule capable of adapting its electron density upon induced-fit binding in the flexible EGFR active site. Critically, the electrophilicity index (*ω* = 3.155 eV) classifies 10b as a strong electrophile on the Domingo scale (*ω* > 1.5 eV). The dipole moment (5.1394 D) reflects the molecular polarity arising from the thione, ester, and halogenated aniline groups. These are gas-phase descriptors of the isolated molecule; they are not used here to infer charge-transfer interactions with specific EGFR residues, membrane permeability, or cellular potency, none of which were measured in this work.

### Molecular electrostatic potential (MEP) analysis

The MEP maps (Fig. 11) visually integrate the charge distribution results and provide a direct electrostatic pharmacophore of compound 10b. The deep red regions (most negative potential) concentrated over the thione sulfur (S16), quinazoline nitrogen atoms, and ester oxygens identify the primary nucleophilic sites and H-bond acceptors regions that engage with electropositive residues LYS721, ASP831, and LYS851 in the EGFR pocket. Blue regions (positive potential) over the DHPM ring NH and amide NH confirm their H-bond donor character toward the hinge residue ASP831, fully consistent with the docking interactions. Green/yellow intermediate regions covering the chlorofluoroanilino ring and aromatic core represent hydrophobic and halogen-bonding zones, consistent with the 15-residue hydrophobic envelope (LEU768, MET769, LEU764, PHE699, *etc.*) and the halogen bond to ILE765 observed in molecular docking. Overall, the MEP surface of 10b defines the electron-rich and electron-poor faces of the molecule. These features are qualitatively compatible with the electrostatic environment of the modelled binding site, but the MEP was computed for the isolated molecule and provides no causal explanation for either the docking score or the measured IC_50_; the concordance is presented as descriptive rather than predictive.

**Fig. 11:**
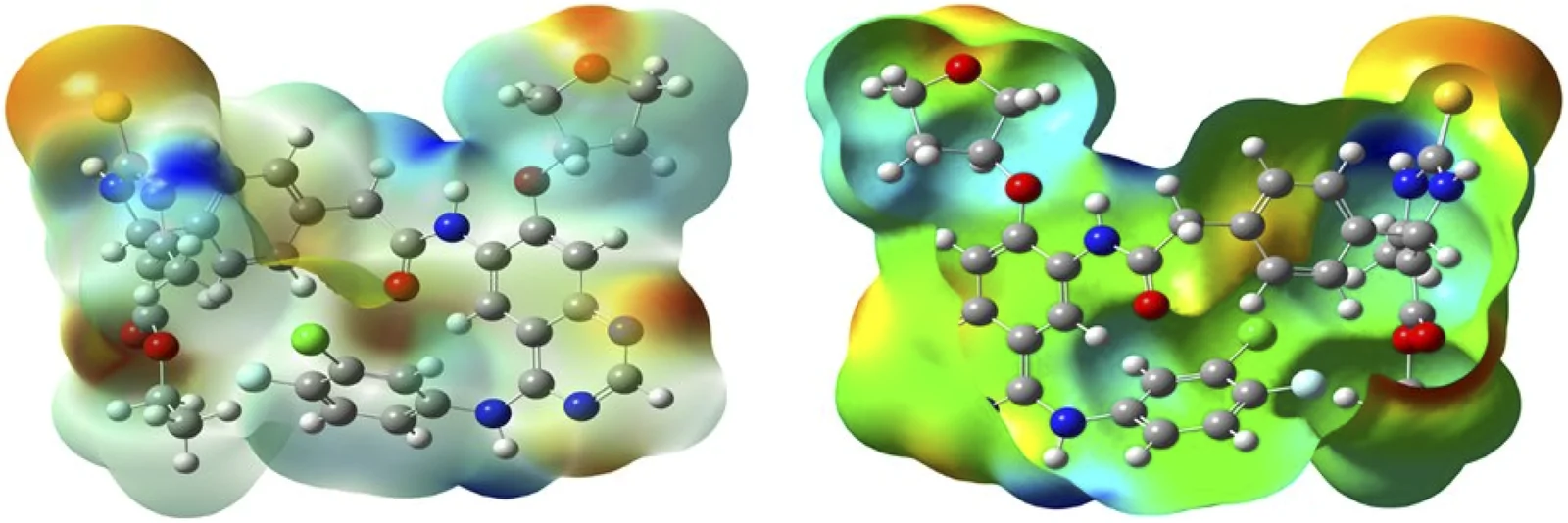
Molecular electrostatic potential (MEP) maps of compound 10b mapped onto the electron density isosurface. Left: standard MEP; right: enhanced color range. Red = electron-rich (nucleophilic); blue = electron-poor (electrophilic); green/yellow = intermediate.

## Experimental section

### General information

All chemicals and reagents were procured from Sigma-Aldrich, Alfa Aesar, and Spectrochem and used as received unless otherwise stated. Solvents were of analytical reagent (AR) grade. Melting points were determined in open capillary tubes using a digital melting point apparatus and are uncorrected. Reaction progress was monitored by TLC on precoated silica gel 60 F254 aluminium-backed plates (Merck), with visualization under UV light at 254 nm. Column chromatography was performed using silica gel (60–120 mesh, Qualigens) with ethyl acetate/hexane as the eluent.

IR spectra were recorded using a PerkinElmer Spectrum Two ATR-FTIR spectrophotometer and are reported in cm^−1^. ^1^H and ^13^C NMR spectra were recorded on a Bruker Avance 400 MHz spectrometer at 400 and 100 MHz, respectively, using DMSO-*d*_6_ as solvent and TMS as the internal standard. Chemical shifts (*δ*) are reported in ppm and coupling constants (*J*) in Hz. The abbreviations s, d, t, q, and m denote singlet, doublet, triplet, quartet, and multiplet, respectively. ESI-MS spectra were recorded on a Waters Q-TOF Premier mass spectrometer in positive and negative ion modes and are reported as *m*/*z* values corresponding to [M + H]^+^ or [M − H]^−^ ions.

Theoretical monoisotopic masses of the molecular ions were calculated from the assigned molecular formulae and were consistent with the observed ESI-MS data. No numerical purity value is claimed for any compound. The identity and homogeneity of all final compounds are supported by the combined evidence of ATR-FTIR, ^1^H NMR, ^13^C NMR, and ESI-MS data, together with sharp, reproducible melting-point ranges and a single spot by TLC in at least two solvent systems. All original spectra are provided in the SI.

### General procedure for the synthesis of dihydropyrimidine–acetic acid intermediates (4a–c)

A mixture of 2-(4-formylphenyl)acetic acid (1, 1.0 equiv.), ethyl 3-oxobutanoate (ethyl acetoacetate, 1.2 equiv.), and urea, thiourea, or *N*′-methylurea (1.0 equiv.) was dissolved in glacial acetic acid (10 mL) and heated under reflux (110 °C) with stirring for 4 h. Reaction progress was monitored by TLC (ethyl acetate/hexane, 3 : 7 v/v). After cooling to room temperature, the mixture was poured onto crushed ice-water (50 mL). The precipitate was collected by vacuum filtration, washed with cold water (3 × 10 mL) and cold diethyl ether (10 mL), and dried at 60 °C in a hot-air oven. Recrystallization from ethanol gave the target dihydropyrimidine–acetic acid intermediates 4a (urea, off-white solid), 4b (thiourea, pale-yellow solid), and 4c (*N*′-methylurea, pale-yellow solid) in 68–78% yield.

### General procedure for the synthesis of dihydropyrimidine–1*H*-imidazole amide conjugates (6a–c)

To a stirred solution of the appropriate acid intermediate 4a, 4b, or 4c (1.0 equiv.) in anhydrous dichloromethane (DCM, 15 mL) cooled to 0 °C, *N*-(3-dimethylaminopropyl)-*N*′-ethylcarbodiimide hydrochloride (EDC·HCl, 1.5 equiv.) and 1-hydroxybenzotriazole (HOBt, 1.2 equiv.) were added sequentially. The mixture was stirred at 0 °C for 30 min to preactivate the carboxylic acid. Triethylamine (TEA, 2.0 equiv.) was then added dropwise, followed by 3-(4-methyl-1*H*-imidazol-1-yl)-5-(trifluoromethyl)aniline (1.0 equiv.). The mixture was warmed to room temperature and stirred under reflux for 12 h. Progress was monitored by TLC (ethyl acetate/hexane, 1 : 1 v/v). The mixture was diluted with DCM (30 mL), washed with saturated aqueous NaHCO_3_ (2 × 20 mL), water (20 mL), and brine (20 mL). The organic layer was dried over anhydrous Na_2_SO_4_, filtered, and concentrated under reduced pressure. Purification by column chromatography (silica gel 60–120 mesh; ethyl acetate/hexane gradient elution) afforded 6a (2-oxo, yellow solid), 6b (2-thioxo, yellow solid), and 6c (1,6-dimethyl-2-oxo, yellow solid) in 62–72% yield.

### General procedure for the synthesis of dihydropyrimidine–quinoline amide conjugates (8a–c)

To a stirred solution of 4a, 4b, or 4c (1.0 equiv.) in anhydrous DCM (15 mL) at 0 °C, EDC·HCl (1.5 equiv.) and HOBt (1.2 equiv.) were added and the mixture was stirred for 30 min for pre-activation. TEA (2.0 equiv.) was then added dropwise, followed by 4-(4-amino-3-chlorophenoxy)-7-methoxyquinoline-6-carboxamide (1.0 equiv.). The reaction mixture was stirred at reflux for 12 h and monitored by TLC (ethyl acetate/hexane, 1 : 1 v/v). Aqueous workup (saturated NaHCO_3_, water, brine) followed by drying (Na_2_SO_4_) and column chromatography (silica gel; ethyl acetate/hexane gradient) gave 8a (2-oxo, white solid), 8b (2-thioxo, white solid), and 8c (1,6-dimethyl-2-oxo, white solid) in 58–68% yield.

### General procedure for the synthesis of dihydropyrimidine–quinazoline amide conjugates (10a–c)

To a stirred solution of 4a, 4b, or 4c (1.0 equiv.) in anhydrous DCM (15 mL) at 0 °C, EDC·HCl (1.5 equiv.) and HOBt (1.2 equiv.) were added and stirred for 30 min. TEA (2.0 equiv.) and (*S*)-*N*^4^-(3-chloro-4-fluorophenyl)-7-((tetrahydrofuran-3-yl)oxy)quinazoline-4,6-diamine (1.0 equiv.) were added. The mixture was stirred at reflux for 12 h with TLC monitoring (ethyl acetate/hexane, 1 : 1 v/v). After standard aqueous workup and column chromatography (silica gel; ethyl acetate/hexane gradient), compounds 10a (2-oxo, brown solid), 10b (2-thioxo, brown solid), and 10c (1,6-dimethyl-2-oxo, brown solid) were obtained in 55–65% yield.

### General procedure for the synthesis of dihydropyrimidine–pyrimidine amide conjugates (12a–c)

To a stirred solution of 4a, 4b, or 4c (1.0 equiv.) in anhydrous DCM (15 mL) at 0 °C, EDC·HCl (1.5 equiv.) and HOBt (1.2 equiv.) were added and stirred for 30 min. TEA (2.0 equiv.) and 4-methyl-*N*^1^-(4-(pyridin-3-yl)pyrimidin-2-yl)benzene-1,3-diamine (1.0 equiv.) were added. The mixture was stirred at reflux for 12 h with TLC monitoring (ethyl acetate/hexane, 1 : 1 v/v). Workup and purification as described above furnished 12a (2-oxo, grey solid), 12b (2-thioxo, grey solid), and 12c (1,6-dimethyl-2-oxo, grey solid) in 60–70% yield.

### Spectral data

#### Ethyl 6-methyl-4-(4-(2-((3-(4-methyl-1*H*-imidazol-1-yl)-5-(trifluoromethyl)phenyl)amino)-2-oxoethyl)phenyl)-2-oxo-1,2,3,4-tetrahydropyrimidine-5-carboxylate (6a)

Yellow solid; mp 221–223 °C; molecular formula: C_27_H_26_F_3_N_5_O_4_, MW: 541.53. ^1^H NMR (400 MHz, DMSO-*d*_6_, *δ* ppm): 10.69 (1H, s, –CONH), 9.19 (1H, s, –NH), 8.16 (1H, s, –NH), 8.03 (1H, s, Ar–H), 7.91 (1H, s, Ar–H), 7.72 (1H, s, Ar–H), 7.66 (1H, s, Ar–H), 7.44 (1H, s, Ar–H), 7.28–7.30 (2H, d, *J* = 8 Hz, Ar–H), 7.18–7.20 (2H, d, *J* = 8 Hz, Ar–H), 5.13 (1H, s, –CH), 3.96–4.01 (2H, q, –CH_2_), 3.67 (2H, s, –CH_2_), 2.24 (3H, s, –CH_3_), 2.16 (3H, s, –CH_3_), 1.16 (3H, t, –CH_3_). ^13^C NMR (100 MHz, DMSO-*d*_6_, *δ* ppm): 168.06, 165.49, 144.33, 137.77, 135.27, 129.37, 129.31, 127.82, 124.99, 123.72, 122.46, 121.46, 40.07, 34.47, 11.06. IR (ATR): *ν̃* = 3453, 3314, 3063, 2982, 2930, 1703, 1663, 1601, 1497, 1335, 1167, 1095, 1007, 845, 706 cm^−1^. MS (ESI^+^): *m*/*z* 542.15 [M + H]^+^.

#### Ethyl 6-methyl-4-(4-(2-((3-(4-methyl-1*H*-imidazol-1-yl)-5-(trifluoromethyl)phenyl)amino)-2-oxoethyl)phenyl)-2-thioxo-1,2,3,4-tetrahydropyrimidine-5-carboxylate (6b)

Yellow solid; mp 228–230 °C; molecular formula: C_27_H_26_F_3_N_5_O_3_S, MW: 557.59. ^1^H NMR (400 MHz, DMSO-*d*_6_, *δ* ppm): 10.67 (1H, s, –CONH), 10.32 (1H, s, –NH), 9.64 (1H, s, –NH), 8.16 (1H, s, Ar–H), 8.03 (1H, s, Ar–H), 7.90 (1H, s, Ar–H), 7.66 (1H, s, Ar–H), 7.44 (1H, s, Ar–H), 7.30–7.32 (2H, d, *J* = 8 Hz, Ar–H), 7.16–7.19 (2H, d, *J* = 8 Hz, Ar–H), 5.15 (1H, s, –CH), 3.99–4.04 (2H, q, –CH_2_), 3.68 (2H, s, –CH_2_), 2.28 (3H, s, –CH_3_), 2.16 (3H, s, –CH_3_), 1.10–1.13 (3H, t, –CH_3_). ^13^C NMR (100 MHz, DMSO-*d*_6_, *δ* ppm): 168.06, 165.49, 144.33, 135.53, 132.28, 129.23, 128.63, 128.32, 124.99, 122.21, 40.07, 34.47, 11.06. IR (ATR): *ν̃* = 3446, 3311, 3059, 2981, 2921, 1702, 1660, 1598, 1495, 1335, 1198, 1165, 1095, 1007, 843, 700 cm^−1^. MS (ESI^+^): *m*/*z* 558.30 [M + H]^+^.

#### Ethyl 1,6-dimethyl-4-(4-(2-((3-(4-methyl-1*H*-imidazol-1-yl)-5-(trifluoromethyl)phenyl)amino)-2-oxoethyl)phenyl)-2-oxo-1,2,3,4-tetrahydropyrimidine-5-carboxylate (6c)

Yellow solid; mp 217–219 °C; molecular formula: C_28_H_28_F_3_N_5_O_4_, MW: 555.56. ^1^H NMR (400 MHz, DMSO-*d*_6_, *δ* ppm): 10.67 (1H, s, –CONH), 8.16 (1H, s, –NH), 8.02 (1H, s, –NH), 7.97 (1H, s, Ar–H), 7.90 (1H, s, Ar–H), 7.66 (1H, s, Ar–H), 7.44 (1H, s, Ar–H), 7.27–7.29 (2H, d, *J* = 8 Hz, Ar–H), 7.16–7.18 (2H, d, *J* = 8 Hz, Ar–H), 5.14 (1H, s, –CH), 3.99–4.08 (2H, q, –CH_2_), 3.59 (2H, s, –CH_2_), 3.09 (3H, s, –CH_3_), 2.50 (3H, s, –CH_3_), 2.16 (3H, s, –CH_3_), 1.12–1.15 (3H, t, –CH_3_). ^13^C NMR (100 MHz, DMSO-*d*_6_, *δ* ppm): 168.06, 165.49, 158.53, 144.33, 137.77, 129.87, 129.31, 127.02, 126.32, 121.46, 115.70, 56.04, 40.07, 34.47, 11.06. IR (ATR): *ν̃* = 3452, 3312, 3062, 2982, 2925, 1703, 1665, 1601, 1498, 1335, 1165, 1098, 1007, 846, 708 cm^−1^. MS (ESI^+^): *m*/*z* 556.69 [M + H]^+^.

#### Ethyl 4-(4-(2-((4-((6-carbamoyl-7-methoxyquinolin-4-yl)oxy)-2-chlorophenyl)amino)-2-oxoethyl)phenyl)-6-methyl-2-oxo-1,2,3,4-tetrahydropyrimidine-5-carboxylate (8a)

White solid; mp 201–203 °C; molecular formula: C_33_H_30_ClN_5_O_7_, MW: 644.08. ^1^H NMR (400 MHz, DMSO-*d*_6_, *δ* ppm): 10.30 (1H, s, –CONH), 9.18 (1H, s, –NH), 8.45 (1H, s, –NH), 7.71–7.73 (4H, m, Ar–H), 7.50 (1H, s, Ar–H), 7.28–7.30 (1H, d, *J* = 8 Hz, Ar–H), 7.22 (2H, s, –NH_2_), 7.19–7.21 (4H, m, Ar–H), 6.42 (1H, s, Ar–H), 5.13 (1H, s, –CH), 4.00–4.04 (2H, q, –CH_2_), 3.98 (3H, s, –OCH_3_), 3.92 (2H, s, –CH_2_), 2.24 (3H, s, –CH_3_), 1.10–1.13 (3H, t, –CH_3_). ^13^C NMR (100 MHz, DMSO-*d*_6_, *δ* ppm): 168.06, 165.49, 158.53, 156.43, 144.33, 132.39, 131.29, 129.87, 127.02, 126.32, 121.99, 115.70, 114.58, 56.04, 40.07, 34.47, 11.06. IR (ATR): *ν̃* = 3450, 3365, 3310, 3063, 2984, 2924, 1700, 1665, 1600, 1498, 1345, 1240, 1090, 1007, 843, 700 cm^−1^. MS (ESI^+^): *m*/*z* 645.76 [M + H]^+^.

#### Ethyl 4-(4-(2-((4-((6-carbamoyl-7-methoxyquinolin-4-yl)oxy)-2-chlorophenyl)amino)-2-oxoethyl)phenyl)-6-methyl-2-thioxo-1,2,3,4-tetrahydropyrimidine-5-carboxylate (8b)

White solid; mp 194–196 °C; molecular formula: C_33_H_30_ClN_5_O_6_S, MW: 660.14. ^1^H NMR (400 MHz, DMSO-*d*_6_, *δ* ppm): 10.30 (1H, s, –CONH), 8.45 (1H, s, –NH), 7.96 (1H, s, –NH), 7.71–7.73 (2H, d, *J* = 8 Hz, Ar–H), 7.50 (2H, s, Ar–H), 7.38 (2H, s, Ar–H), 7.28 (2H, s, –NH_2_), 7.20–7.22 (4H, m, Ar–H), 7.17 (1H, s, Ar–H), 5.16 (1H, s, –CH), 4.02–4.08 (2H, q, –CH_2_), 3.98 (3H, s, –OCH_3_), 3.74 (2H, s, –CH_2_), 2.48 (3H, s, –CH_3_), 1.12–1.16 (3H, t, –CH_3_). ^13^C NMR (100 MHz, DMSO-*d*_6_, *δ* ppm): 168.06, 165.49, 156.43, 144.33, 135.27, 131.29, 129.37, 124.99, 114.58, 40.07, 34.47, 11.06. IR (ATR): *ν̃* = 3450, 3365, 3310, 3059, 2981, 2921, 1703, 1660, 1597, 1495, 1200, 1098, 1007, 841, 700 cm^−1^. MS (ESI^−^): *m*/*z* 661.25 [M − H]^+^.

#### Ethyl 4-(4-(2-((4-((6-carbamoyl-7-methoxyquinolin-4-yl)oxy)-2-chlorophenyl)amino)-2-oxoethyl)phenyl)-1,6-dimethyl-2-oxo-1,2,3,4-tetrahydropyrimidine-5-carboxylate (8c)

White solid; mp 187–189 °C; molecular formula: C_34_H_32_ClN_5_O_7_, MW: 658.11. ^1^H NMR (400 MHz, DMSO-*d*_6_, *δ* ppm): 10.45 (1H, s, –CONH), 8.50 (1H, s, –NH), 7.66–7.68 (2H, d, *J* = 8 Hz, Ar–H), 7.50 (2H, s, Ar–H), 7.48 (2H, s, Ar–H), 7.44 (2H, s, –NH_2_), 7.36–7.40 (4H, m, Ar–H), 7.34 (1H, s, Ar–H), 5.20 (1H, s, –CH), 4.00–4.04 (2H, q, –CH_2_), 4.02 (3H, s, –OCH_3_), 3.88 (3H, s, –CH_3_), 3.76 (2H, s, –CH_2_), 2.22 (3H, s, –CH_3_), 1.09–1.14 (3H, t, –CH_3_). ^13^C NMR (100 MHz, DMSO-*d*_6_, *δ* ppm): 168.06, 165.49, 156.43, 144.33, 132.61, 132.39, 131.57, 126.32, 121.99, 114.58, 56.04, 40.07, 34.47, 11.06. IR (ATR): *ν̃* = 3449, 3363, 3311, 3062, 2982, 2925, 1701, 1662, 1600, 1498, 1345, 1240, 1090, 1007, 845, 700 cm^−1^. MS (ESI^−^): *m*/*z* 658.33 [M − H]^−^.

#### Ethyl 4-(4-(2-((4-((3-chloro-4-fluorophenyl)amino)-7-(((*S*)-tetrahydrofuran-3-yl)oxy)quinazolin-6-yl)amino)-2-oxoethyl)phenyl)-6-methyl-2-oxo-1,2,3,4-tetrahydropyrimidine-5-carboxylate (10a)

Brown solid; mp 188–190 °C; molecular formula: C_34_H_32_ClFN_6_O_6_, MW: 675.11. ^1^H NMR (400 MHz, DMSO-*d*_6_, *δ* ppm): 9.78 (1H, s, –CONH), 9.40 (1H, s, –NH), 9.19 (1H, s, –NH), 8.77 (1H, s, Ar–H), 8.20 (1H, s, Ar–H), 7.78 (1H, s, Ar–H), 7.72 (2H, s, Ar–H), 7.34–7.38 (3H, m, Ar–H), 7.18–7.21 (3H, s, Ar–H), 5.12 (1H, s, –CH), 3.91 (6H, m, –CH_2_), 3.86–3.90 (2H, q, –CH_2_), 3.80 (1H, s, –CH), 2.34 (2H, s, –CH_2_), 2.30 (3H, s, –CH_3_), 1.08–1.12 (3H, t, –CH_3_). ^13^C NMR (100 MHz, DMSO-*d*_6_, *δ* ppm): 168.06, 165.49, 144.33, 137.77, 136.44, 132.61, 132.20, 130.20, 129.31, 126.32, 123.72, 123.13, 121.46, 40.07, 34.47, 11.06. IR (ATR): *ν̃* = 3451, 3315, 3062, 2982, 2924, 1700, 1663, 1601, 1497, 1340, 1245, 1090, 1007, 843, 700 cm^−1^. MS (ESI^+^): *m*/*z* 675.30 [M + H]^+^.

#### Ethyl 4-(4-(2-((4-((3-chloro-4-fluorophenyl)amino)-7-(((*S*)-tetrahydrofuran-3-yl)oxy)quinazolin-6-yl)amino)-2-oxoethyl)phenyl)-6-methyl-2-thioxo-1,2,3,4-tetrahydropyrimidine-5-carboxylate (10b)

Brown solid; mp 202–204 °C; molecular formula: C_34_H_32_ClFN_6_O_5_S, MW: 691.18. ^1^H NMR (400 MHz, DMSO-*d*_6_, *δ* ppm): 10.31 (1H, s, –CONH), 10.09 (1H, s, –NH), 9.63 (1H, s, –NH), 9.25 (1H, s, Ar–H), 8.90 (1H, s, Ar–H), 8.71 (1H, s, Ar–H), 8.50 (1H, s, Ar–H), 8.46 (1H, s, Ar–H), 8.43 (1H, s, Ar–H), 7.92 (2H, s, Ar–H), 7.44 (1H, s, Ar–H), 7.27–7.31 (3H, m, Ar–H), 7.12–7.17 (3H, m, Ar–H), 5.15 (1H, s, –CH), 3.97–4.02 (2H, q, –CH_2_), 3.60 (2H, s, –CH_2_), 2.28 (3H, s, –CH_3_), 2.18 (3H, s, –CH_3_), 1.08–1.12 (3H, t, –CH_3_). ^13^C NMR (100 MHz, DMSO-*d*_6_, *δ* ppm): 168.06, 165.19, 144.33, 136.53, 136.44, 132.19, 131.57, 130.20, 129.24, 128.63, 126.32, 124.73, 122.34, 122.21, 40.07, 34.47, 11.06. IR (ATR): *ν̃* = 3452, 3310, 3062, 2981, 2924, 1702, 1660, 1598, 1496, 1200, 1340, 1090, 1007, 843, 700 cm^−1^. MS (ESI^−^): *m*/*z* 692.40 [M − H]^+^.

#### Ethyl 4-(4-(2-((4-((3-chloro-4-fluorophenyl)amino)-7-(((*S*)-tetrahydrofuran-3-yl)oxy)quinazolin-6-yl)amino)-2-oxoethyl)phenyl)-1,6-dimethyl-2-oxo-1,2,3,4-tetrahydropyrimidine-5-carboxylate (10c)

Brown solid; mp 181–183 °C; molecular formula: C_35_H_34_ClFN_6_O_6_, MW: 689.14. ^1^H NMR (400 MHz, DMSO-*d*_6_, *δ* ppm): 10.06 (1H, s, –CONH), 9.34 (1H, s, –NH), 9.10 (1H, s, –NH), 8.25 (1H, s, Ar–H), 7.66 (1H, s, Ar–H), 7.44 (2H, s, Ar–H), 7.31–7.34 (3H, m, Ar–H), 7.22–7.25 (3H, s, Ar–H), 5.18 (1H, s, –CH), 3.97–4.02 (2H, q, –CH_2_), 3.94 (1H, s, –CH_2_), 3.90 (6H, m, –CH_2_), 3.81 (2H, s, –CH_2_), 2.38 (3H, s, CH_3_), 2.32 (3H, s, CH_3_), 1.10–1.12 (3H, t, –CH_3_). ^13^C NMR (100 MHz, DMSO-*d*_6_, *δ* ppm): 168.06, 154.36, 149.41, 139.65, 139.63, 137.39, 136.83, 133.55, 130.73, 129.24, 125.53, 122.80, 122.21, 122.10, 116.64, 40.07, 32.93, 11.06. IR (ATR): *ν̃* = 3451, 3313, 3063, 2982, 2924, 1700, 1663, 1601, 1498, 1340, 1245, 1090, 1007, 843, 700 cm^−1^. MS (ESI^−^): *m*/*z* 690.40 [M − H]^+^.

#### Ethyl 6-methyl-4-(4-(2-((4-methyl-3-((4-(pyridin-3-yl)pyrimidin-2-yl)amino)phenyl)amino)-2-oxoethyl)phenyl)-2-oxo-1,2,3,4-tetrahydropyrimidine-5-carboxylate (12a)

Grey solid; mp 198–200 °C; molecular formula: C_32_H_31_N_7_O_4_, MW: 577.65. ^1^H NMR (400 MHz, DMSO-*d*_6_, *δ* ppm): 10.09 (1H, s, –CONH), 9.25 (1H, s, –NH), 8.90 (1H, s, Ar–H), 8.68–8.70 (1H, d, *J* = 8 Hz, Ar–H), 8.49–8.51 (1H, d, *J* = 8 Hz, Ar–H), 8.43–8.45 (1H, d, *J* = 8 Hz, Ar–H), 8.17 (1H, s, –NH), 7.92 (1H, s, Ar–H), 7.71 (1H, s, Ar–H), 7.41–7.47 (2H, q, Ar–H), 7.26–7.28 (3H, d, *J* = 8 Hz, Ar–H), 7.17–7.19 (2H, d, *J* = 8 Hz, Ar–H), 7.12–7.14 (1H, d, *J* = 8 Hz, Ar–H), 5.12 (1H, s, –CH), 3.94–3.98 (2H, q, –CH_2_), 3.59 (2H, s, –CH_2_), 2.23 (3H, s, –CH_3_), 2.18 (3H, s, –CH_3_), 1.07–1.11 (3H, t, –CH_3_). ^13^C NMR (100 MHz, DMSO-*d*_6_, *δ* ppm): 168.06, 165.49, 158.53, 154.36, 144.33, 134.58, 132.39, 129.87, 127.02, 125.78, 121.00, 116.64, 115.70, 56.04, 40.07, 34.47, 11.06. IR (ATR): *ν̃* = 3453, 3314, 3062, 2983, 2924, 1701, 1664, 1601, 1498, 1345, 1248, 1090, 1007, 845, 700 cm^−1^. MS (ESI^+^): *m*/*z* 578.21 [M + H]^+^.

#### Ethyl 6-methyl-4-(4-(2-((4-methyl-3-((4-(pyridin-3-yl)pyrimidin-2-yl)amino)phenyl)amino)-2-oxoethyl)phenyl)-2-thioxo-1,2,3,4-tetrahydropyrimidine-5-carboxylate (12b)

Grey solid; mp 206–208 °C; molecular formula: C_32_H_31_N_7_O_3_S, MW: 593.71. ^1^H NMR (400 MHz, DMSO-*d*_6_, *δ* ppm): 10.32 (1H, s, –CONH), 10.09 (1H, s, –NH), 9.63 (1H, s, –NH), 9.25 (1H, s, Ar–H), 8.90 (1H, s, Ar–H), 8.70 (1H, s, Ar–H), 8.50 (1H, d, *J* = 8 Hz, Ar–H), 8.43 (1H, d, *J* = 8 Hz, Ar–H), 7.91 (1H, s, Ar–H), 7.44–7.47 (2H, m, Ar–H), 7.27–7.31 (3H, m, Ar–H), 7.12–7.17 (3H, m, Ar–H), 5.14 (1H, s, –CH), 3.97–4.02 (2H, q, –CH_2_), 3.60 (2H, s, –CH_2_), 2.28 (3H, s, –CH_3_), 2.18 (3H, s, –CH_3_), 1.08–1.12 (3H, t, –CH_3_). ^13^C NMR (100 MHz, DMSO-*d*_6_, *δ* ppm): 168.06, 149.41, 149.39, 137.77, 137.39, 133.55, 129.31, 125.56, 122.34, 121.46, 121.00, 40.07, 32.93, 11.06. IR (ATR): *ν̃* = 3451, 3314, 3060, 2980, 2924, 1703, 1660, 1599, 1498, 1200, 1341, 1090, 1007, 841, 700 cm^−1^. MS (ESI^+^): *m*/*z* 594.79 [M + H]^+^.

#### Ethyl 1,6-dimethyl-4-(4-(2-((4-methyl-3-((4-(pyridin-3-yl)pyrimidin-2-yl)amino)phenyl)amino)-2-oxoethyl)phenyl)-2-oxo-1,2,3,4-tetrahydropyrimidine-5-carboxylate (12c)

Grey solid; mp 215–217 °C; molecular formula: C_33_H_33_N_7_O_4_, MW: 591.67. ^1^H NMR (400 MHz, DMSO-*d*_6_, *δ* ppm): 10.09 (1H, s, –CONH), 9.25 (1H, s, –NH), 8.91 (1H, s, –NH), 8.70 (1H, s, Ar–H), 8.69 (1H, s, Ar–H), 8.50 (1H, s, Ar–H), 8.49 (1H, s, Ar–H), 8.43 (1H, s, Ar–H), 7.47 (2H, s, Ar–H), 7.41–7.46 (3H, m, Ar–H), 7.12–7.15 (3H, m, Ar–H), 5.15 (1H, s, –CH), 3.97–4.07 (2H, q, –CH_2_), 3.59 (2H, s, –CH_2_), 3.08 (3H, s, –CH_3_), 2.49 (3H, s, –CH_3_), 2.18 (3H, s, CH_3_), 1.10–1.14 (3H, t, –CH_3_). ^13^C NMR (100 MHz, DMSO-*d*_6_, *δ* ppm): 168.06, 149.41, 139.65, 139.63, 137.39, 136.83, 133.55, 130.73, 129.24, 125.53, 122.21, 122.10, 40.07, 32.93, 11.06. IR (ATR): *ν̃* = 3452, 3311, 3062, 2982, 2924, 1701, 1663, 1601, 1498, 1345, 1248, 1090, 1007, 845, 700 cm^−1^. MS (ESI^+^): *m*/*z* 592.77 [M + H]^+^.

### Molecular docking studies

Molecular docking studies were performed using Schrödinger Suite against Epidermal Growth Factor Receptor (PDB ID 1M17), retrieved from the Protein Data Bank. Protein preparation was carried out using the Protein Preparation Wizard, involving hydrogen addition, protonation state adjustment at pH 7.4 ± 2.0, removal of water molecules beyond 5 Å, and energy minimization with the OPLS3 force field (RMSD = 0.20 Å). Receptor grids were generated using Glide with inner (20 × 20 × 20 Å) and outer (36 × 36 × 36 Å) box dimensions. Ligands were prepared using LigPrep at pH 7.2 ± 2.0 with OPLS3 geometry optimization. Docking was performed in Standard Precision followed by Extra Precision mode, retaining up to 10 poses per ligand, with the best pose selected based on GlideScore. Binding interactions were visualized and analyzed using BIOVIA Discovery Studio Visualizer, and results are summarized in Table 1.^18^

## Conclusion

In conclusion, twelve novel dihydropyrimidine–amide hybrid conjugates (6a–c, 8a–c, 10a–c, and 12a–c) were successfully synthesized through a convergent Biginelli reaction followed by EDC·HCl/HOBt-mediated amide coupling, enabling systematic variation of the DHPM core and appended pharmacophoric fragments. Among the synthesized compounds, 10b exhibited the most promising cytotoxicity against A549 NSCLC cells (IC_50_ = 23.31 µM; 16.11 ± 0.11 µg mL^−1^), exceeding the molar potency of methotrexate (33.01 µM). SAR analysis indicated that C2-thioxo substitution enhanced activity in series 10, whereas *N*-methylation favored activity in series 6.

Computational investigations further supported the potential of 10b, which showed a favorable EGFR docking score (−7.819 kcal mol^−1^), key hydrogen-bonding and hydrophobic interactions, and a favorable electronic profile from DFT calculations (Δ*E* = 4.045 eV; *ω* = 3.155 eV). Normal mode analysis indicated a conformationally stable ligand–EGFR interactions. However, the weak and statistically nonsignificant correlation between docking scores and cytotoxicity indicates that EGFR involvement remains a plausible hypothesis rather than a confirmed mechanism. Overall, 10b represents a promising lead for further optimization. Several limitations of the present study should be acknowledged explicitly. Compound identity rests on IR, ^1^H/^13^C NMR and ESI-MS data, and no numerical purity figure is claimed. Docking was performed only against the inactive-conformation structure 1M17, so the binding mode should be regarded as a chemotype-compatibility hypothesis rather than an established Type-I or Type-II mode; re-docking against active-conformation structures such as 2GS6 and 3POZ is planned. The DFT descriptors are gas-phase properties of the isolated molecules and are not evidence of a mechanism of cellular action. Accordingly, direct EGFR kinase assays, comparison with approved EGFR-TKIs, active-conformation docking, expanded SAR, and *in vivo* and selectivity studies are all required before therapeutic potential can be established.

## Conflicts of interest

There are no conflicts to declare.

## Abbreviations

DHPM: Dihydropyrimidine
NSCLC: Non-small cell lung cancer
VEGFR-2: Vascular endothelial growth factor receptor-2
MET: Mesenchymal–epithelial transition factor
KRAS: Kirsten rat sarcoma viral proto-oncogene
MAPK: Mitogen-activated protein kinase
NMA: Normal mode analysis

## Supplementary Material

RA-016-D6RA08838D-s001

## Data Availability

All data supporting the findings of this study, including NMR, FT-IR, mass spectra, computational DFT files, molecular docking results, and ADME analysis, are provided within the manuscript and the supplementary information (SI). Additional raw data can be made available from the corresponding author upon reasonable request. Supplementary information is available. See DOI: https://doi.org/10.1039/d6ra08838d.
