# Supplementary material for: Design, synthesis, and anticancer evaluation of Biginelli-derived DHPM–amide hybrids bearing clinically inspired kinase-directed pharmacophores: EGFR docking and DFT studies
Source: RSC Adv. 2026 Oct 19. Online ahead of print. doi: 10.1039/d6ra08838d (PMC13678441; doi:10.1039/d6ra08838d)
Supplement: Supplementary file 1 [file RA-OLF-D6RA08838D-s001.pdf]

Supplementary Information (SI) for RSC Advances.
This journal is © The Royal Society of Chemistry

Design, Synthesis, and Anticancer Evaluation of Biginelli-Derived DHPM–Amide Hybrids Bearing Clinically Inspired Kinase-Directed Pharmacophores: EGFR Docking and DFT Studies

Sagar P. Kothadiya[a], Jay B. Maheta[b], Darshna K. Lakhnotra[b], Yogesh O. Bhola[b*], Suresh B. Koradiya[a,b*]

[a]*Department of Chemistry, Atamiya University, Rajkot -360005, Gujarat, India*

[b]*Department of Chemistry, Shri M. P. Pandya Science College, Lunawada, Shri Govind Guru University, Godhra, Gujarat, India*

Corresponding author: dryobhola@gmail.com, Suresh.koradiya@gmail.com

Supporting Information

Contain
**Fig. S1:** ^{1}H NMR spectrum of final compound 6a
**Fig. S2:** ^{1}H NMR spectrum of final compound 6b
**Fig. S3:** ^{1}H NMR spectrum of final compound 6c
**Fig. S4:** ^{1}H NMR spectrum of final compound 8a
**Fig. S5:** ^{1}H NMR spectrum of final compound 8b
**Fig. S6:** ^{1}H NMR spectrum of final compound 10a
**Fig. S7:** ^{1}H NMR spectrum of final compound 10b
**Fig. S8:** ^{1}H NMR spectrum of final compound 12a
**Fig. S9:** ^{1}H NMR spectrum of final compound 12b
**Fig. S10:** ^{1}H NMR spectrum of final compound 12c
**Fig. S11:** IR spectrum of final compound 6a
**Fig. S12:** IR spectrum of final compound 6b
**Fig. S13:** IR spectrum of final compound 6c
**Fig. S14:** IR spectrum of final compound 8a
**Fig. S15:** IR spectrum of final compound 8b
**Fig. S16:** IR spectrum of final compound 8c

**Fig. S17:** IR spectrum of final compound 10a
**Fig. S18:** IR spectrum of final compound 10b
**Fig. S19:** IR spectrum of final compound 10c
**Fig. S20:** IR spectrum of final compound 12a
**Fig. S21:** IR spectrum of final compound 12b
**Fig. S22:** IR spectrum of final compound 12c
**Fig. S23:** ^{13}C NMR spectrum of final compound 6a
**Fig. S24:** ^{13}C NMR spectrum of final compound 6b
**Fig. S25:** ^{13}C NMR spectrum of final compound 6c
**Fig. S26:** ^{13}C NMR spectrum of final compound 8a
**Fig. S27:** ^{13}C NMR spectrum of final compound 8b
**Fig. S28:** ^{13}C NMR spectrum of final compound 8c
**Fig. S29:** ^{13}C NMR spectrum of final compound 10a
**Fig. S30:** ^{13}C NMR spectrum of final compound 10b
**Fig. S31:** ^{13}C NMR spectrum of final compound 10c
**Fig. S32:** ^{13}C NMR spectrum of final compound 12a
**Fig. S33:** ^{13}C NMR spectrum of final compound 12b
**Fig. S34:** ^{13}C NMR spectrum of final compound 12c
**Fig. S35:** Mass spectrum of final compound 6a
**Fig. S36**: Mass spectrum of final compound 6b
**Fig. S37:** Mass spectrum of final compound 6c
**Fig. S38**: Mass spectrum of final compound 8a
**Fig. S39**: Mass spectrum of final compound 8b
**Fig. S40**: Mass spectrum of final compound 8c
**Fig. S41**: Mass spectrum of final compound 10a
**Fig. S42**: Mass spectrum of final compound 10b
**Fig. S43**: Mass spectrum of final compound 10c
**Fig. S44**: Mass spectrum of final compound 12a
**Fig. S45**: Mass spectrum of final compound 12b
**Fig. S46**: Mass spectrum of final compound 12c
**Table S1.** In vitro cytotoxic activity (IC₅₀, μg/mL) of synthesized compounds against A549 human lung carcinoma cell line.

**Table S2.** Molecular docking results of synthesized compounds (6a-c, 8a-c, 10a-c, 12a-c) and reference inhibitors against the EGFR tyrosine kinase domain (PDB ID: 1M17).
**Fig S47:** 2D molecular docking Diagram against 1M17 Protein target
**Table S3.** Mulliken Atomic Charges of Selected Atoms in the Optimized Structure (Compound 10b)

Fig. S1: ^{1}H NMR spectrum of final compound 6a

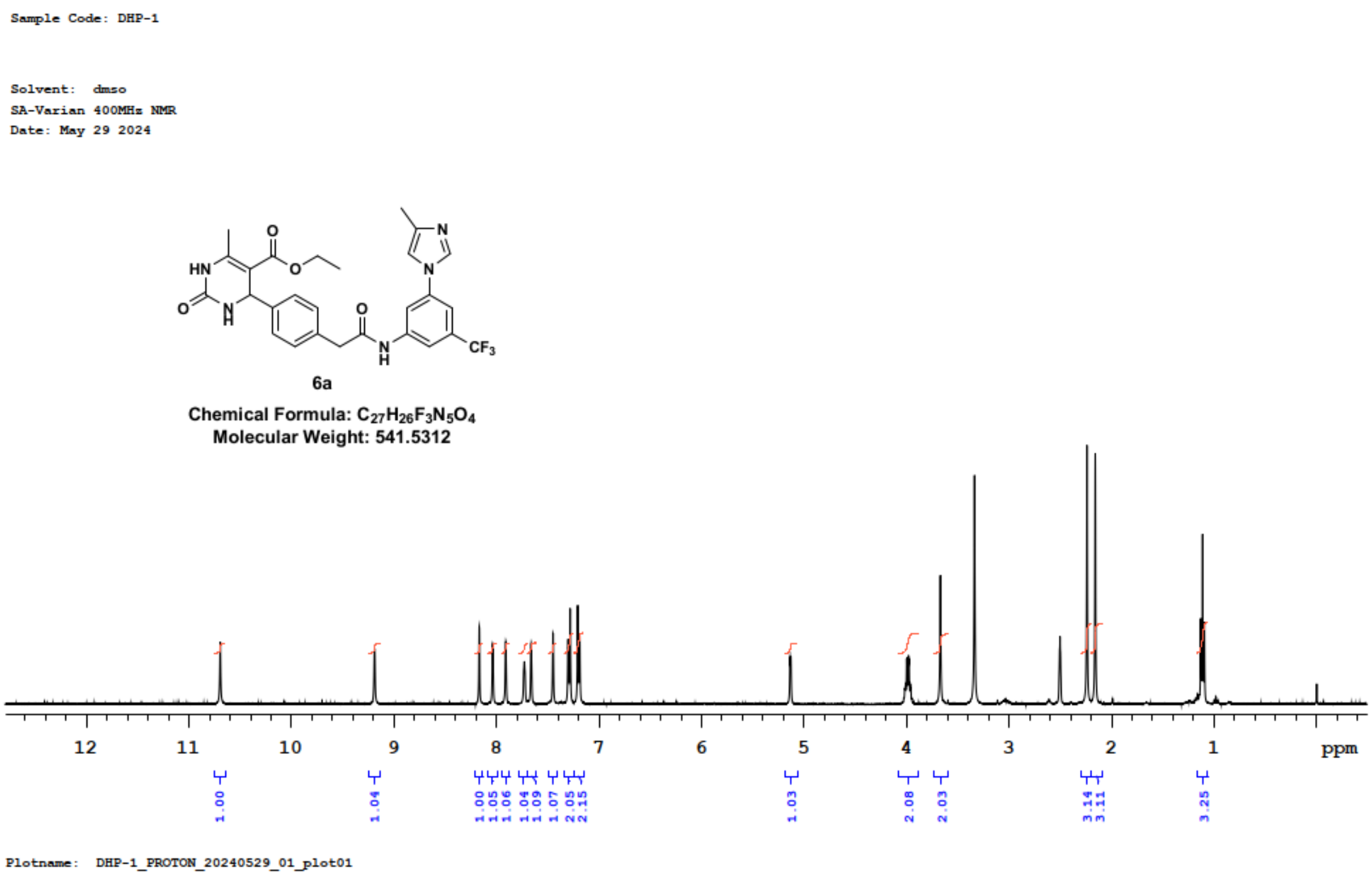

Fig. S2: ^{1}H NMR spectrum of final compound 6b

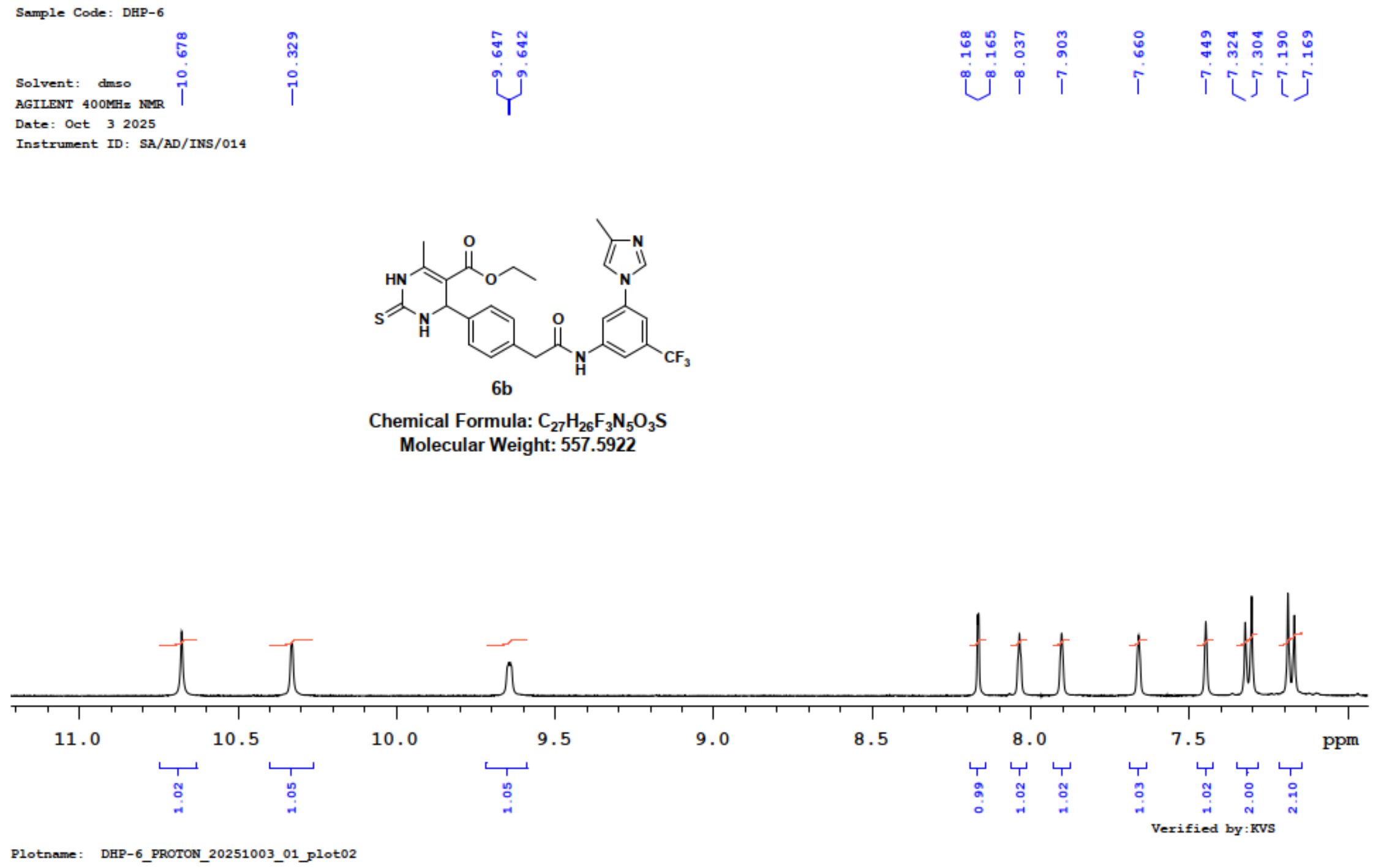

Fig. S3: ^{1}H NMR spectrum of final compound 6c

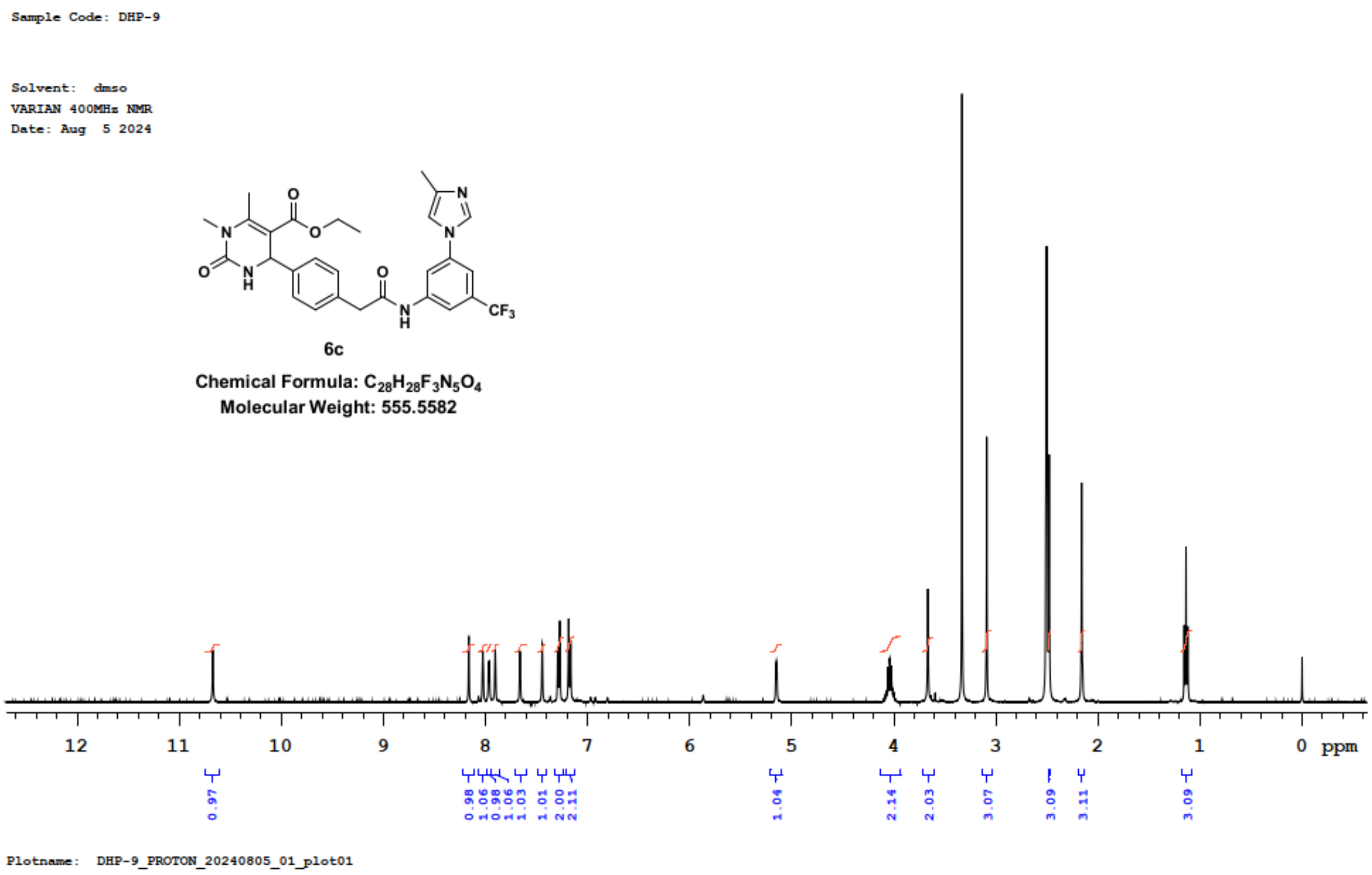

Fig. S4: ^{1}H NMR spectrum of final compound 8a

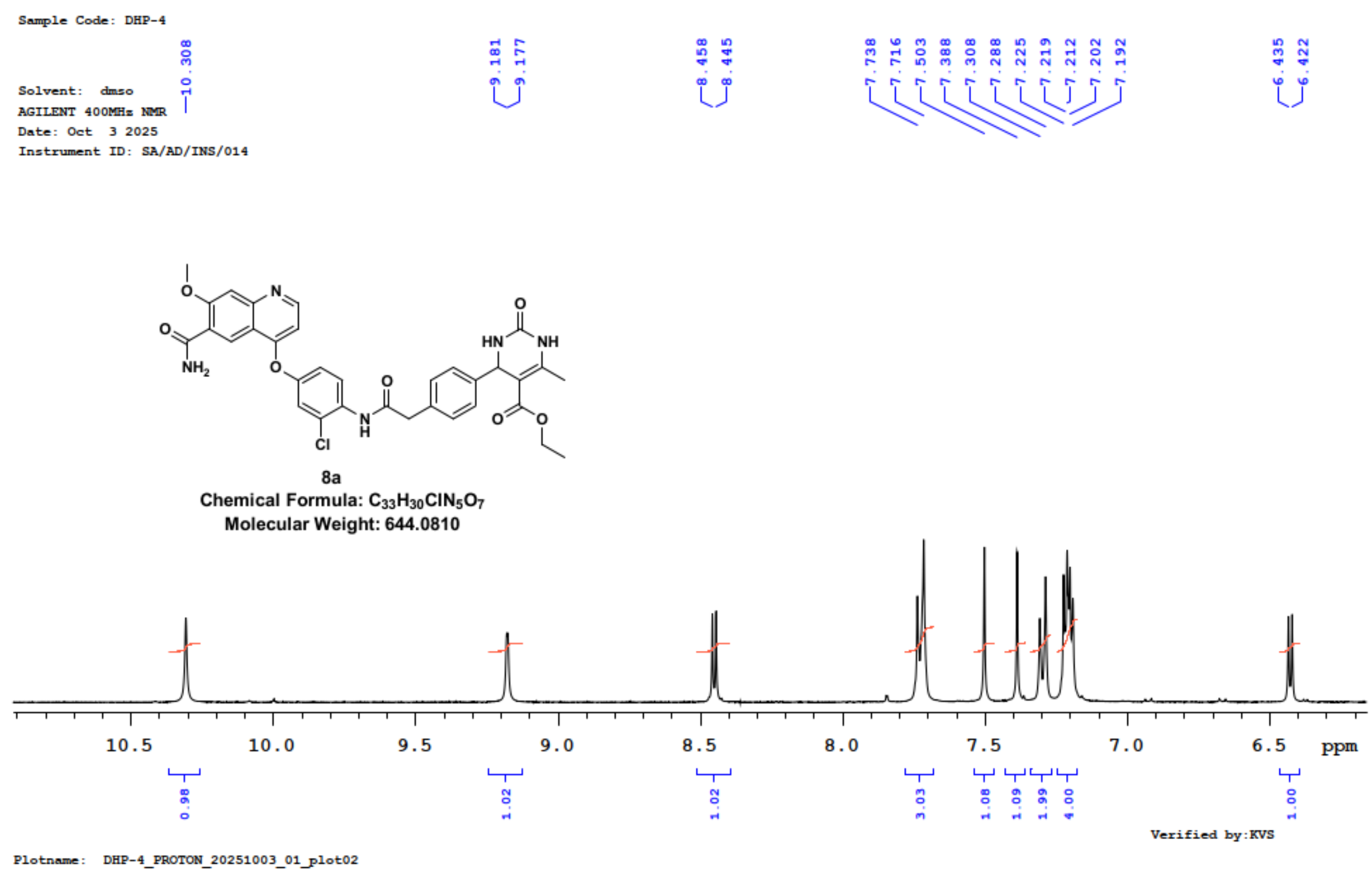

Fig. S5: ^{1}H NMR spectrum of final compound 8b

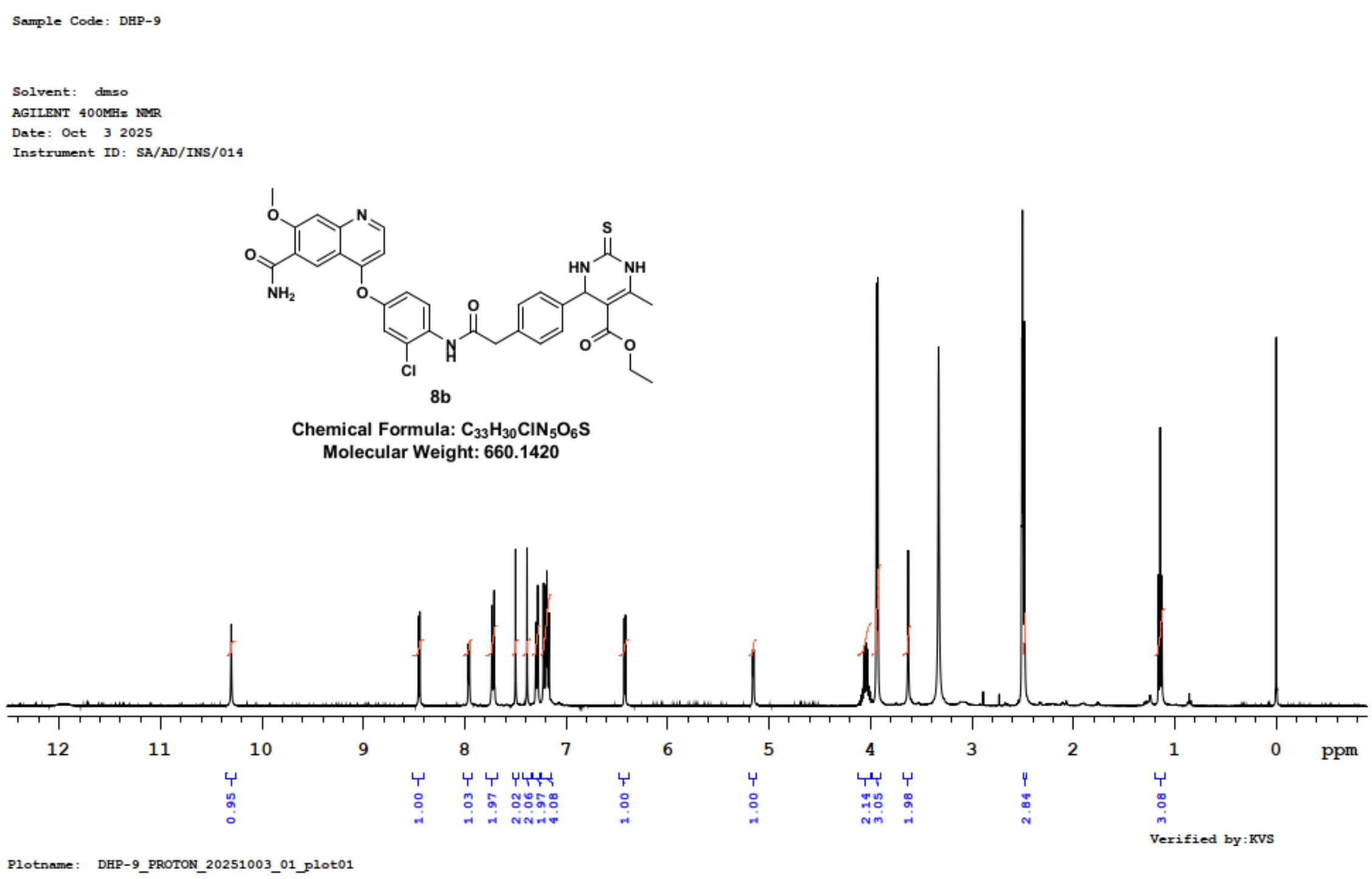

Fig. S6: ^{1}H NMR spectrum of final compound 10a

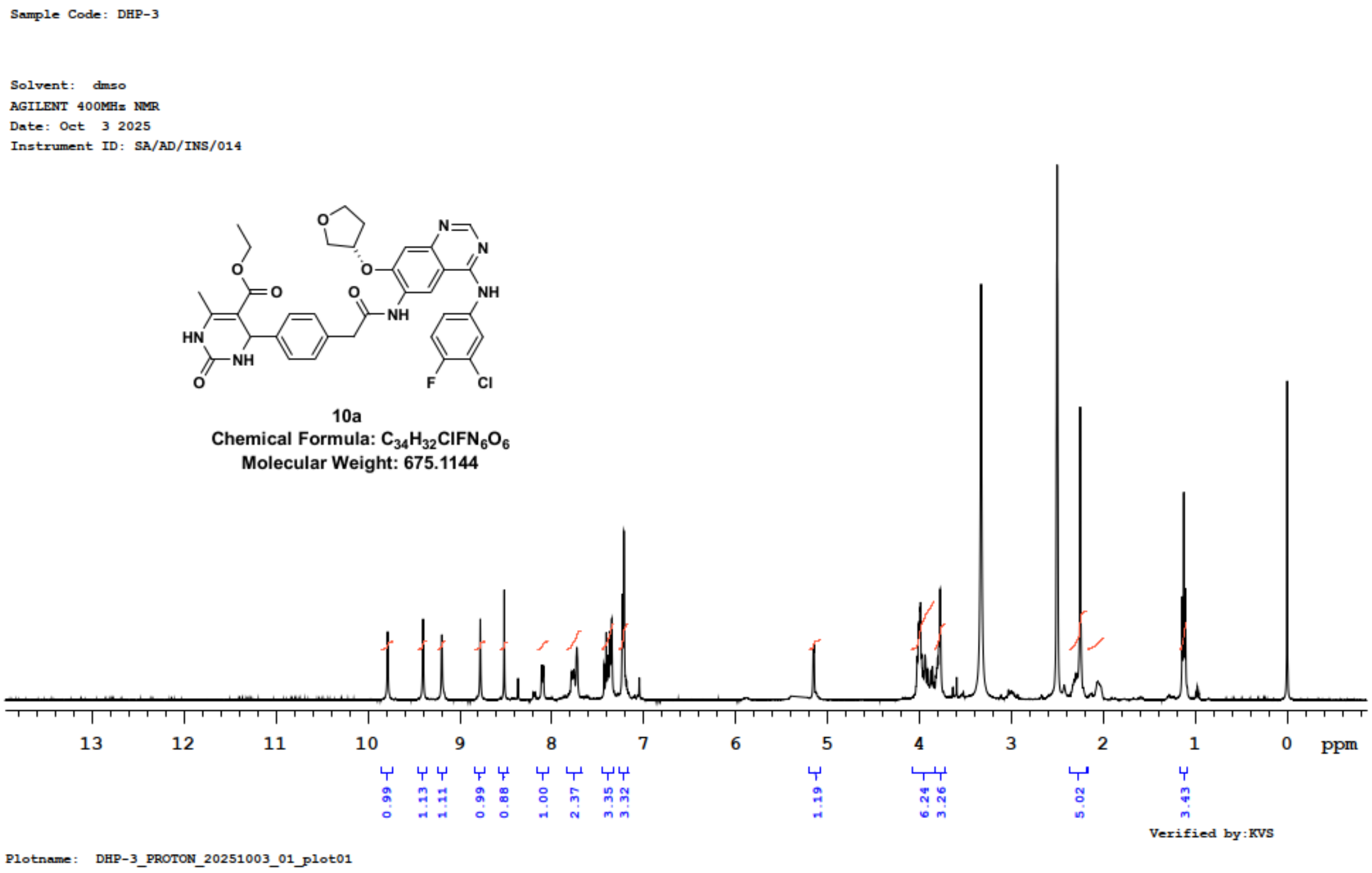

Fig. S7: ^{1}H NMR spectrum of final compound 10b

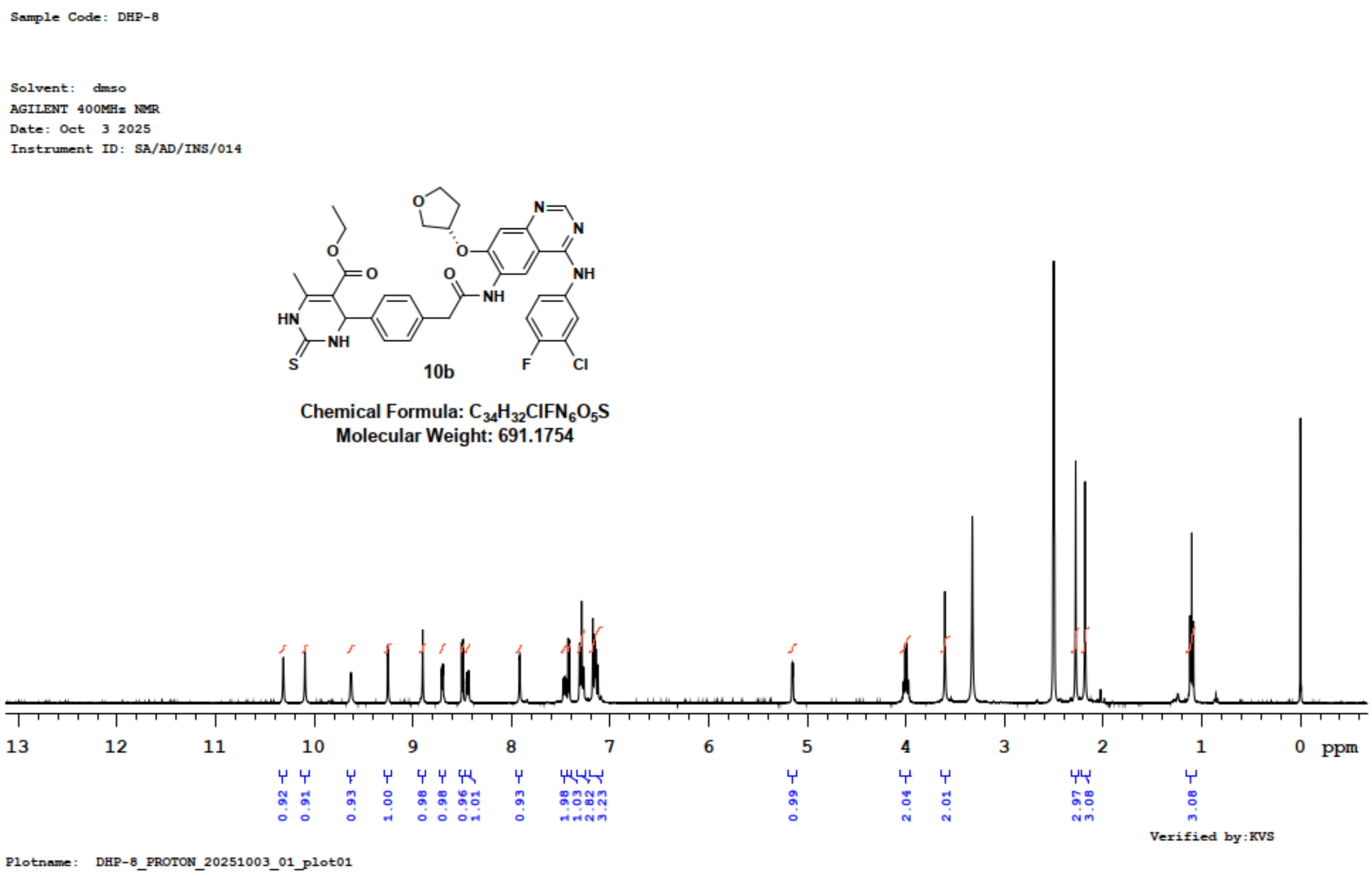

Fig. S8: ^{1}H NMR spectrum of final compound 12a

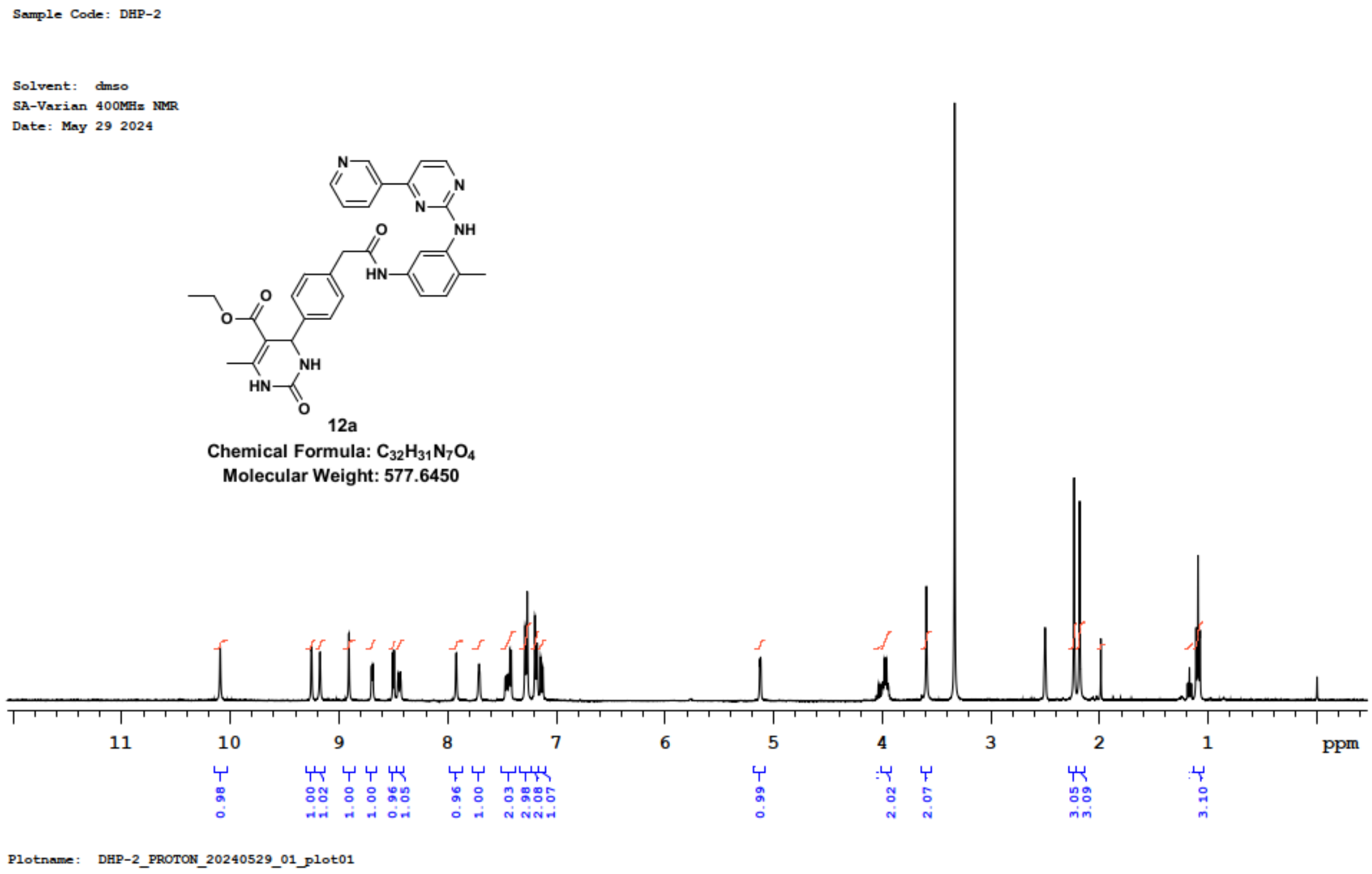

Fig. S9: ^{1}H NMR spectrum of final compound 12b

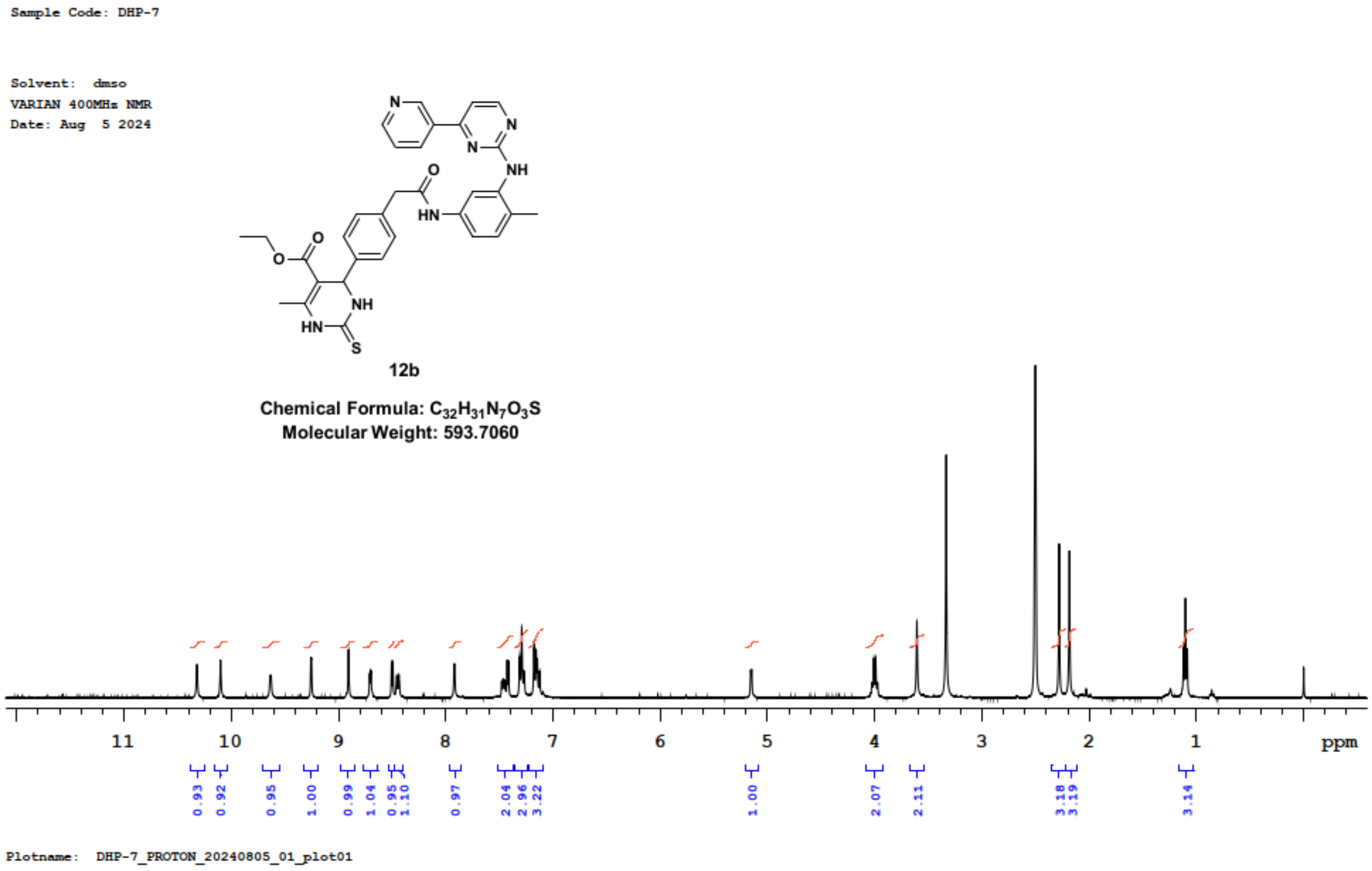

Fig. S10: ¹H NMR spectrum of final compound 12c

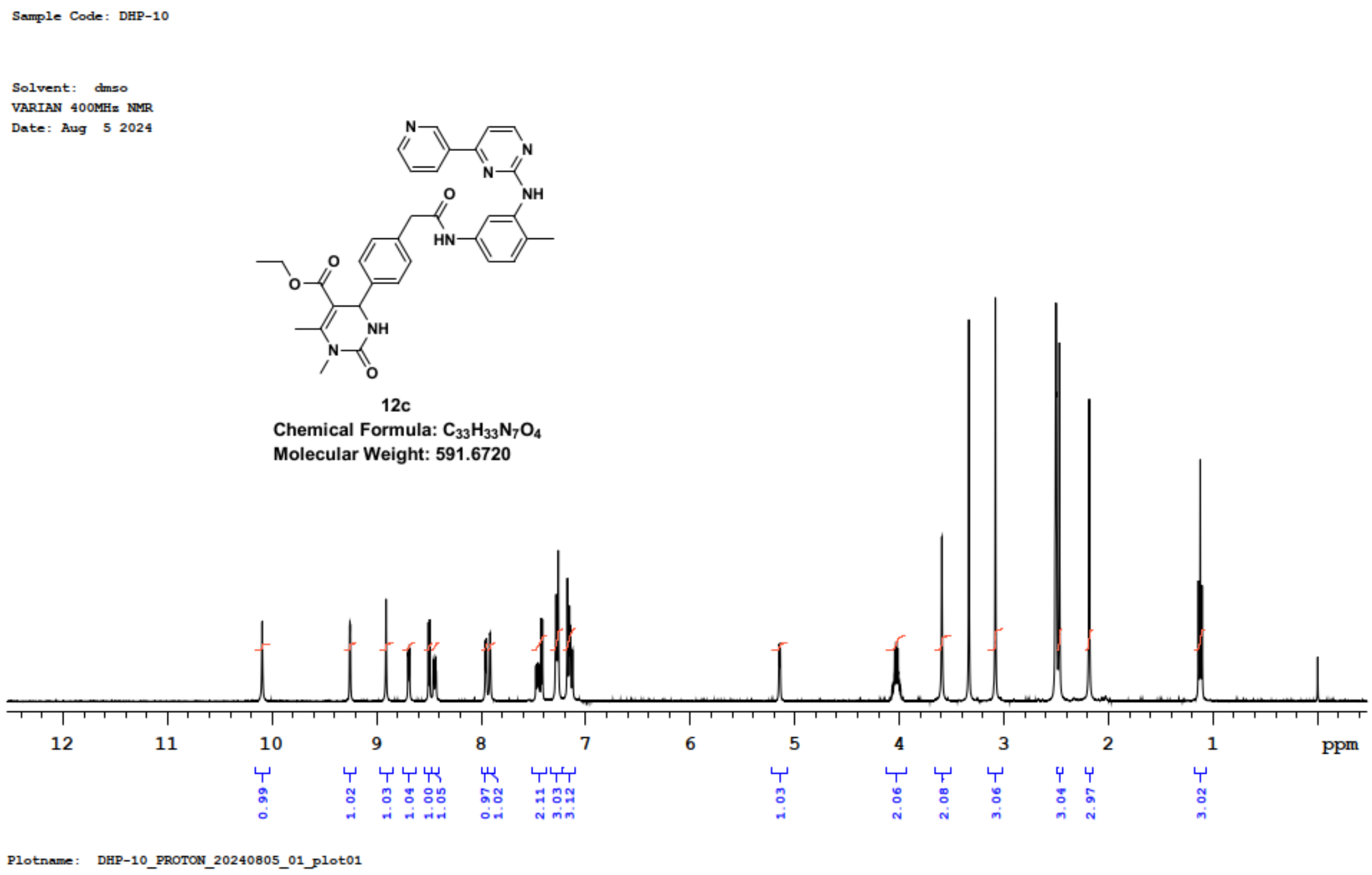

Fig. S11: IR spectrum of final compound 6a

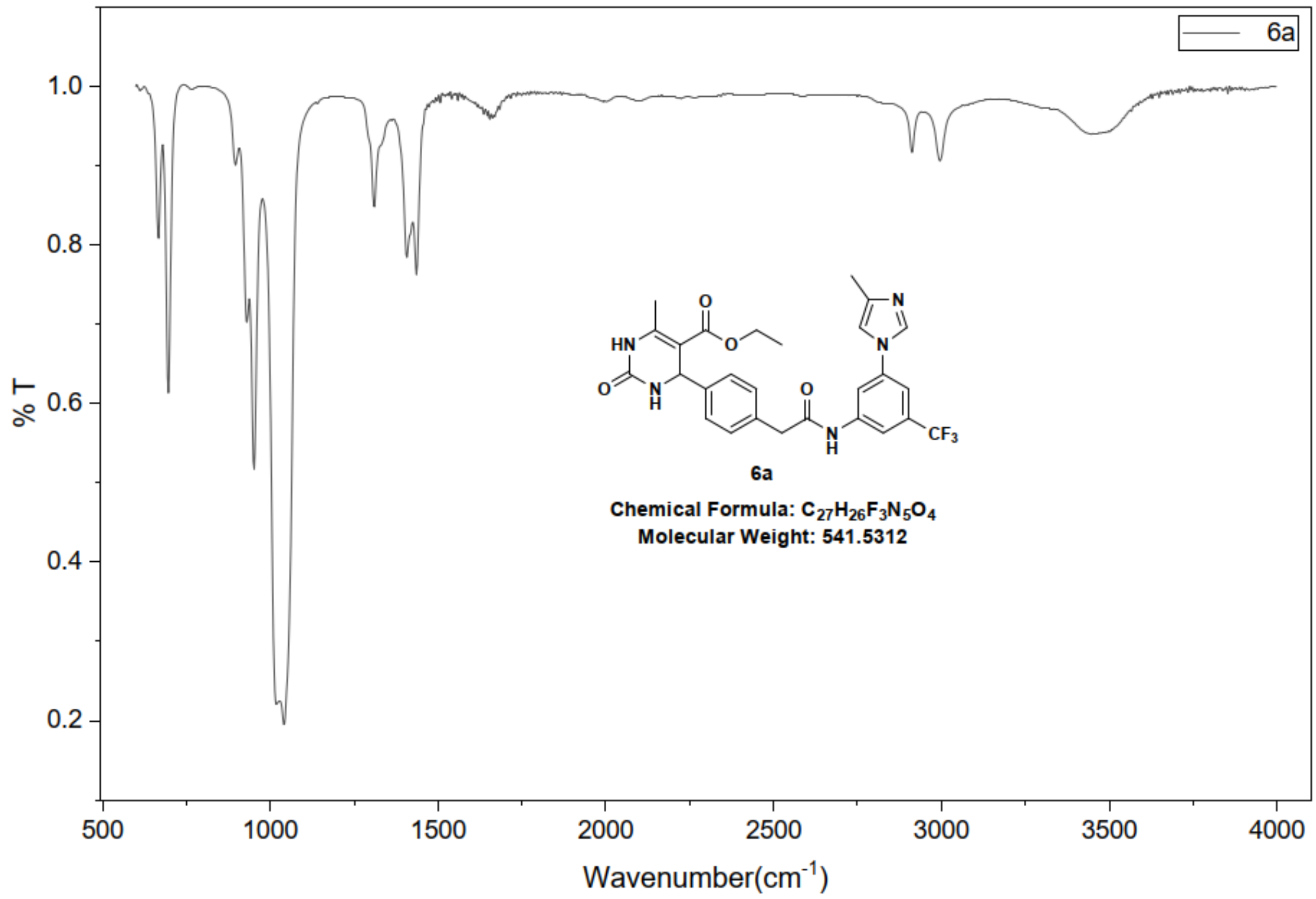

Fig. S12: IR spectrum of final compound 6b

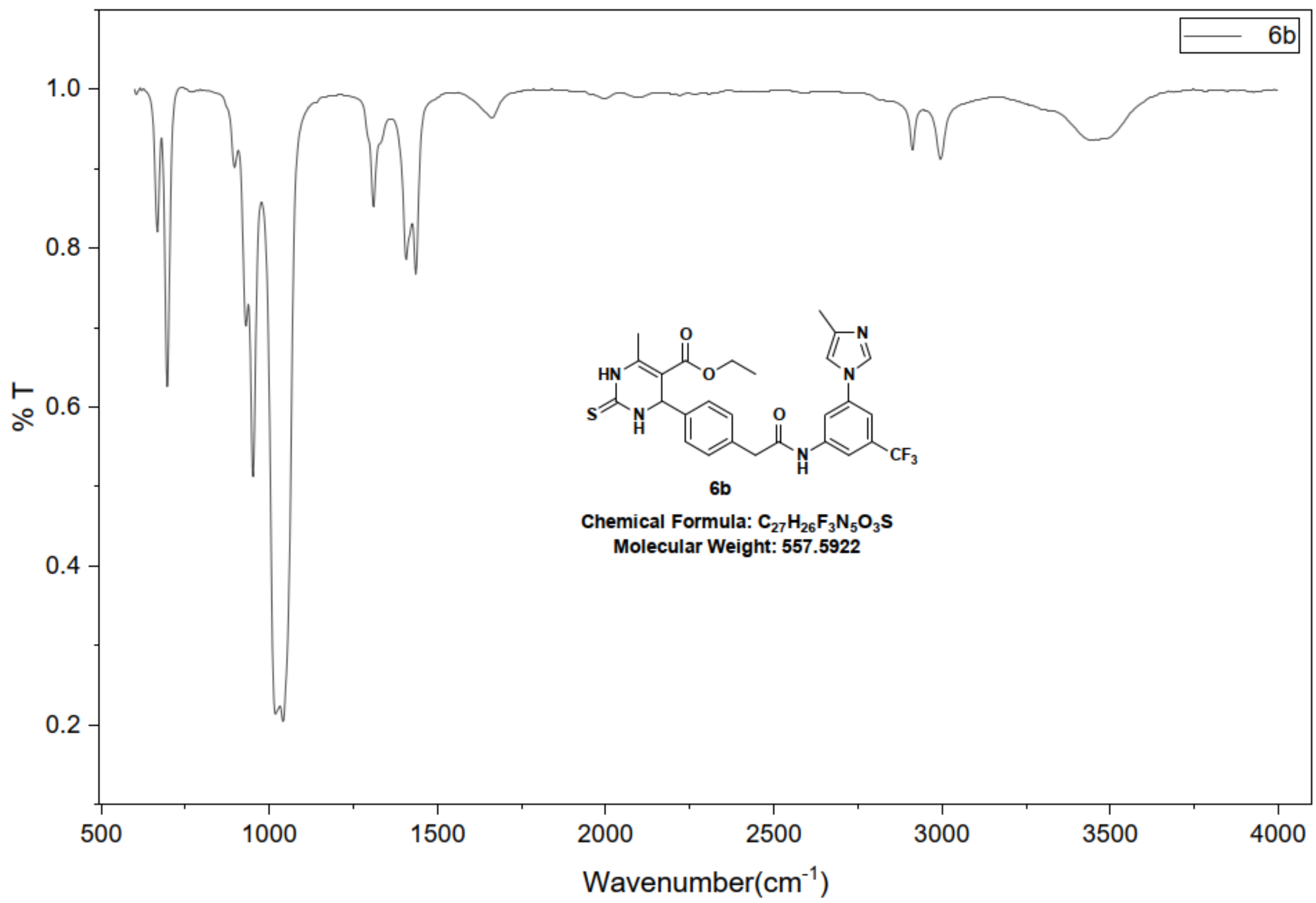

Fig. S13: IR spectrum of final compound 6c

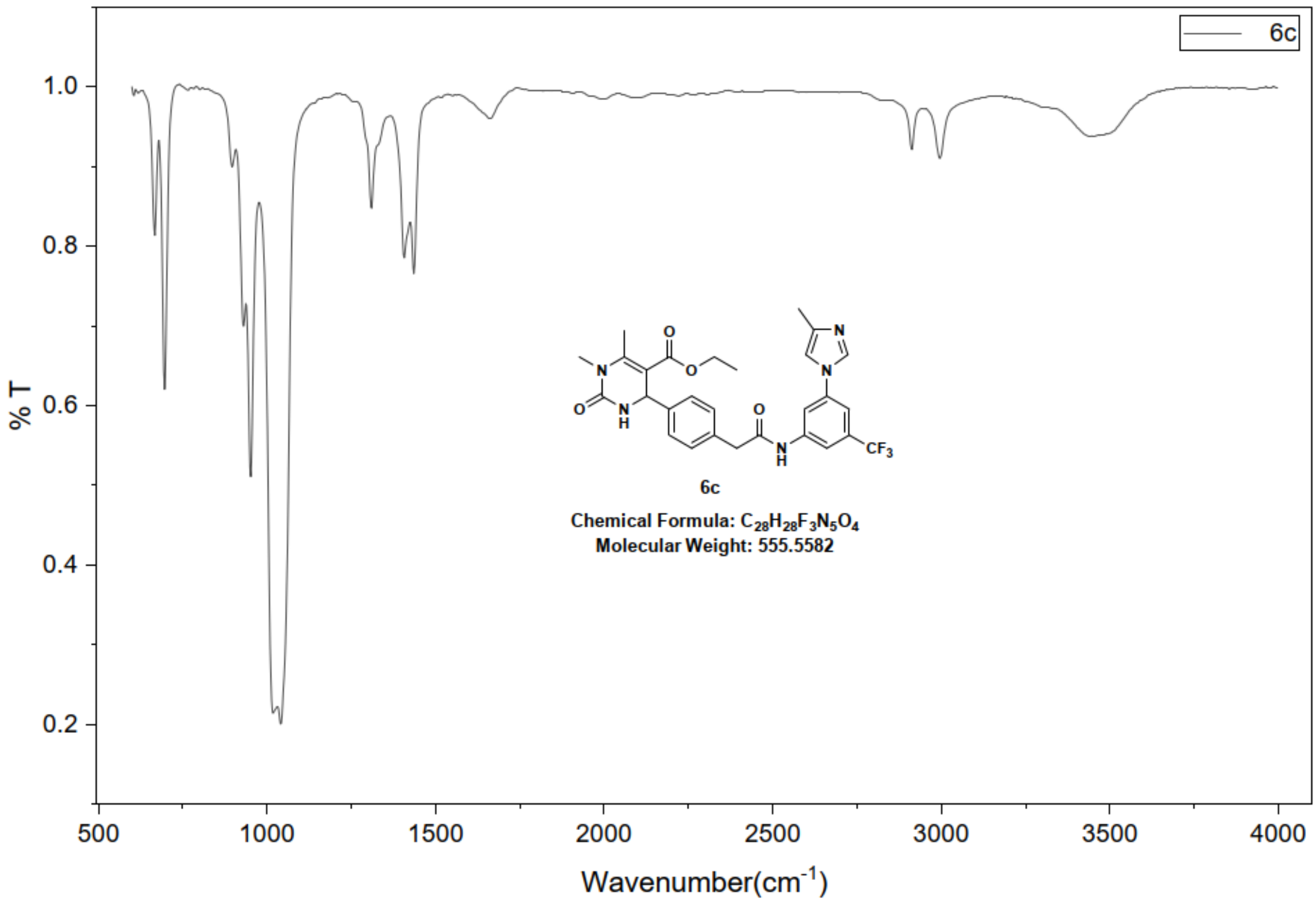

Fig. S14: IR spectrum of final compound 8a

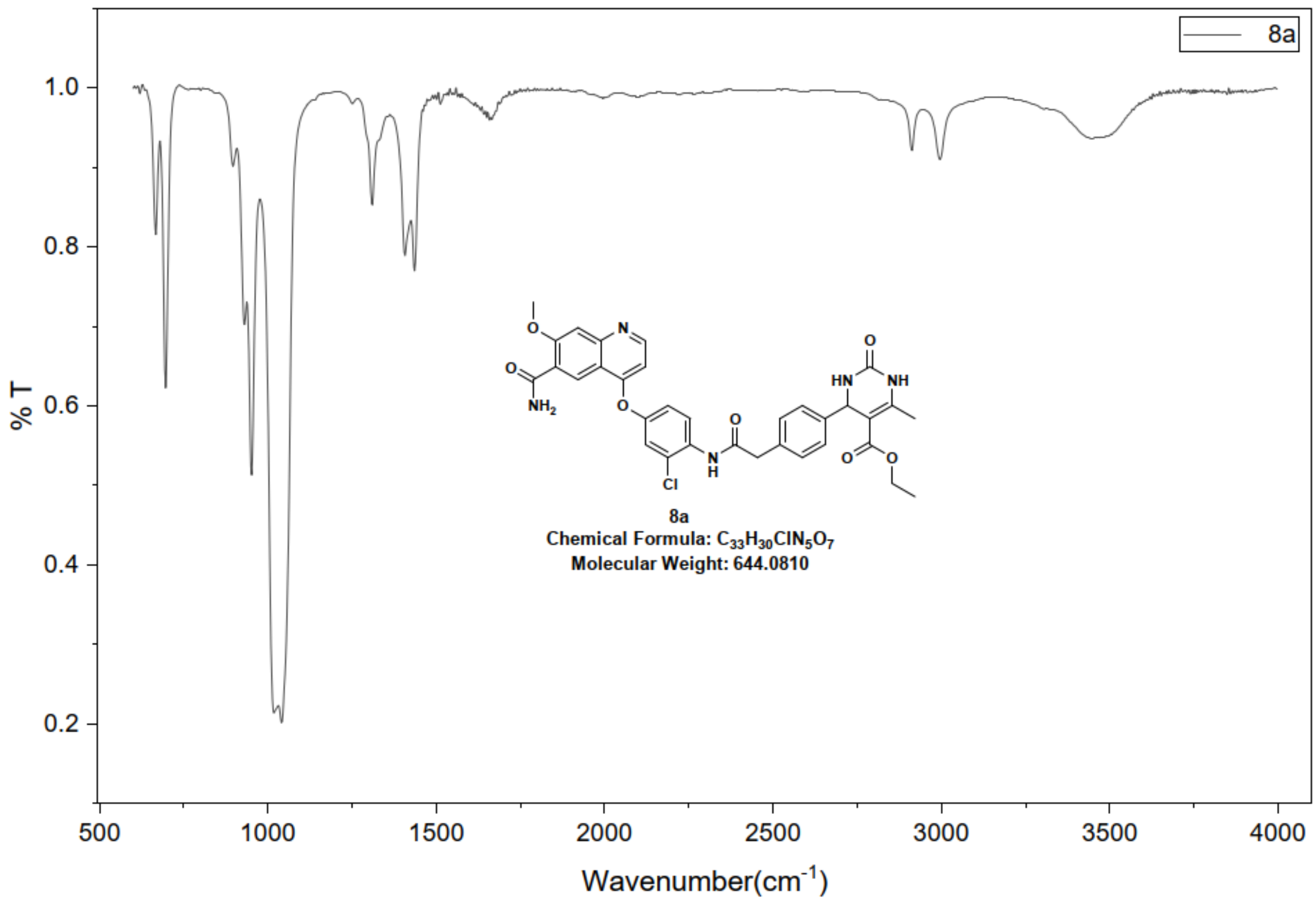

Fig. S15: IR spectrum of final compound 8b

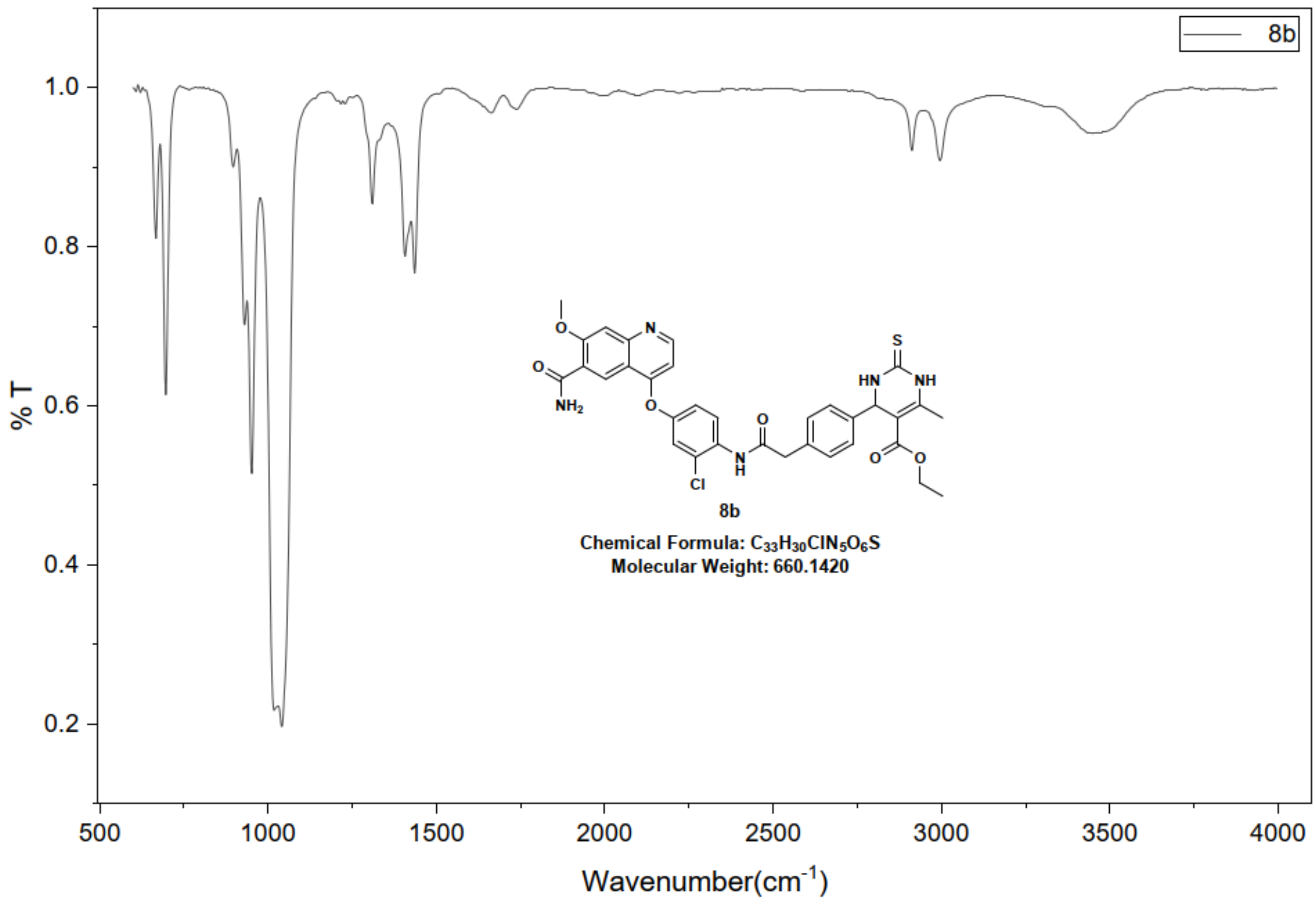

Fig. S16: IR spectrum of final compound 8c

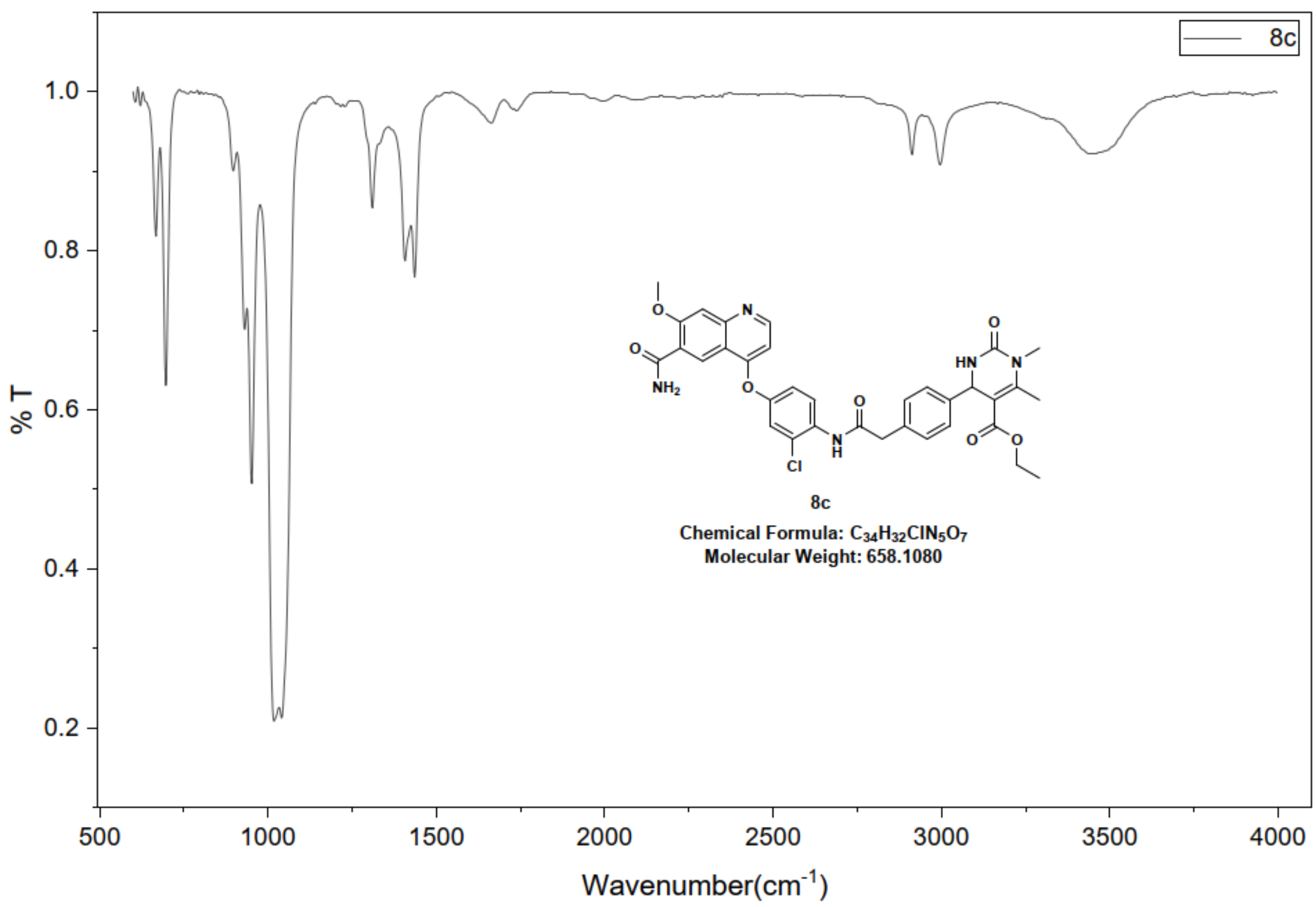

Fig. S17: IR spectrum of final compound 10a

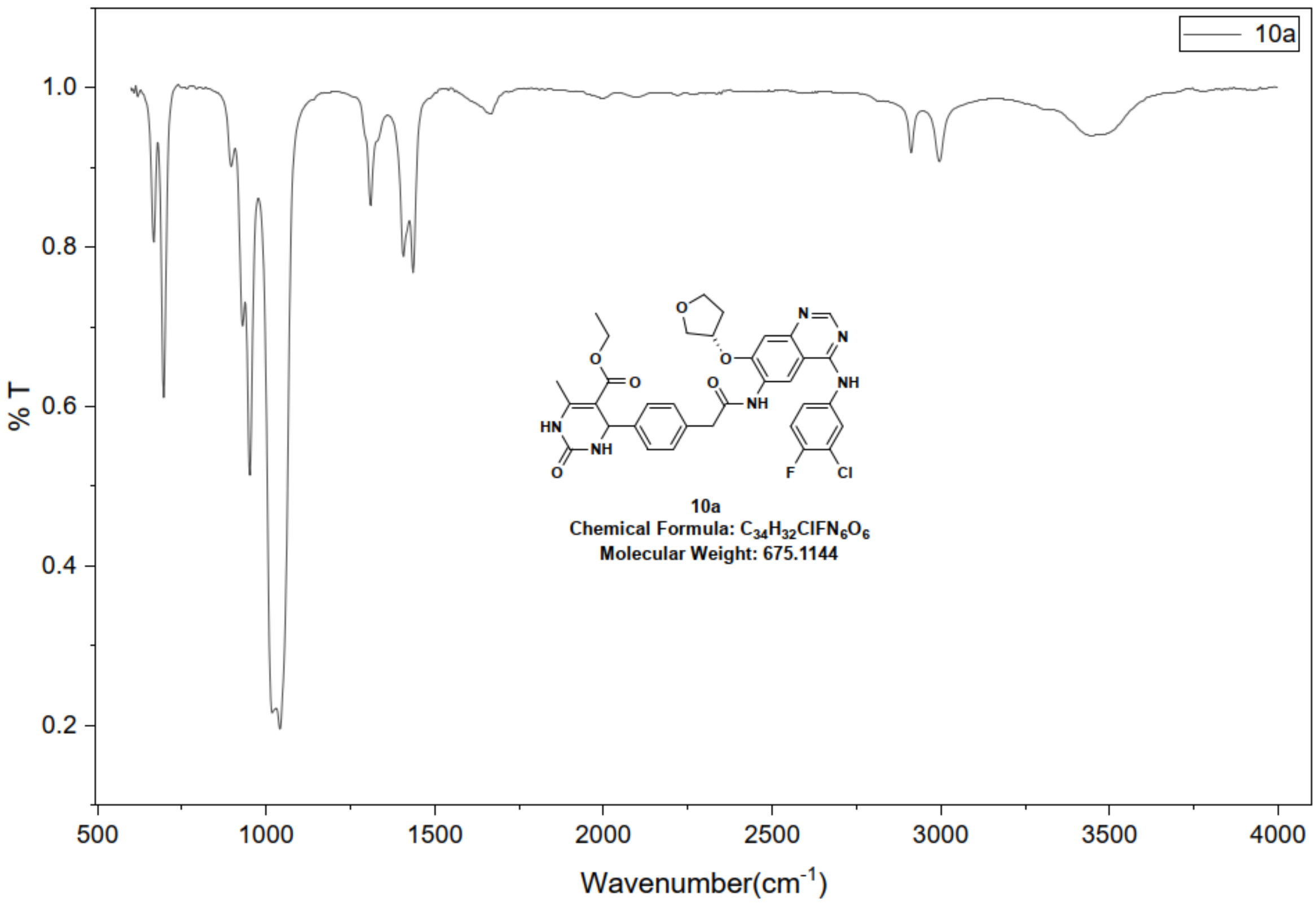

Fig. S18: IR spectrum of final compound 10b

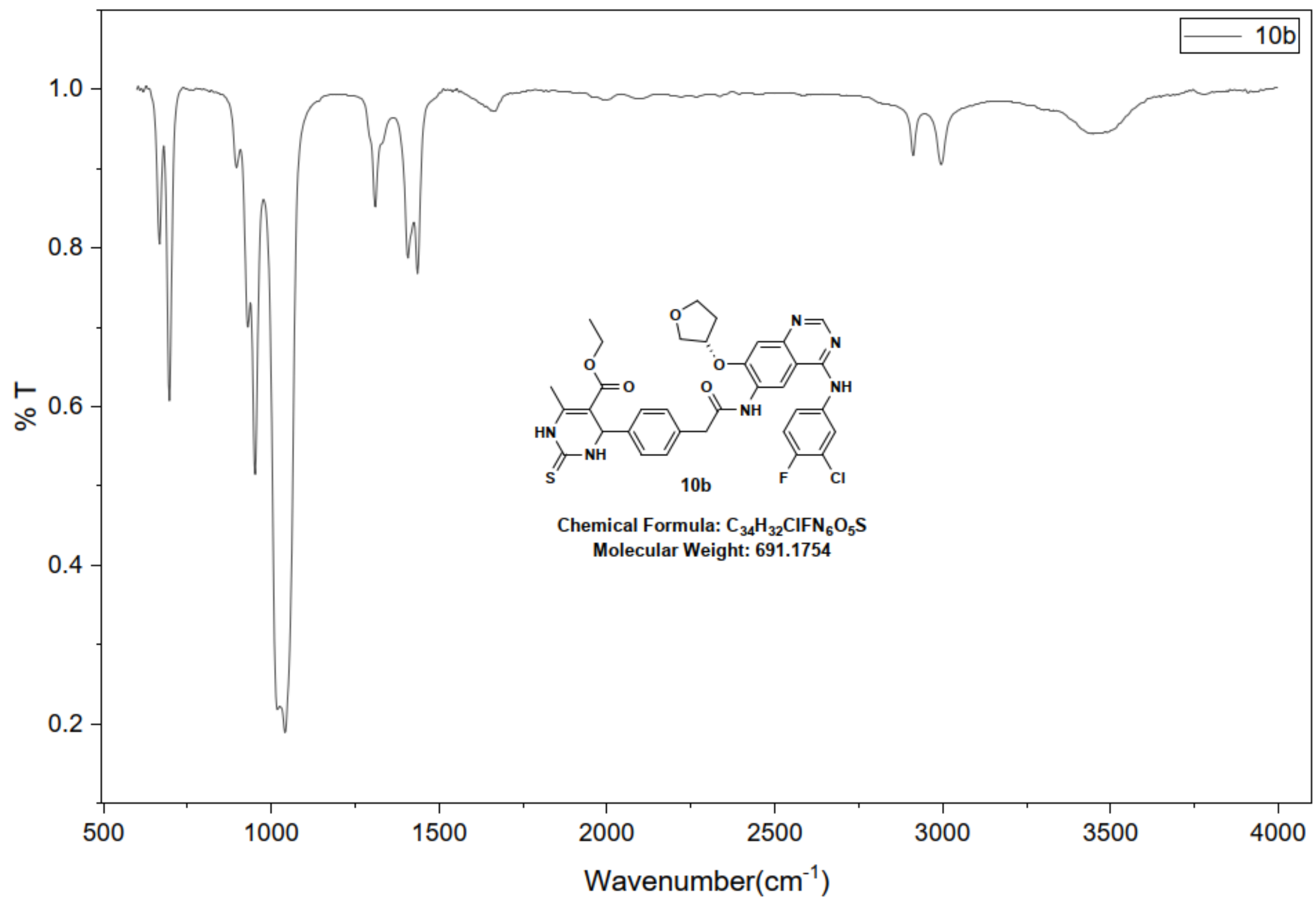

Fig. S19: IR spectrum of final compound 10c

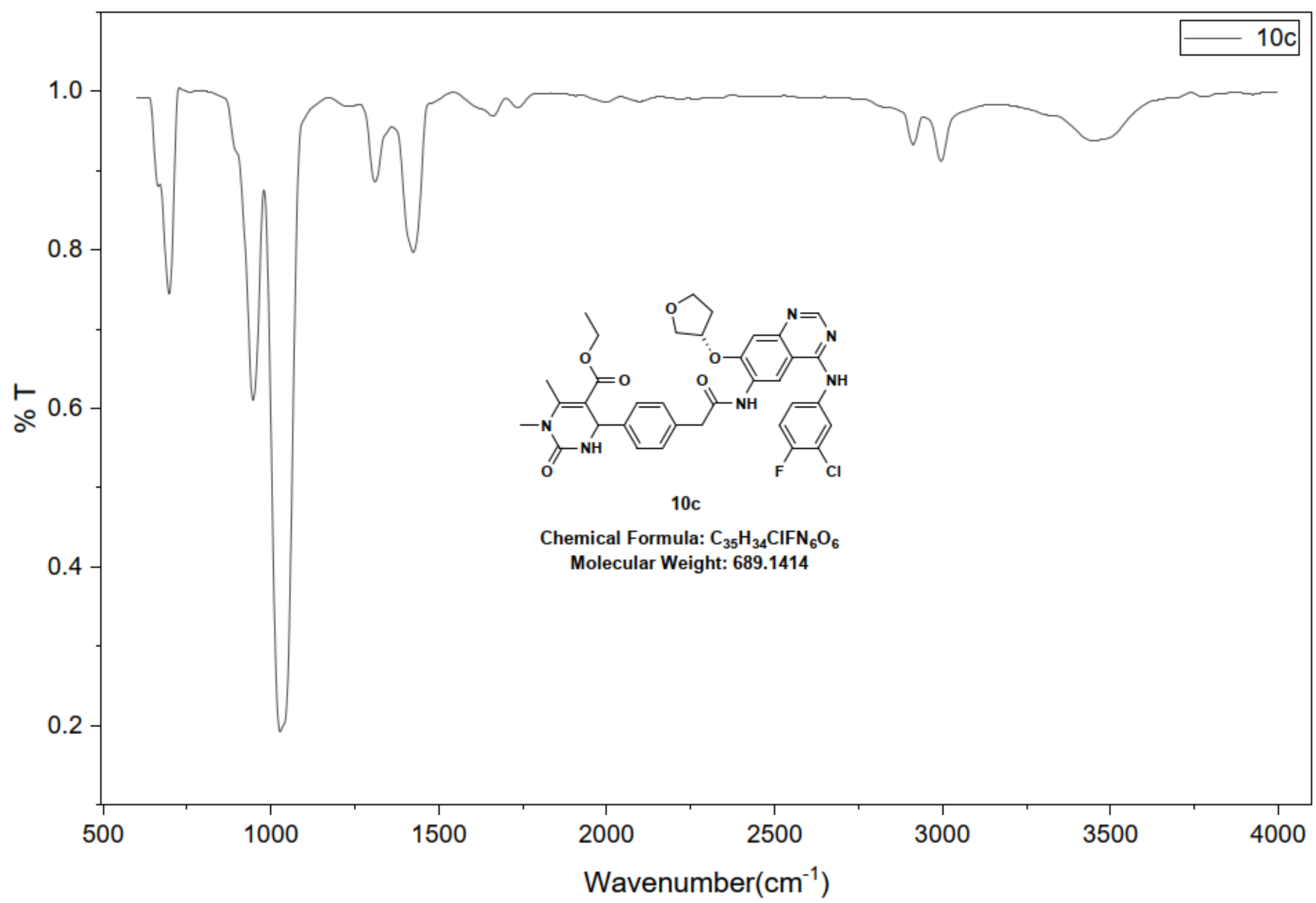

Fig. S20: IR spectrum of final compound 12a

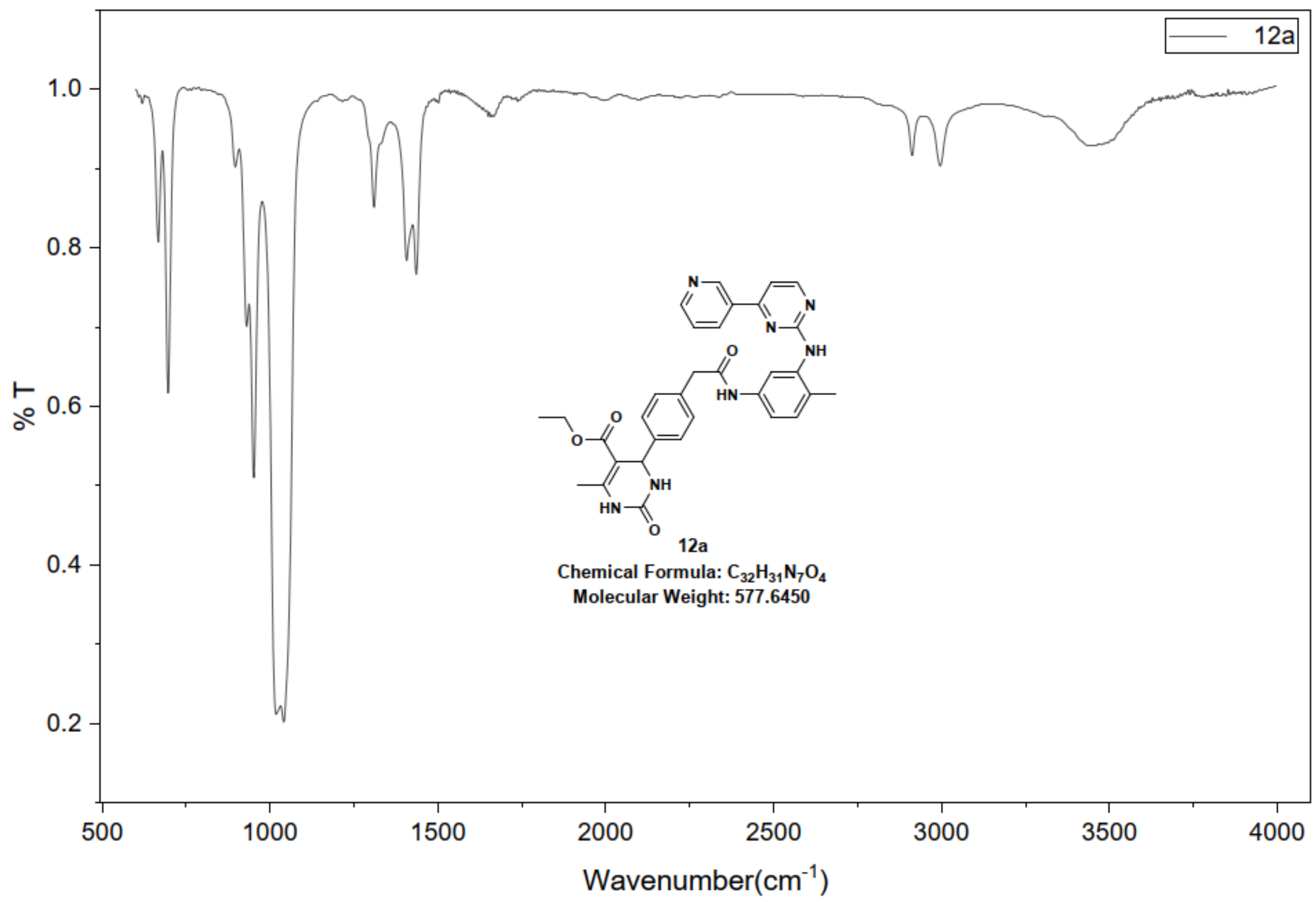

Fig. S21: IR spectrum of final compound 12b

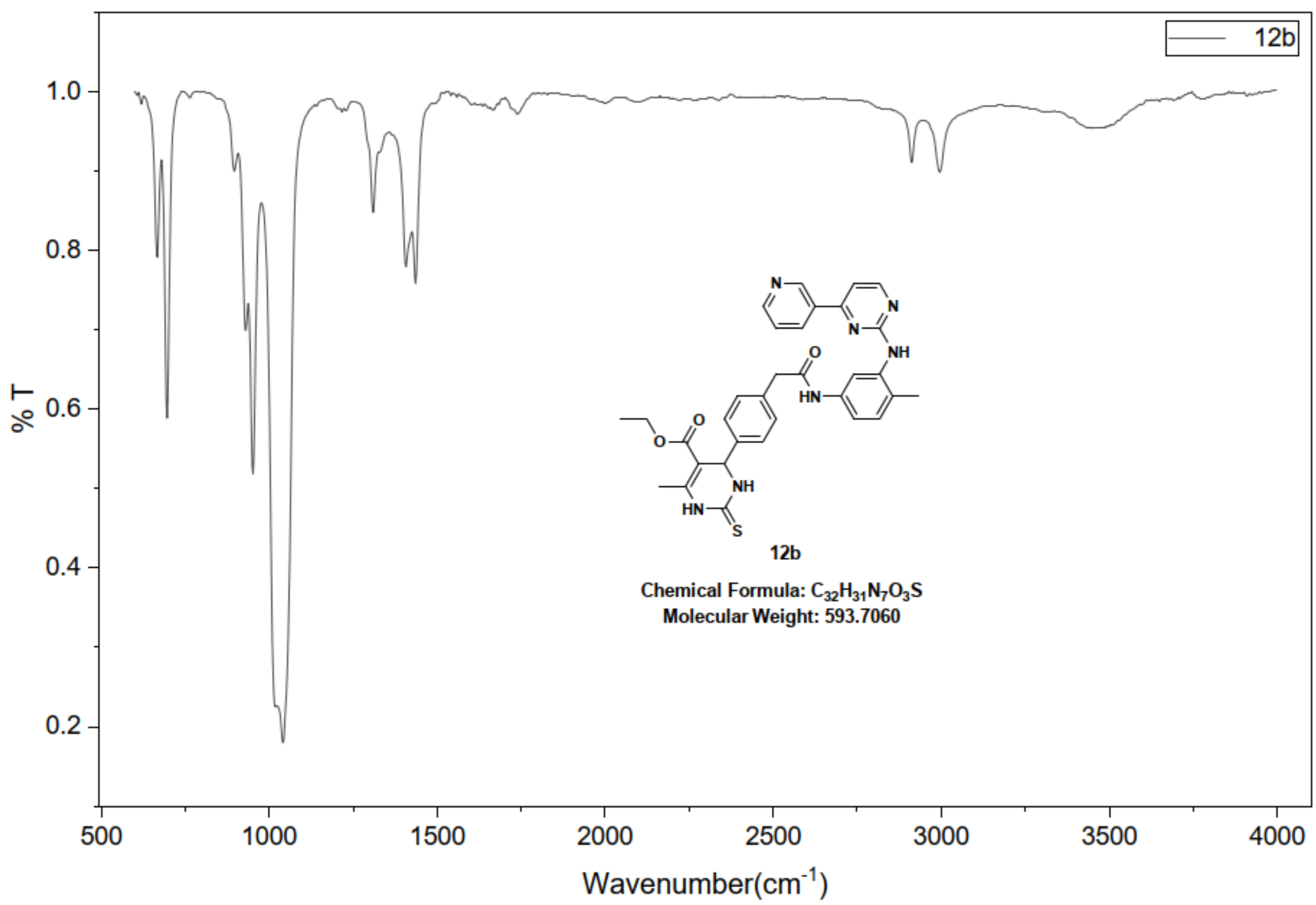

Fig. S22: IR spectrum of final compound 12c

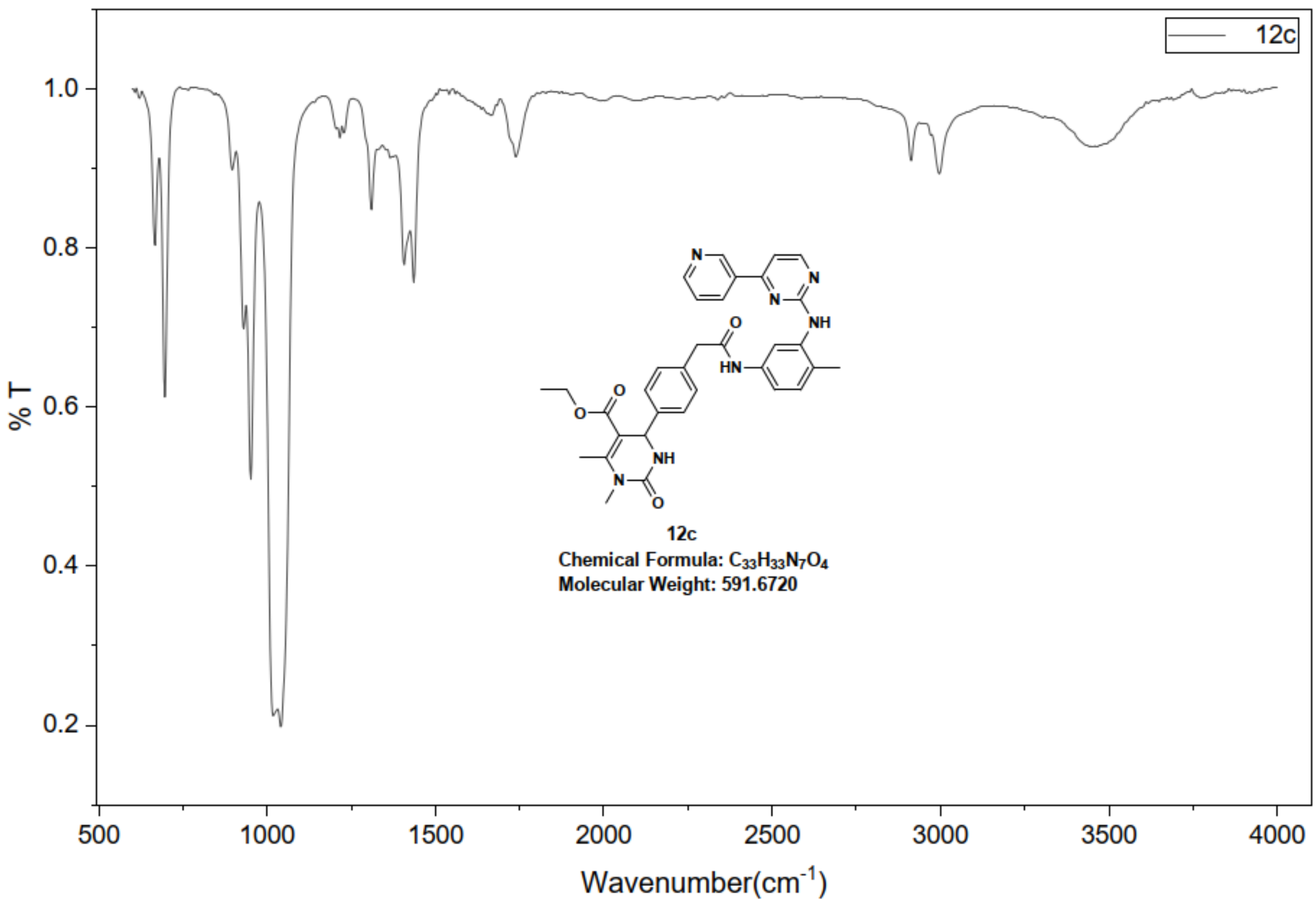

Fig. S23: ¹³C NMR spectrum of final compound 6a

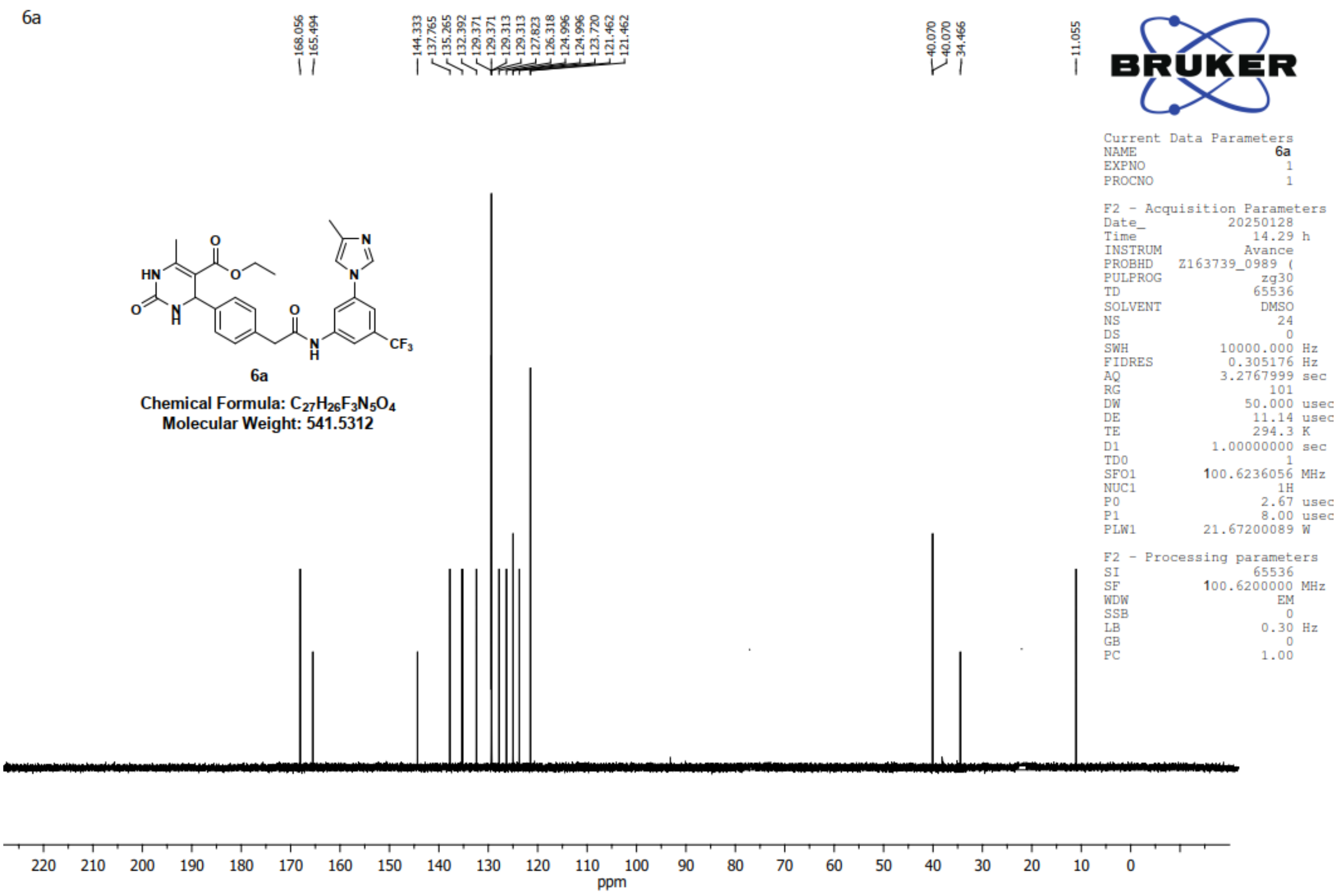

Fig. S24: ^{13}C NMR spectrum of final compound 6b

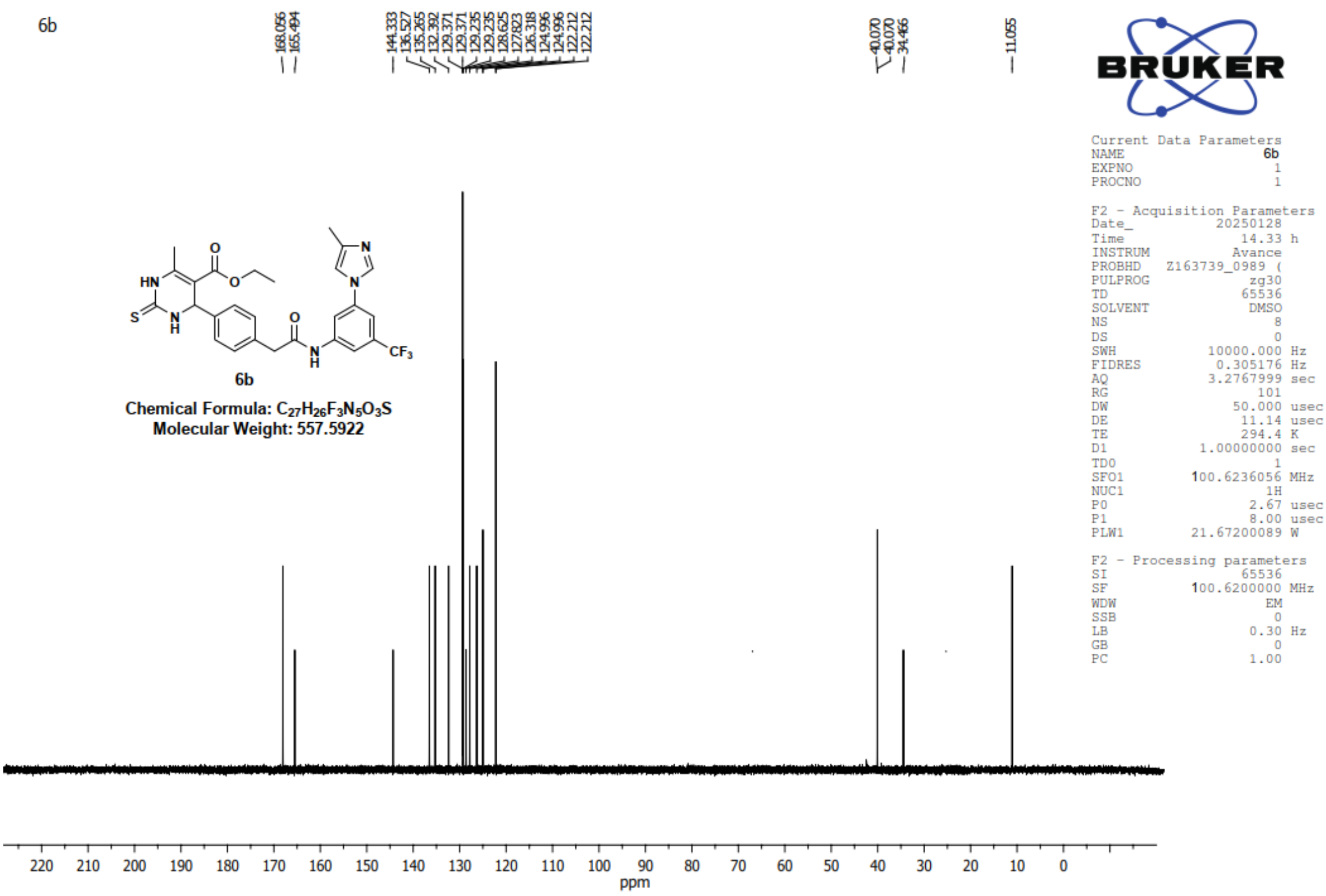

Fig. S25: ^{13}C NMR spectrum of final compound 6c

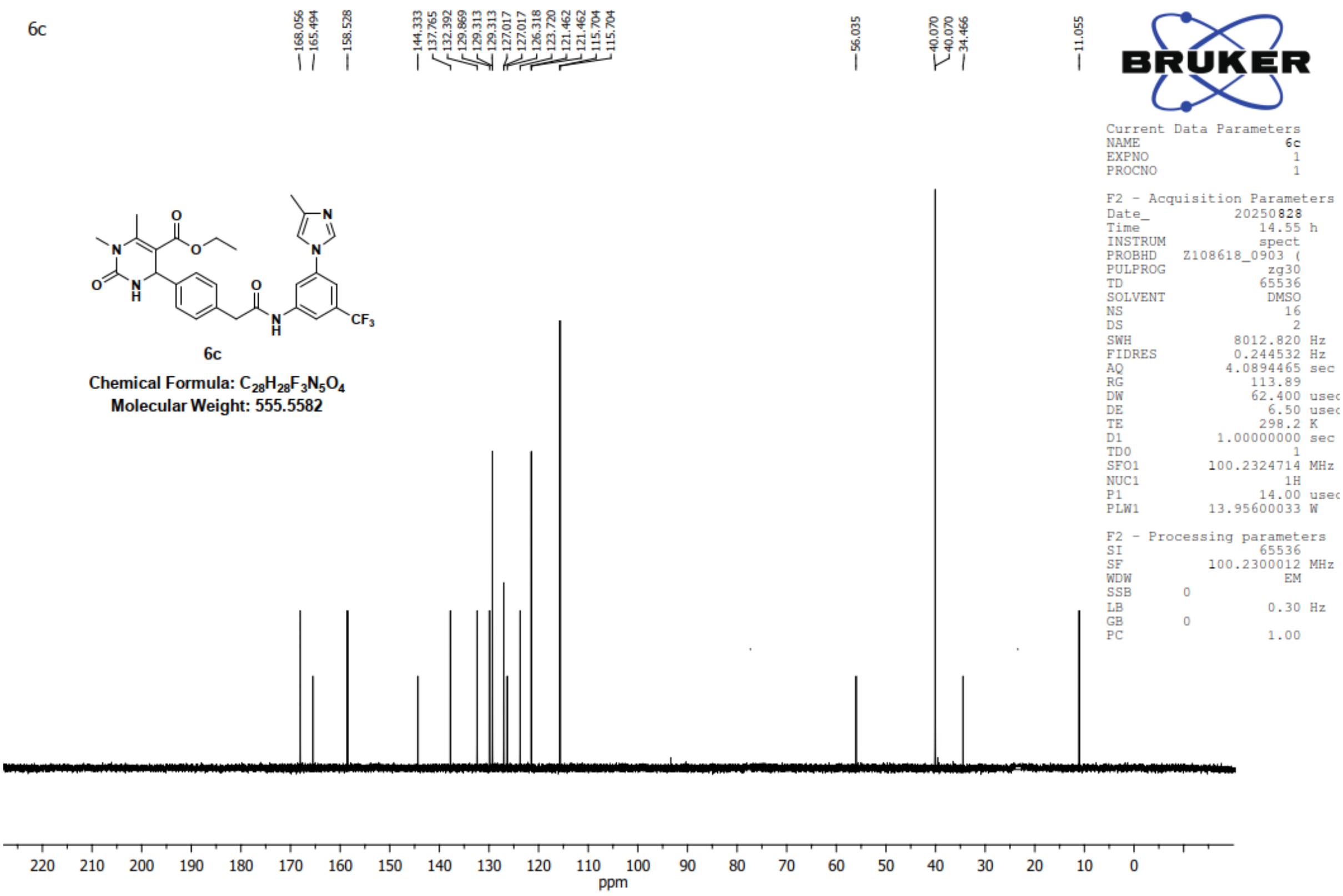

Fig. S26: ^{13}C NMR spectrum of final compound 8a

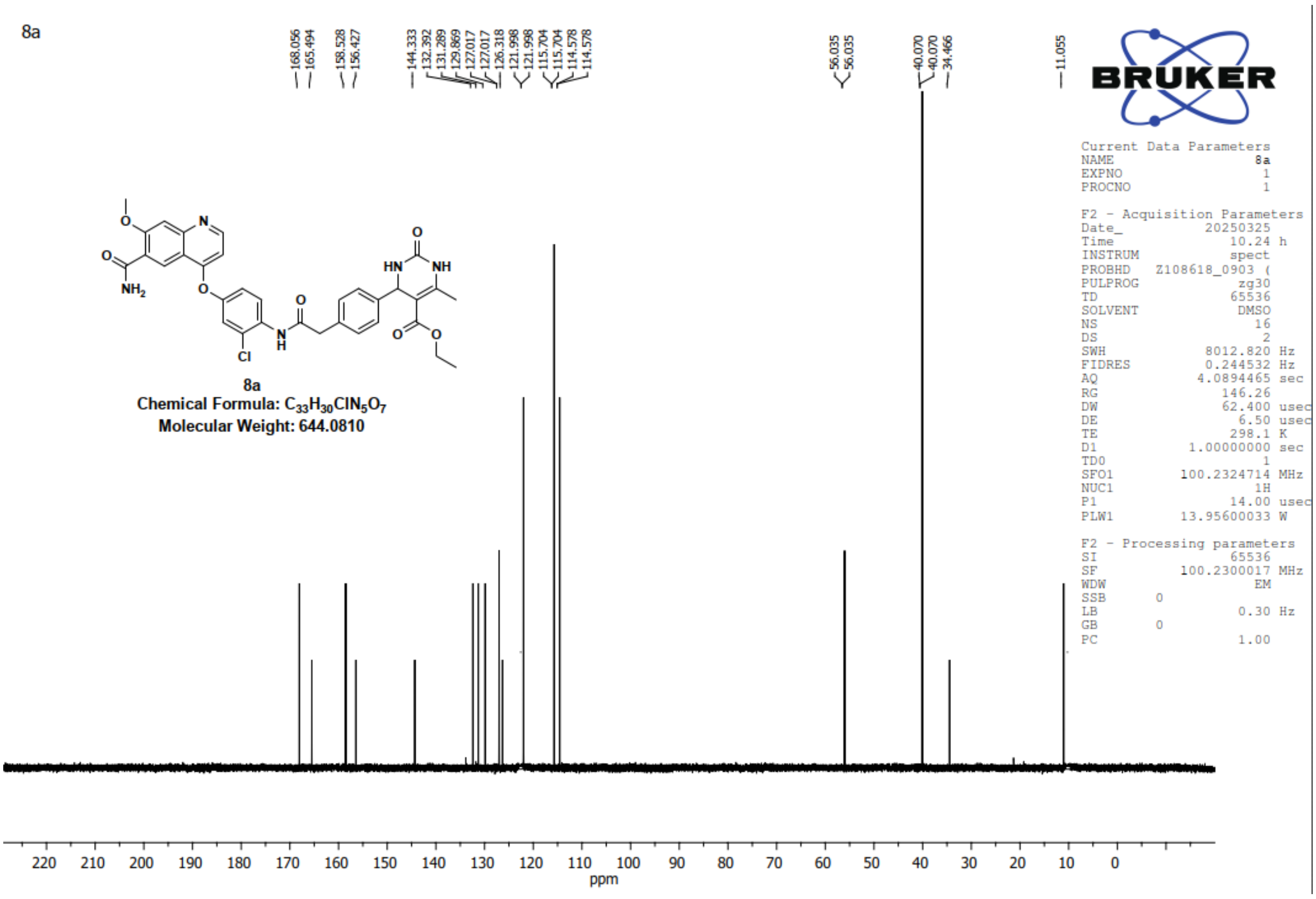

Fig. S27: ^{13}C NMR spectrum of final compound 8b

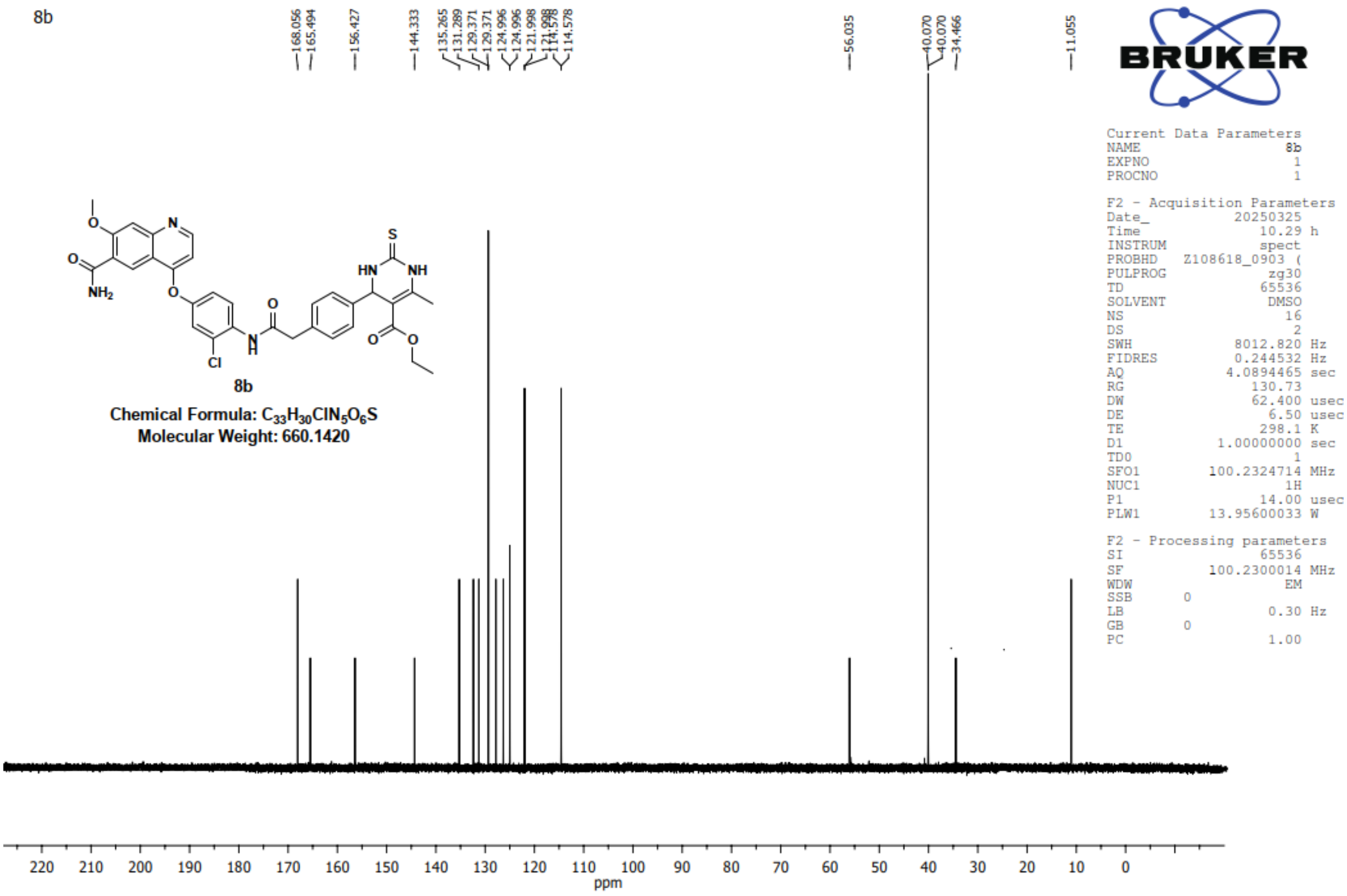

Fig. S28: ¹³C NMR spectrum of final compound 8c

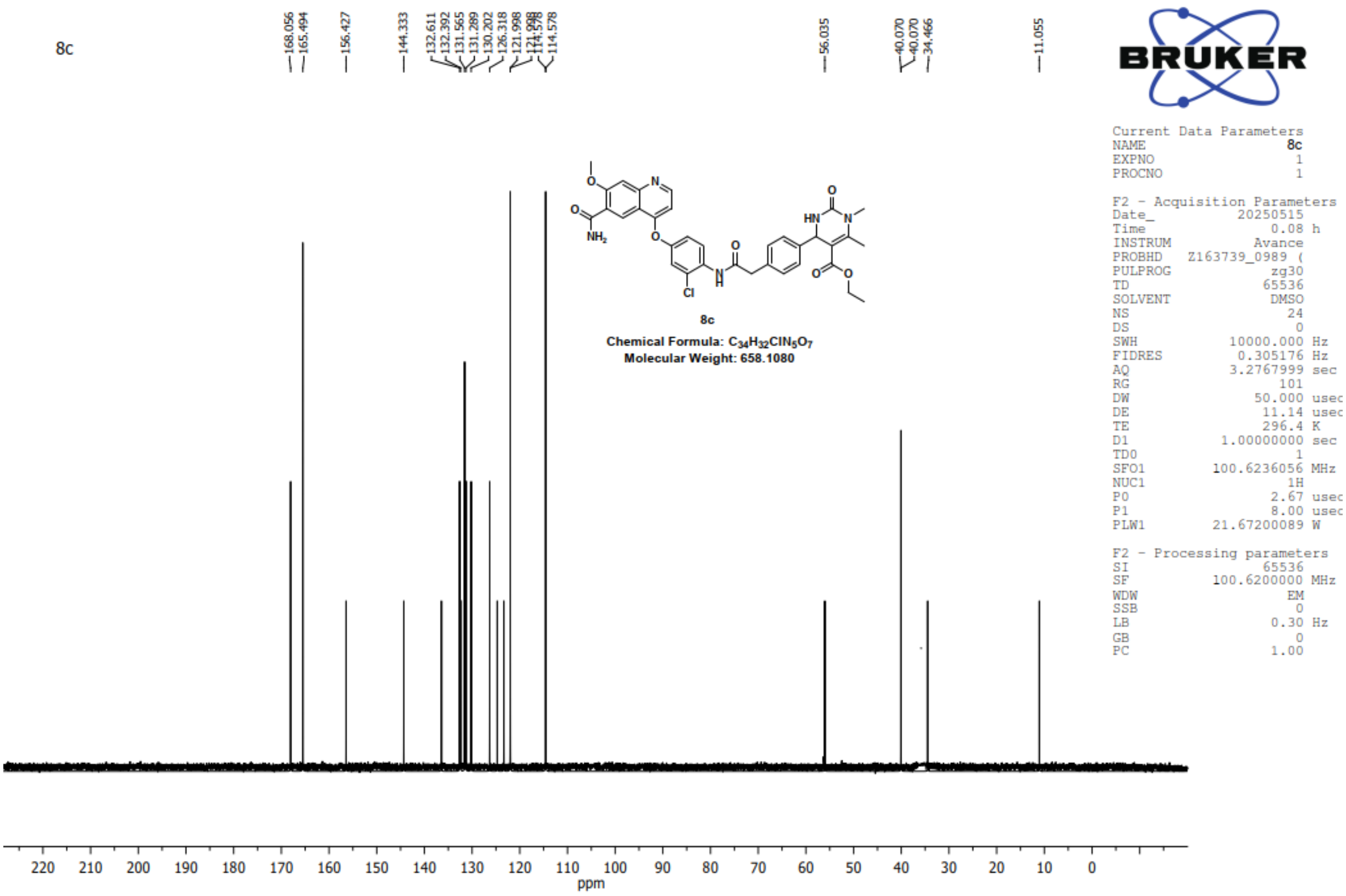

Fig. S29: ^{13}C NMR spectrum of final compound 10a

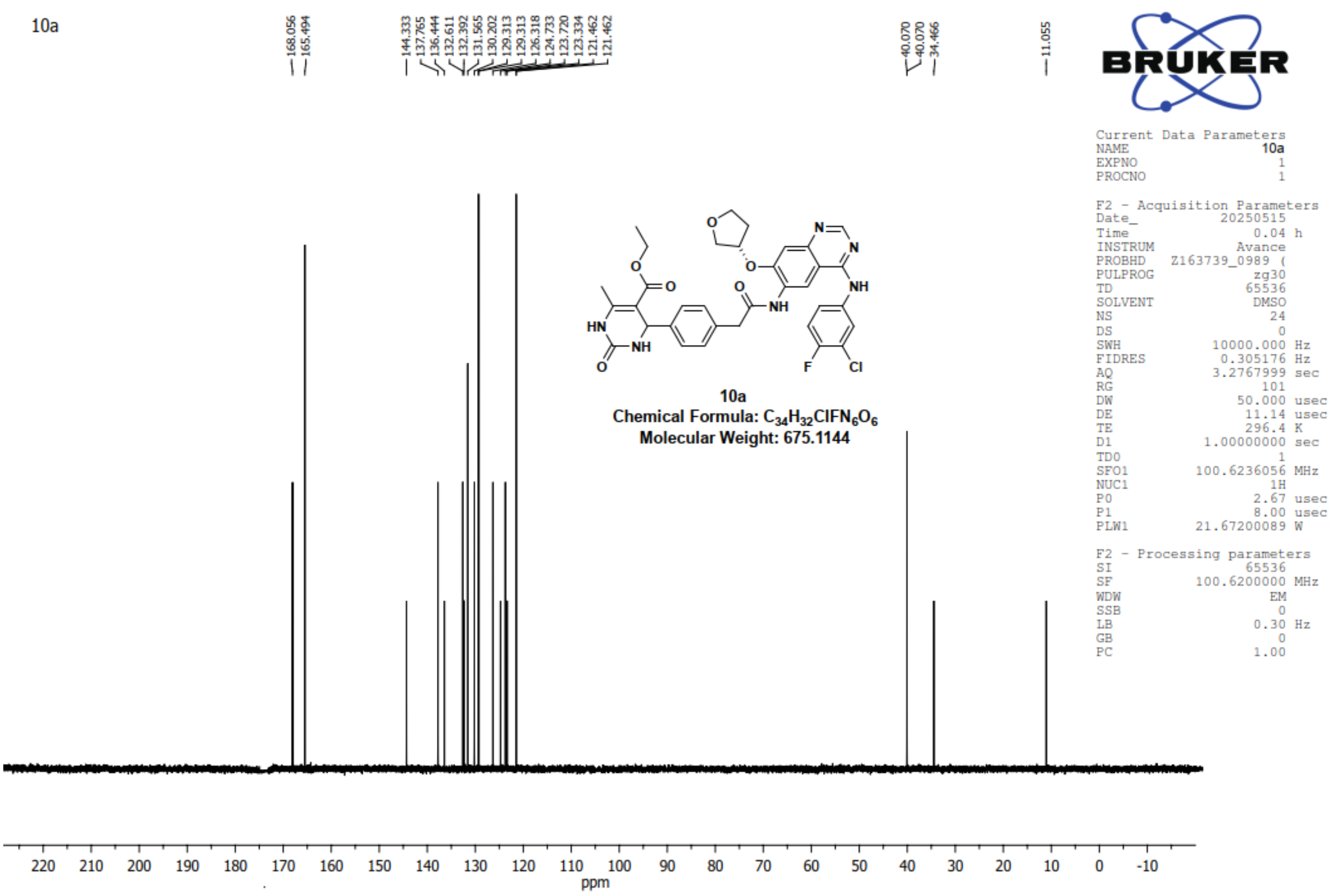

Fig. S30: ^{13}C NMR spectrum of final compound 10b

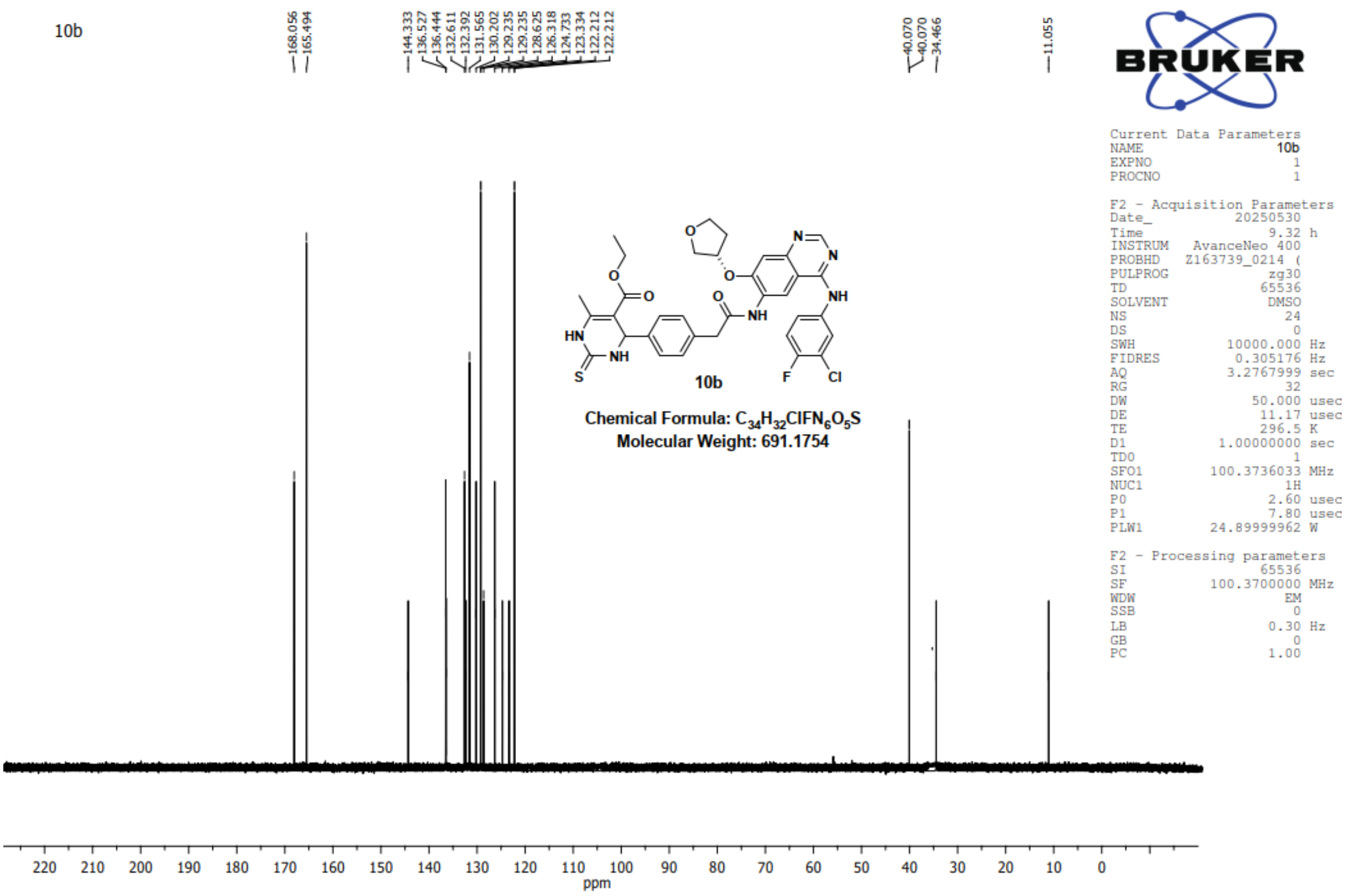

Fig. S31: ^{13}C NMR spectrum of final compound 10c

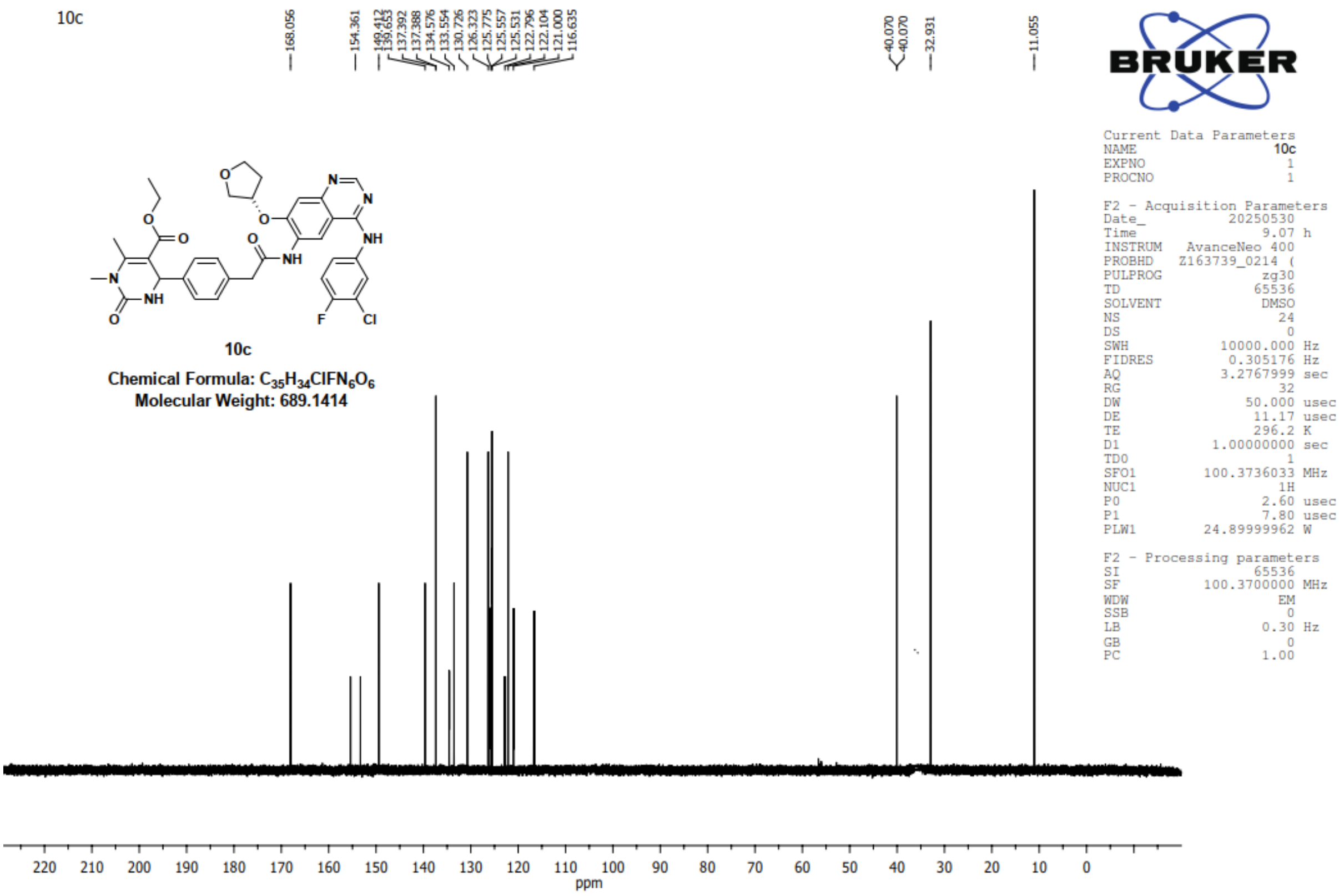

Fig. S32: ^{13}C NMR spectrum of final compound 12a

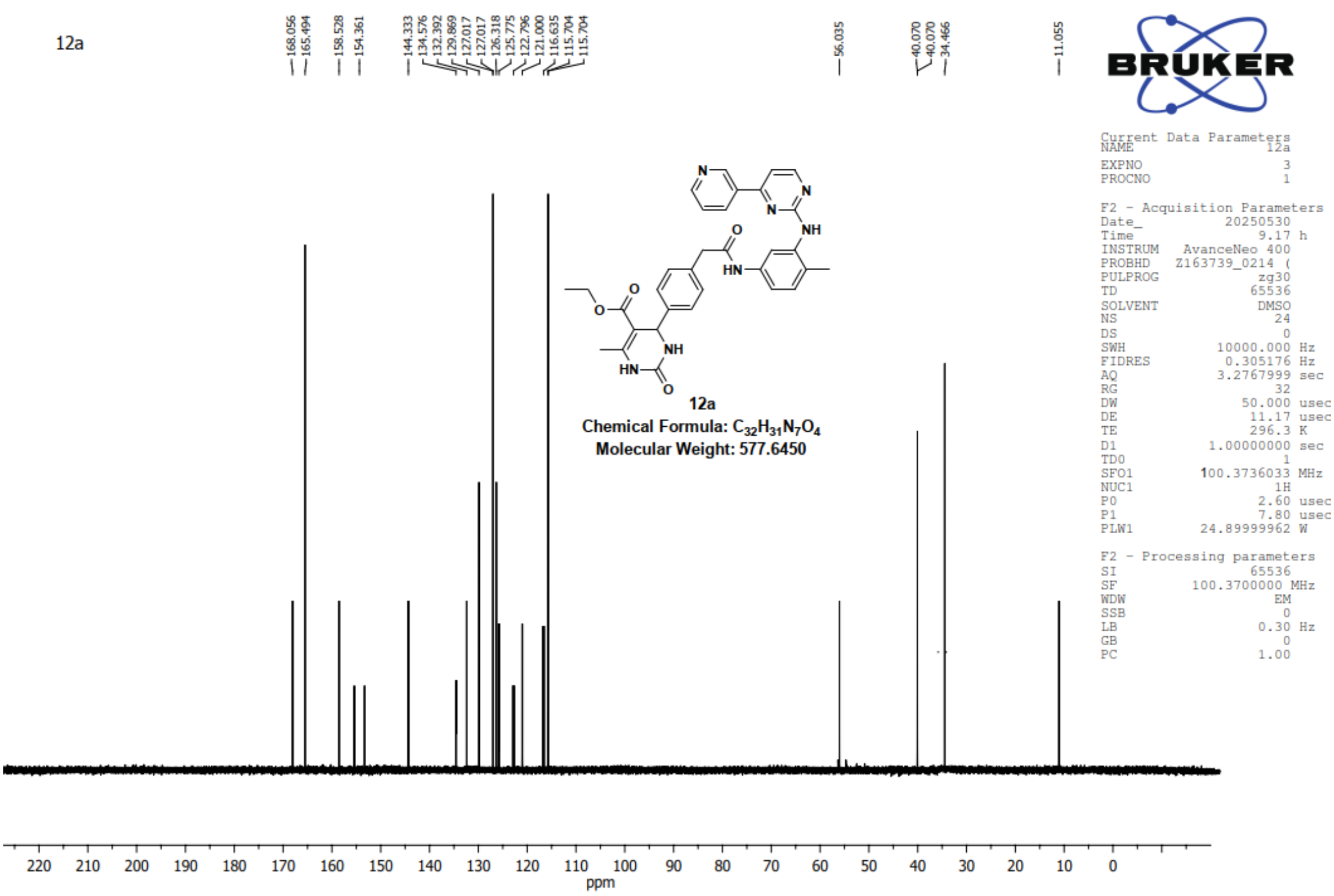

Fig. S33: ^{13}C NMR spectrum of final compound 12b

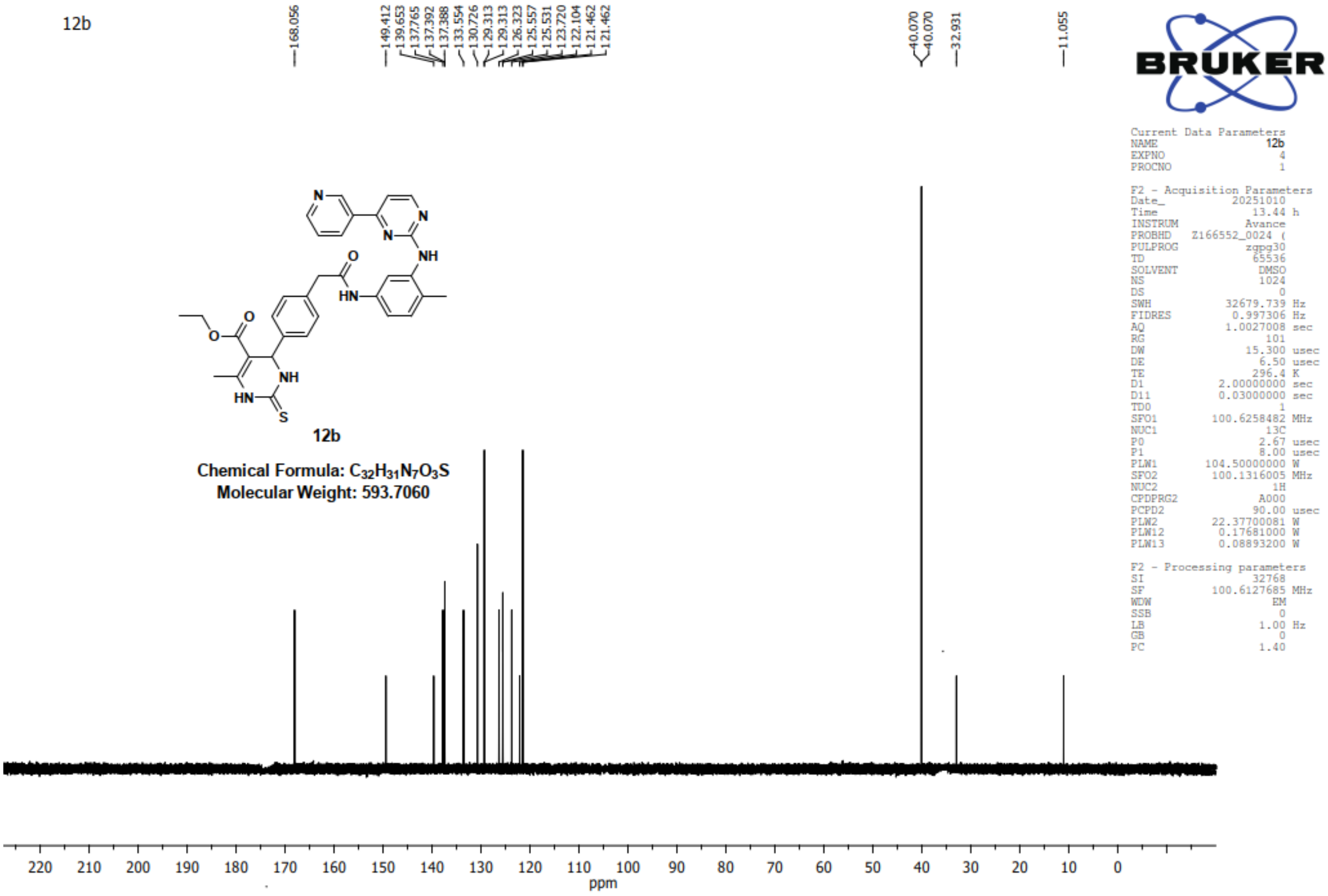

Fig. S34: ^{13}C NMR spectrum of final compound 12c

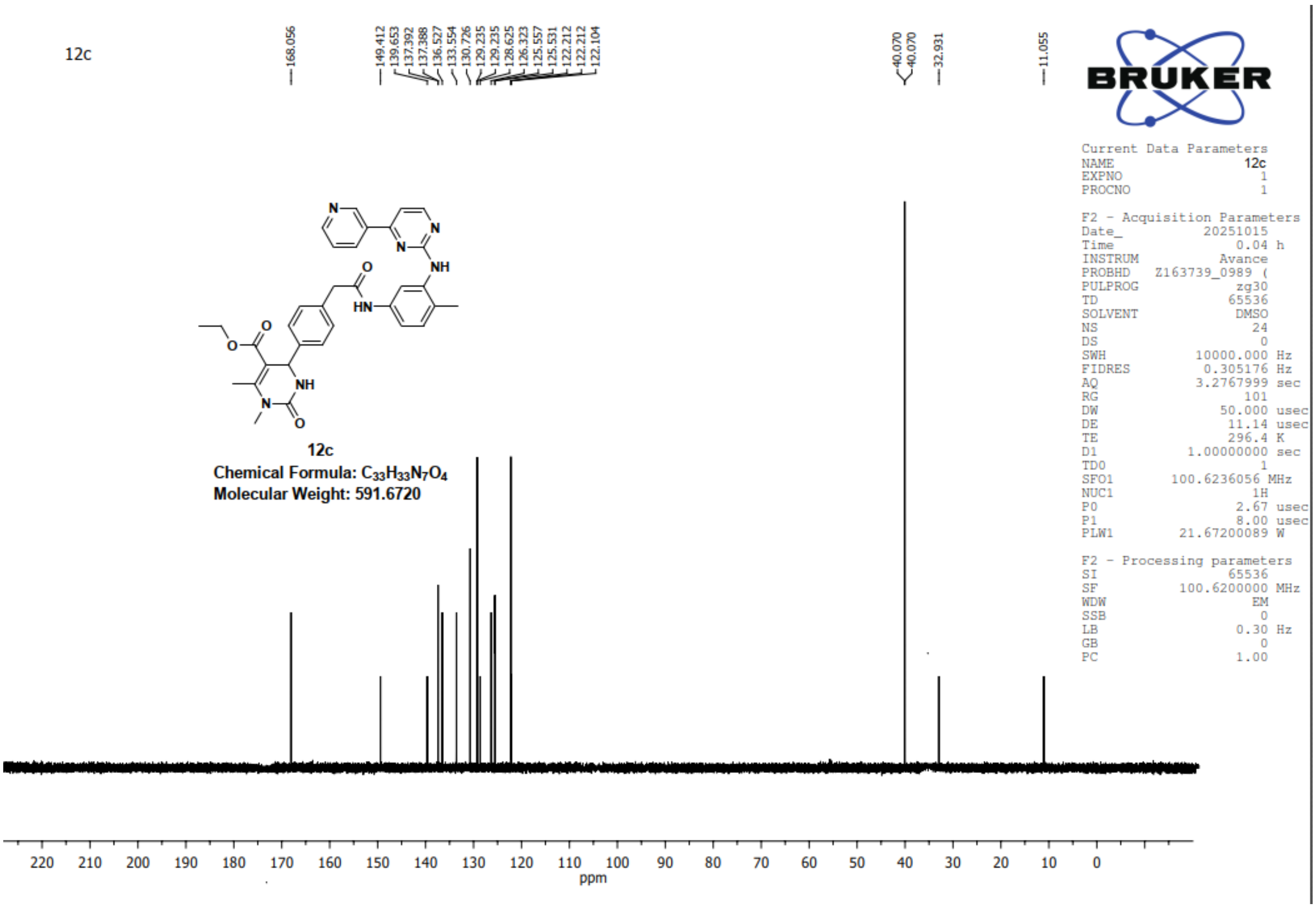

Fig. S35: Mass spectrum of final compound 6a

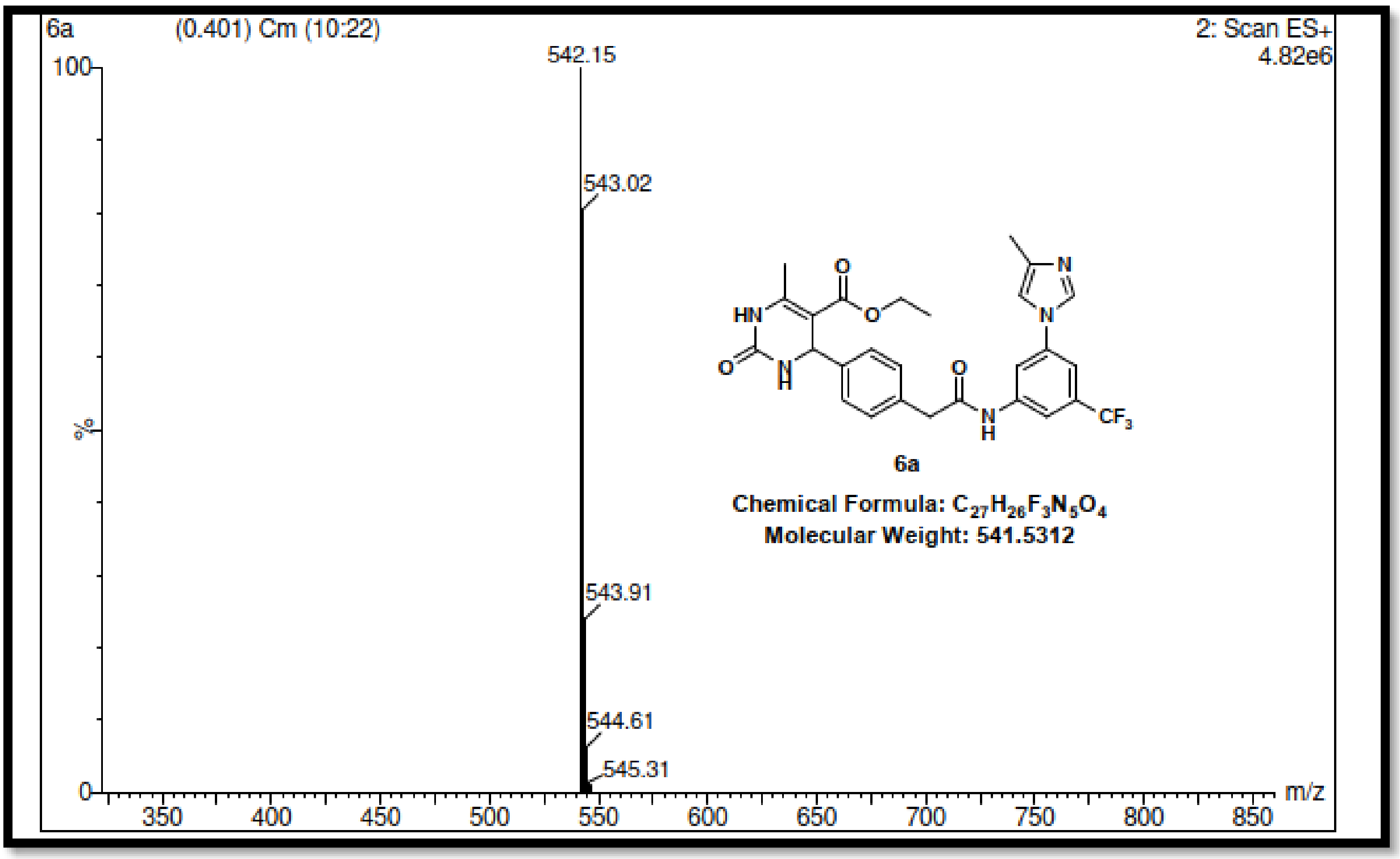

Fig. S36: Mass spectrum of final compound 6b

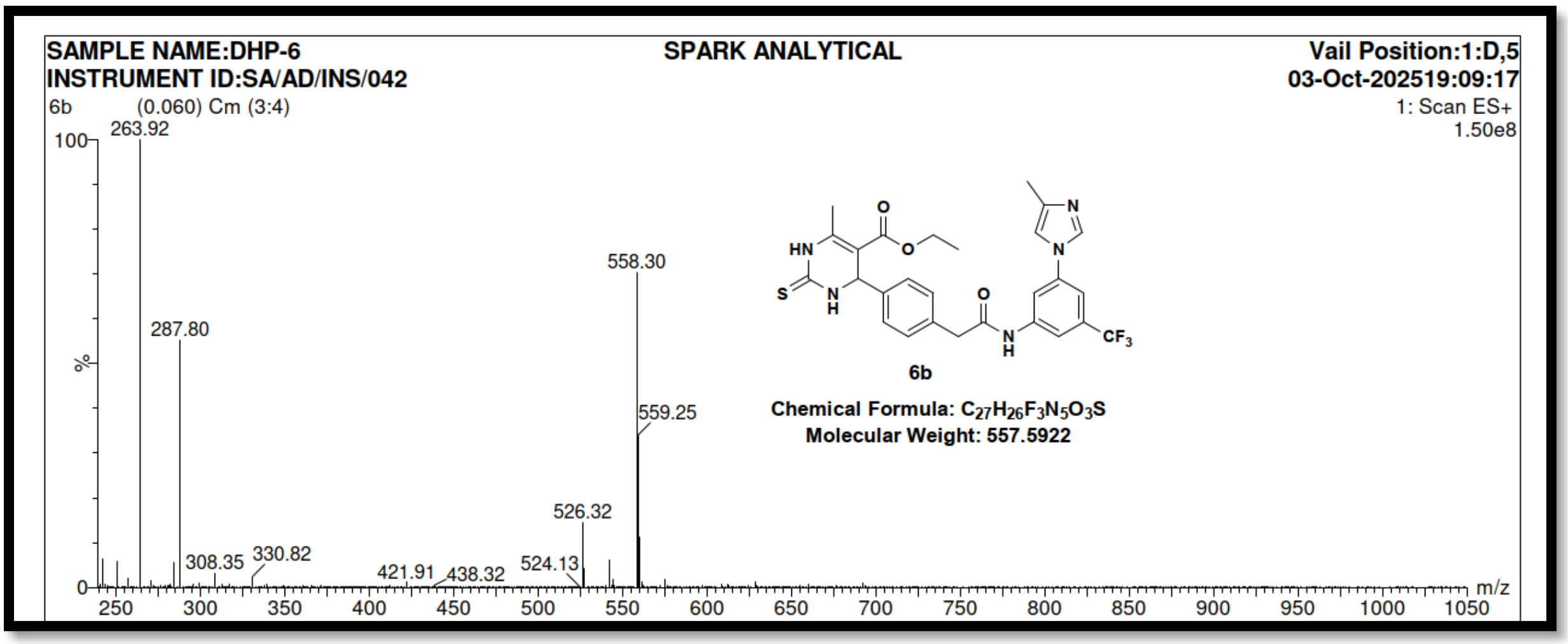

Fig. S37: Mass spectrum of final compound 6c

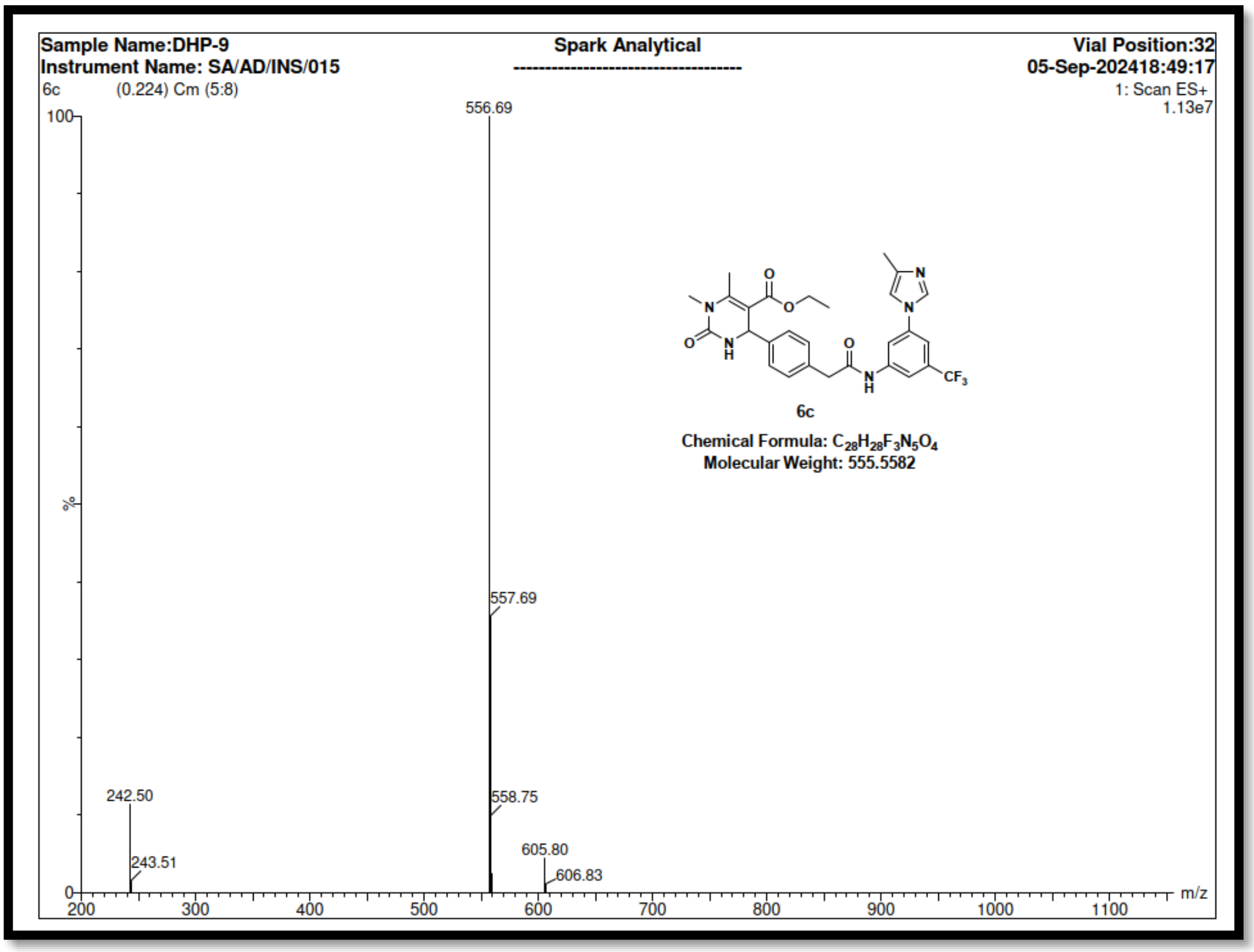

Fig. S38: Mass spectrum of final compound 8a

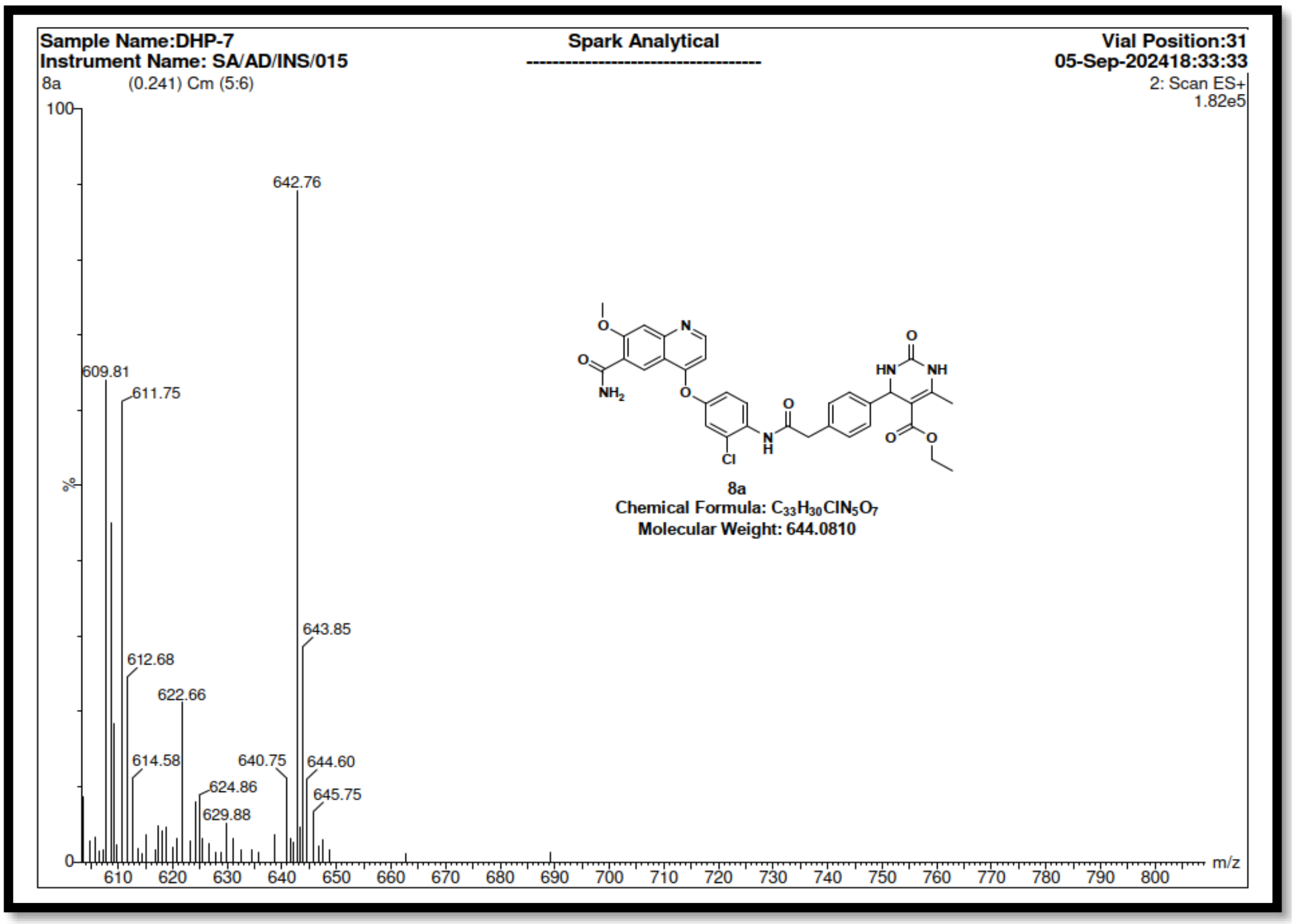

Fig. S39: Mass spectrum of final compound 8b

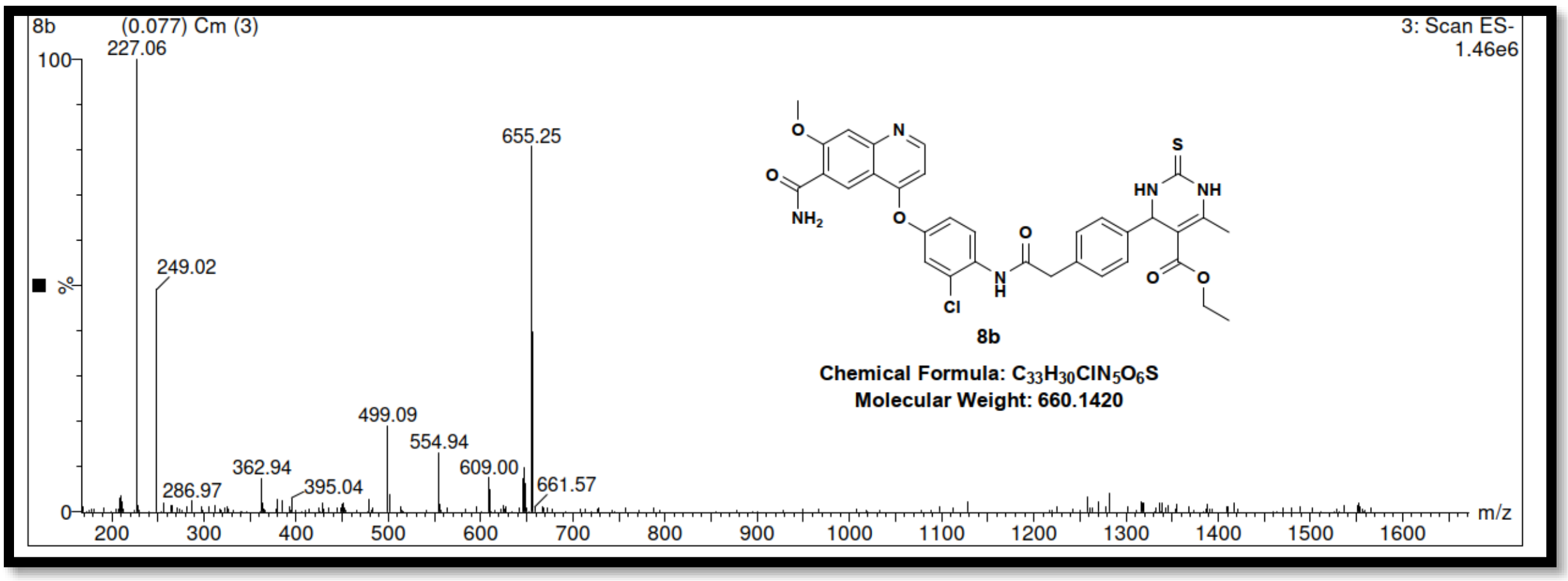

Fig. S40: Mass spectrum of final compound 8c

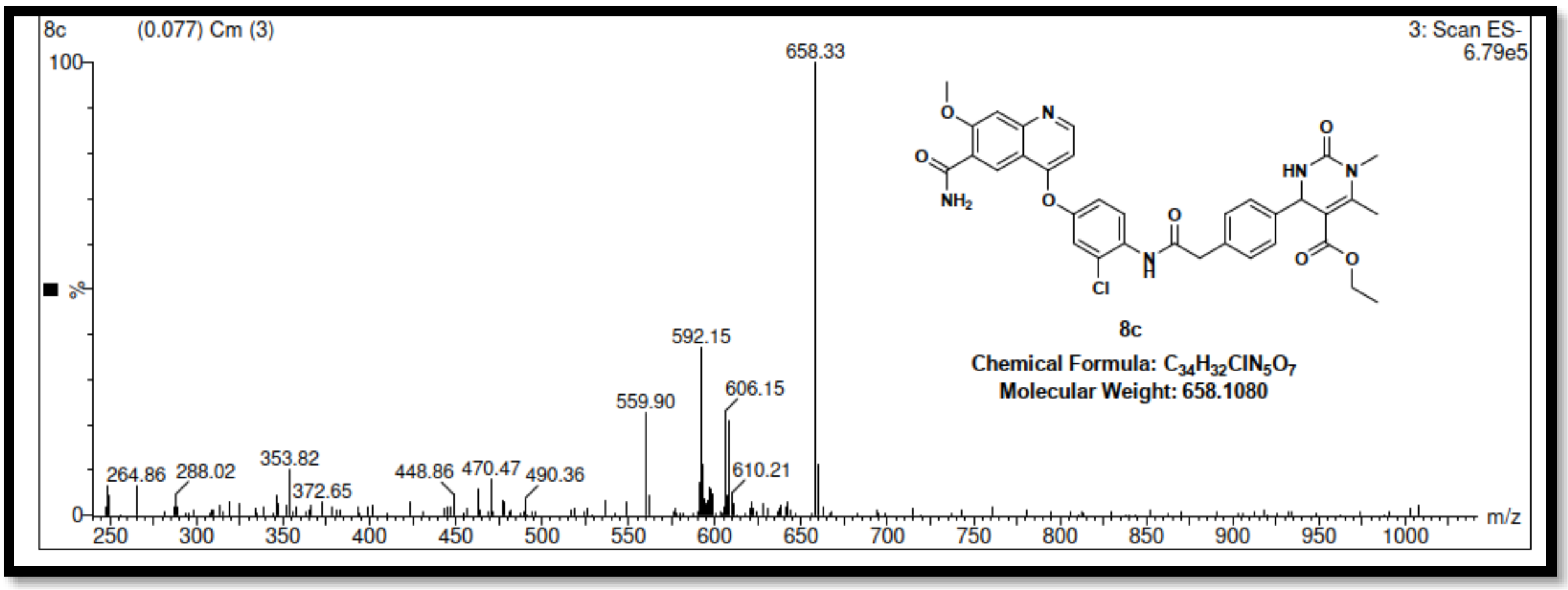

Fig. S41: Mass spectrum of final compound 10a

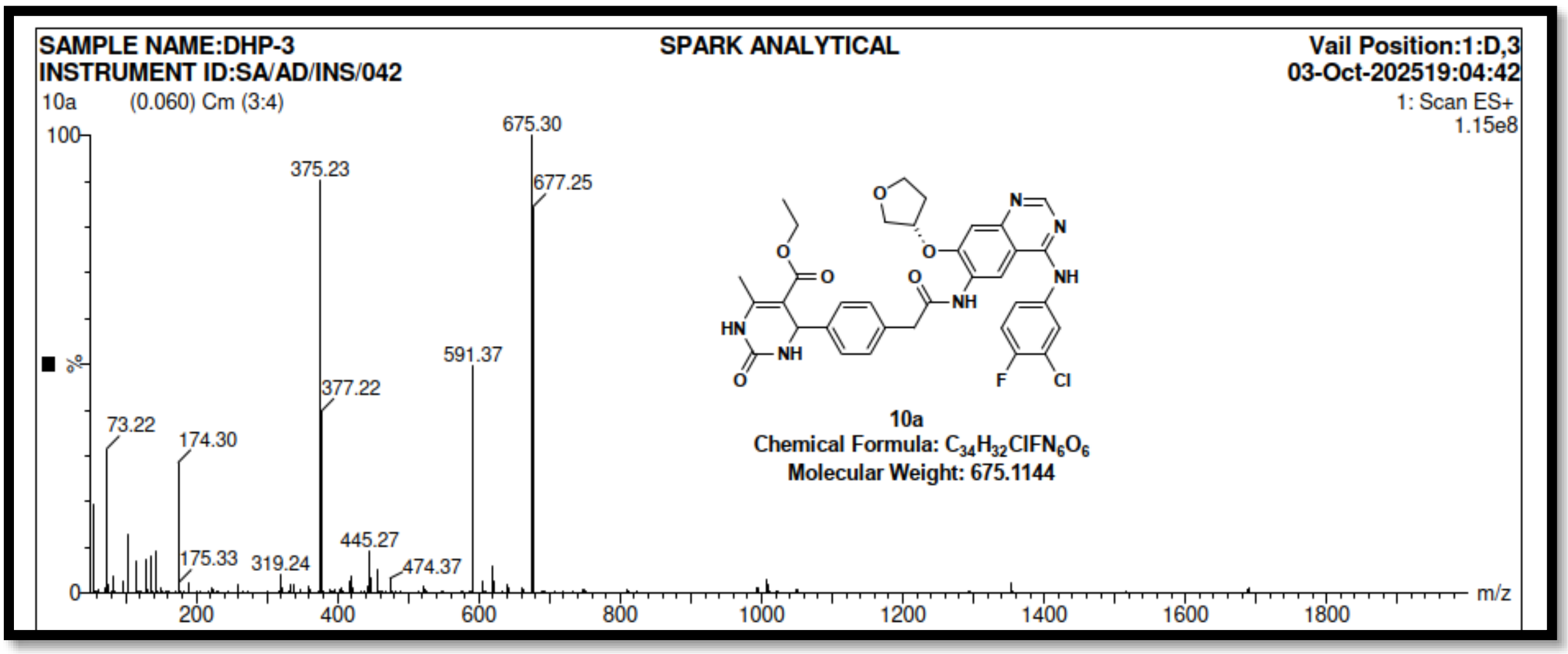

Fig. S42: Mass spectrum of final compound 10b

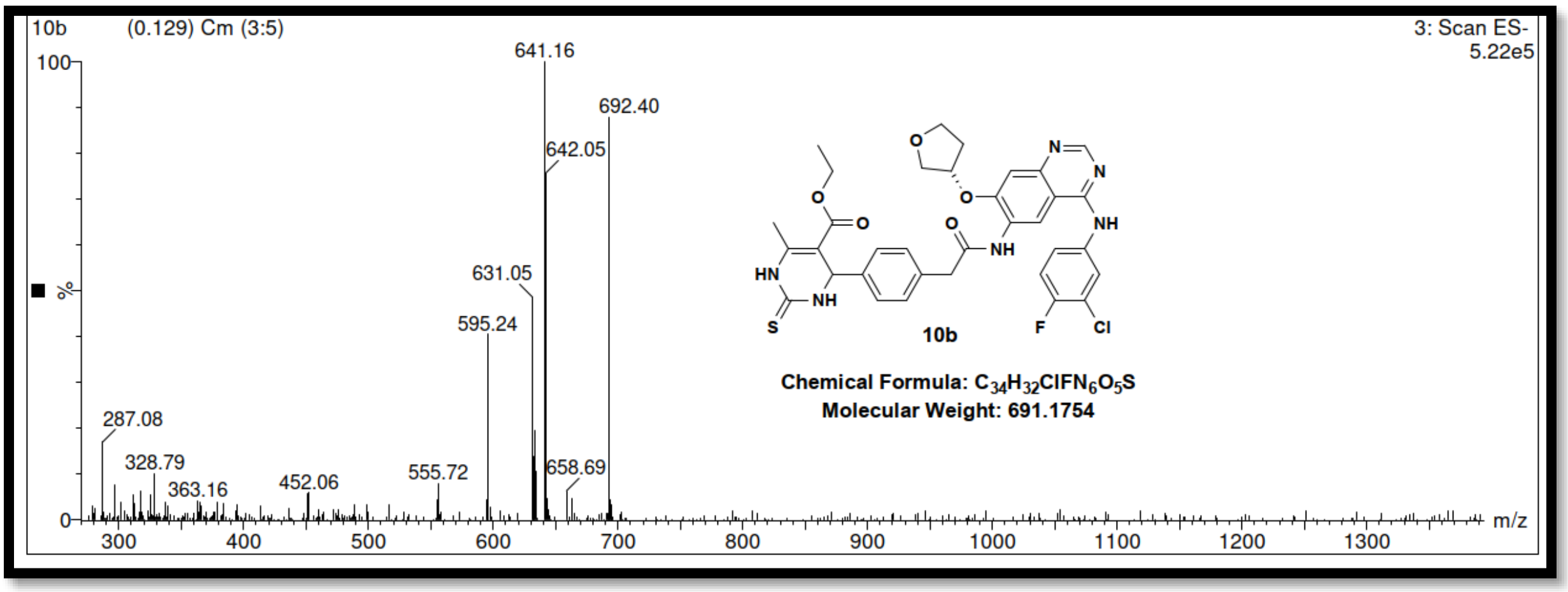

Fig. S43: Mass spectrum of final compound 10c

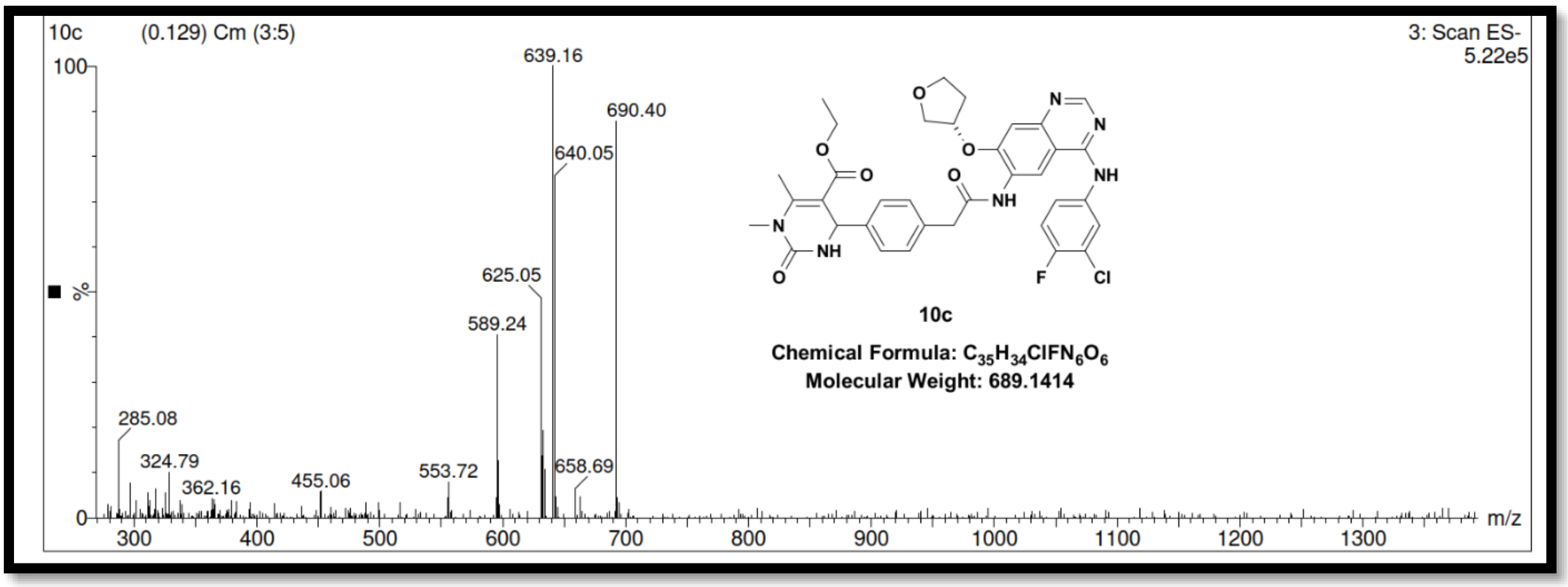

Fig. S44: Mass spectrum of final compound 12a

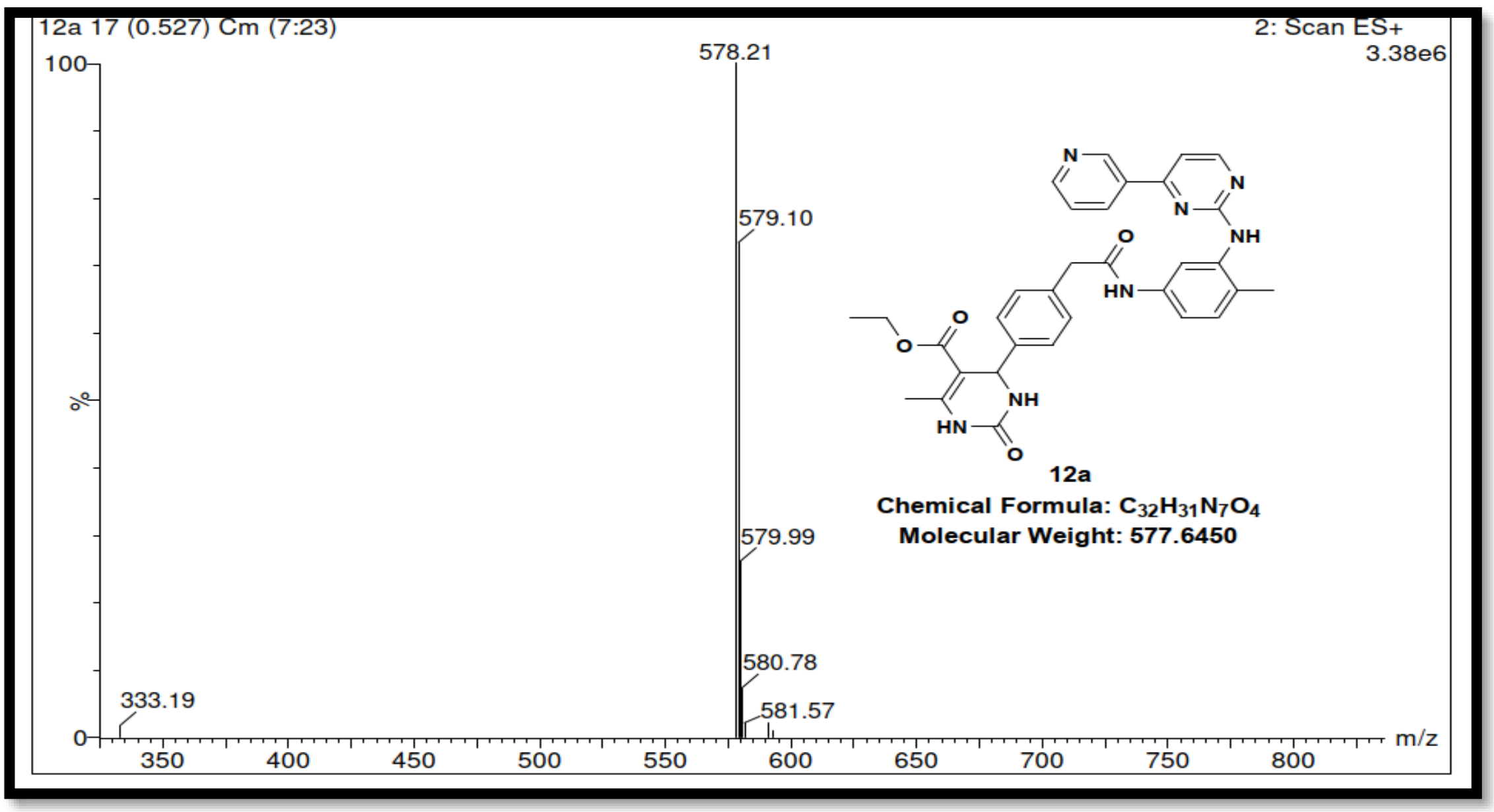

Fig. S45: Mass spectrum of final compound 12b

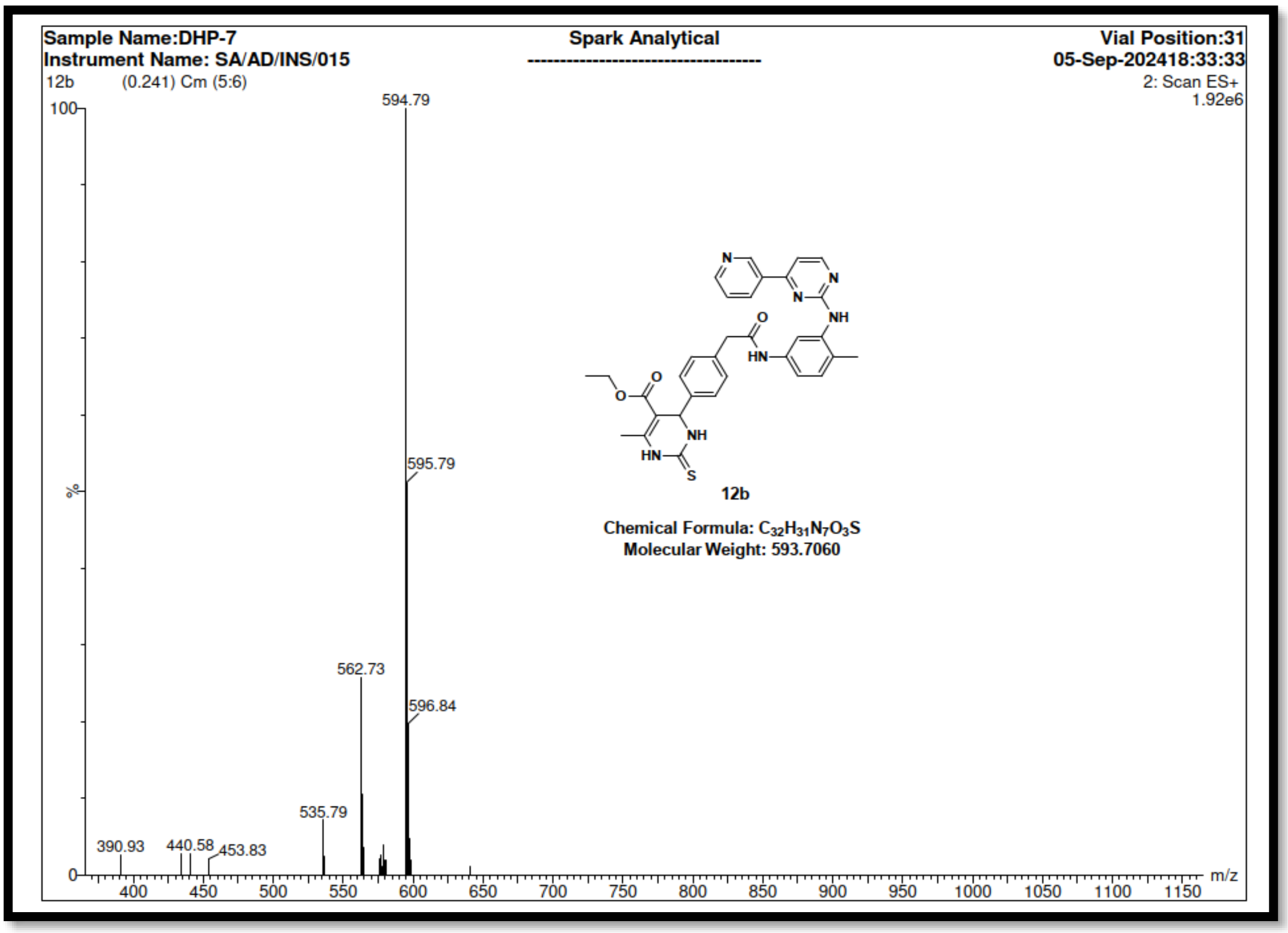

Fig. S46: Mass spectrum of final compound 12c

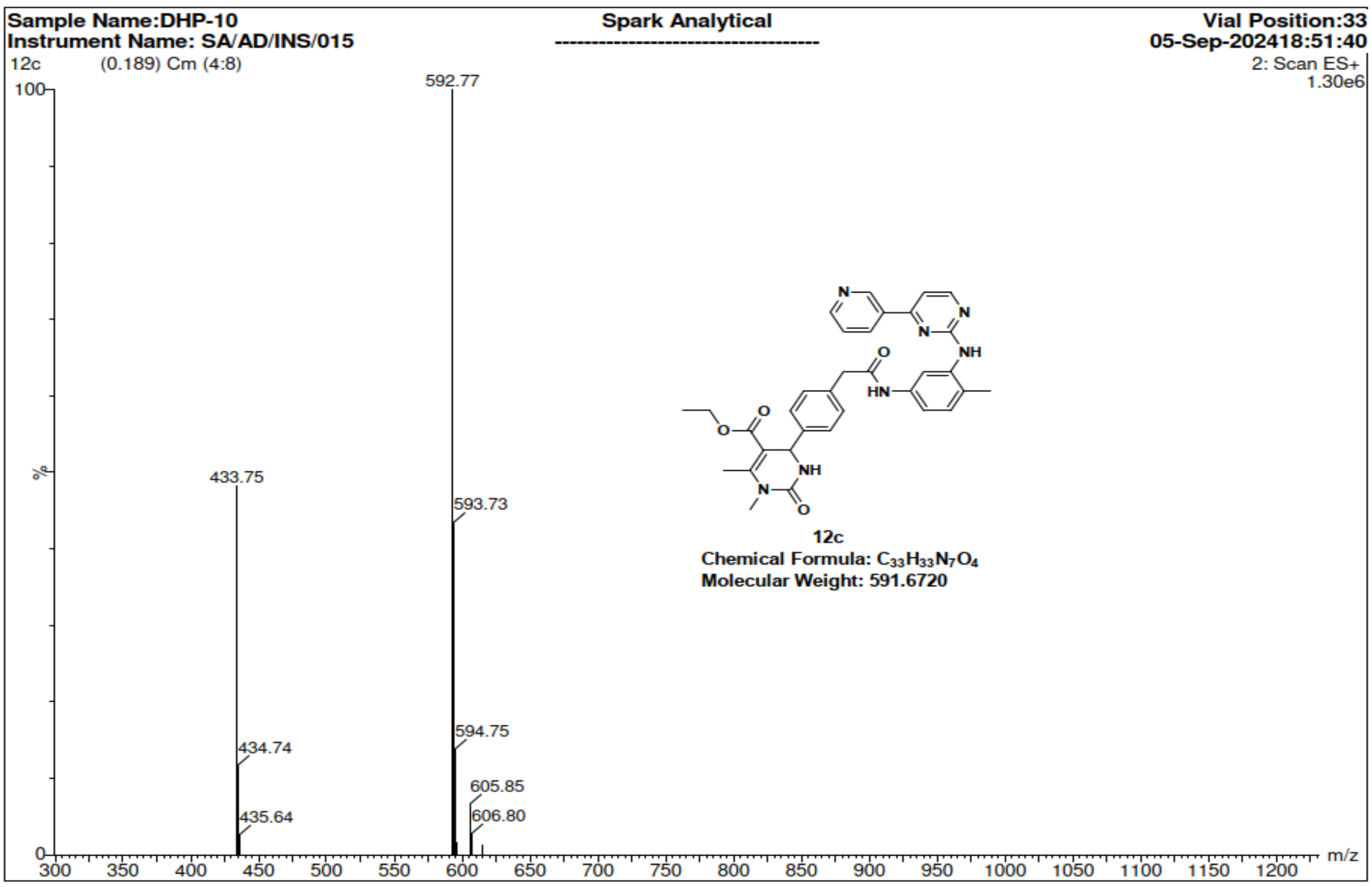

Anticancer activity:

Table S1. In vitro cytotoxic activity (IC₅₀, μg/mL) of synthesized compounds against A549 human lung carcinoma cell line.

Compound	IC₅₀ (μM)	IC₅₀ (μg/mL)	MW (g/mol)
6a	64.63	35.00	541.54
6b	146.34	81.60	557.61
6c	47.23	26.24	555.58
8a	76.48	49.26	644.09
8b	183.75	121.30	660.14
8c	40.22	26.47	658.13
10a	63.52	42.88	675.06
10b	23.31	16.11	691.12
10c	31.29	21.56	689.04
12a	202.20	116.80	577.65
12b	43.51	25.83	593.66
12c	181.35	107.30	591.67
Methotrexate	33.01	15.00	454.41

Values are expressed as mean ± SD (n = 3). Highlighted in green: most potent synthesized compound (10b). Highlighted in yellow: reference standard (methotrexate). Lower IC₅₀ = greater cytotoxic potency.

Molecular Docking studies:

Table S2. Molecular docking results of synthesized compounds (6a-c, 8a-c, 10a-c, 12a-c) and reference inhibitors against the EGFR tyrosine kinase domain (PDB ID: 1M17).

EGFR tyrosine kinase inhibitor (1M17)					
Compound code	Docking score (kcal/mol)	Interacting amino acids			
		Hydrophobic interactions	Polar interactions	H-bond	Others
6a	-6.128	LEU834, MET742, ALA719, LEU764, LEU694, LEU768, MET769, PRO770, LEU820, VAL702, PHE699, PRO853	THR830, ASN818, THR766, GLN767	MET769 (H-bond to imidazole N)	LYS721, ARG817, LYS851 (charged positive); ASP813, ASP831, GLU738 (charged negative); GLY833, GLY772 (glycine); no halogen bond
6b	-5.956	LEU834, MET742, ALA719, LEU764, LEU694, LEU768, MET769, PRO770, LEU820, VAL702, PHE699, PRO853	THR830, ASN818	ASP831 (H-bond backbone – salt bridge to phenyl ring)	LYS721 (charged positive); ASP813, GLU738 (charged negative); ALA719
6c	-7.119	LEU834, GLY833, LEU768, MET769, PRO770, GLY772, LEU820, PHE699, VAL702	THR830, ASN818, GLN767, THR766	ASP831 (backbone); ASN818–LYS721 (H-bond sidechain, red line)	LYS721, ARG817 (charged positive); GLU738 (charged negative); MET769 (H-bond sidechain)
8a	-6.788	TRP856, PRO890, PRO853, LEU775, LEU694, GLY695, VAL702, PHE699, GLY772, CYS773, PRO770, MET769, LEU768	ASN818, THR830	ASN818 (H-bond backbone); LEU768 (H-bond sidechain); ARG779–NH_2 (H-bond)	ARG817, ARG779, LYS889 (charged positive); ASP813, GLU882, ASP776 (charged negative); Cl halogen bond; ASN818–Cl halogen bond

**8b**	-7.587	GLY772, CYS773, LEU694, MET769, LEU768, GLN767, THR766, LEU764, CYS751, MET742, GLU738, VAL702, PHE699, LEU820	ASN818, ALA698	MET769 (H-bond sidechain); ALA698–NH2 (H-bond)	LYS721, ARG817 (charged positive); ASP813, ASP831, GLU734 (charged negative); Pi-cation: Lys721; Cl halogen
**8c**	-6.334	LEU723, GLY833, ALA719, ALA698, PHE699, VAL702, CYS773, GLY772, LEU694, LEU820, LEU768, MET769, CYS751	ASN818, THR830, GLN767, THR766	GLU738–NH_2 (H-bond sidechain); LYS721 (H-bond backbone)	LYS721, ARG817 (charged positive); ASP831, GLU738, GLU734 (charged negative); Cl halogen
**10a**	-6.245	LEU834, GLY833, ALA719, MET742, LEU764, ILE765, MET769, LEU768, GLY772, CYS773, LEU694, PHE699, VAL702, LEU820	THR830, ASN818, THR766	ASP831 (H-bond backbone/sidechain); LYS721 (Pi-cation, red line); THF ring–ASP776	LYS721, ARG817, LYS851 (charged positive); ASP813, GLU738, ASP776 (charged negative); F, Cl halogen; ILE720
**10b**	-7.819	GLY833, LEU834, ALA719, ILE720, MET742, LEU764, ILE765, LEU768, MET769, GLY772, CYS773, LEU694, PHE699, VAL702, LEU820, LEU820	THR830, ASN818	ASP831 (H-bond sidechain); LYS721 (H-bond backbone, Pi-cation); THF ring–ASP776	LYS721, ARG817, LYS851 (charged positive); ASP813, GLU738, ASP776 (charged negative); F, Cl halogen; ILE765 (halogen bond)
**10c**	-6.843	MET742, LEU764, MET769, PRO770, PHE771, GLY772, CYS773, LEU694, LEU768, GLN767, THR766	ASN818, THR830	ASP831 (halogen bond to Cl); MET769 (H-bond sidechain)	ARG817 (charged positive); GLU738 (charged negative); ASP776 (charged negative); F, Cl halogen; (R)-THF ether
**12a**	-6.598	PHE699, ALA698, GLY697, GLY695, LEU694, VAL702, PHE771, PRO770, MET769, GLY772, CYS773, LEU768	ASN818, THR766, GLN767	ARG817 (H-bond backbone); MET769 (H-bond sidechain x2)	LYS721, ARG817, LYS704 (charged positive); ASP831, THR830 (polar); no halogen
**12b**	-5.228	PRO770, MET769, LEU768, GLN767, THR766, GLY772, CYS773, LEU694, MET742, VAL702, PHE699, ALA698, LEU834, ALA731	ASN818, THR830	LYS721 (Pi-cation, red lines); ASP831 (backbone)	LYS721, ARG817 (charged positive); GLU738, GLU734 (charged negative); ILE735
**12c**	-5.507	LEU694, LEU768, MET769, PHE771, GLY772, CYS773, VAL821, LEU820,	ASN818, GLN767, THR766	LYS721 (Pi-cation, red line); ASP831–THR830 (backbone)	LYS721, ARG817 (charged positive); GLU734, ASP831 (charged negative); no halogen

		MET742, PHE699, ILE735, ALA731, ALA719			
**Methotrexate**	-5.956	GLY695, LEU694, PHE771, PRO770, MET769, LEU768, CYS773, GLY772, PHE699, VAL702, LEU820	ASN818, THR830	ASP831 (H-bond sidechain); ASP776 (H-bond backbone); LYS692 (salt bridge)	LYS721, ARG817, LYS704, LYS692 (charged positive); ASP831, ASP776 (charged negative)

Fig S47: 2D molecular docking Diagram against 1M17 Protein target

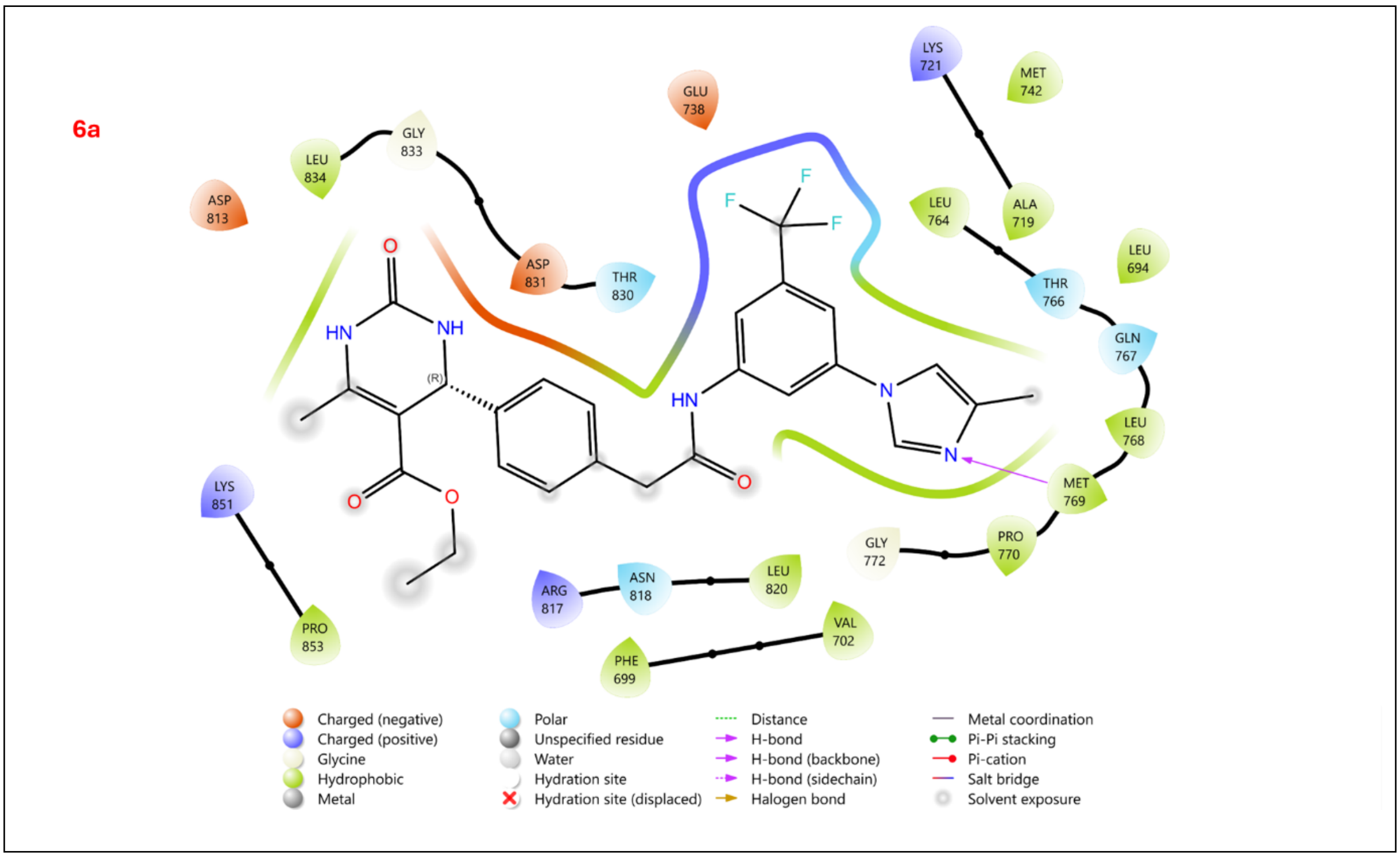

6b

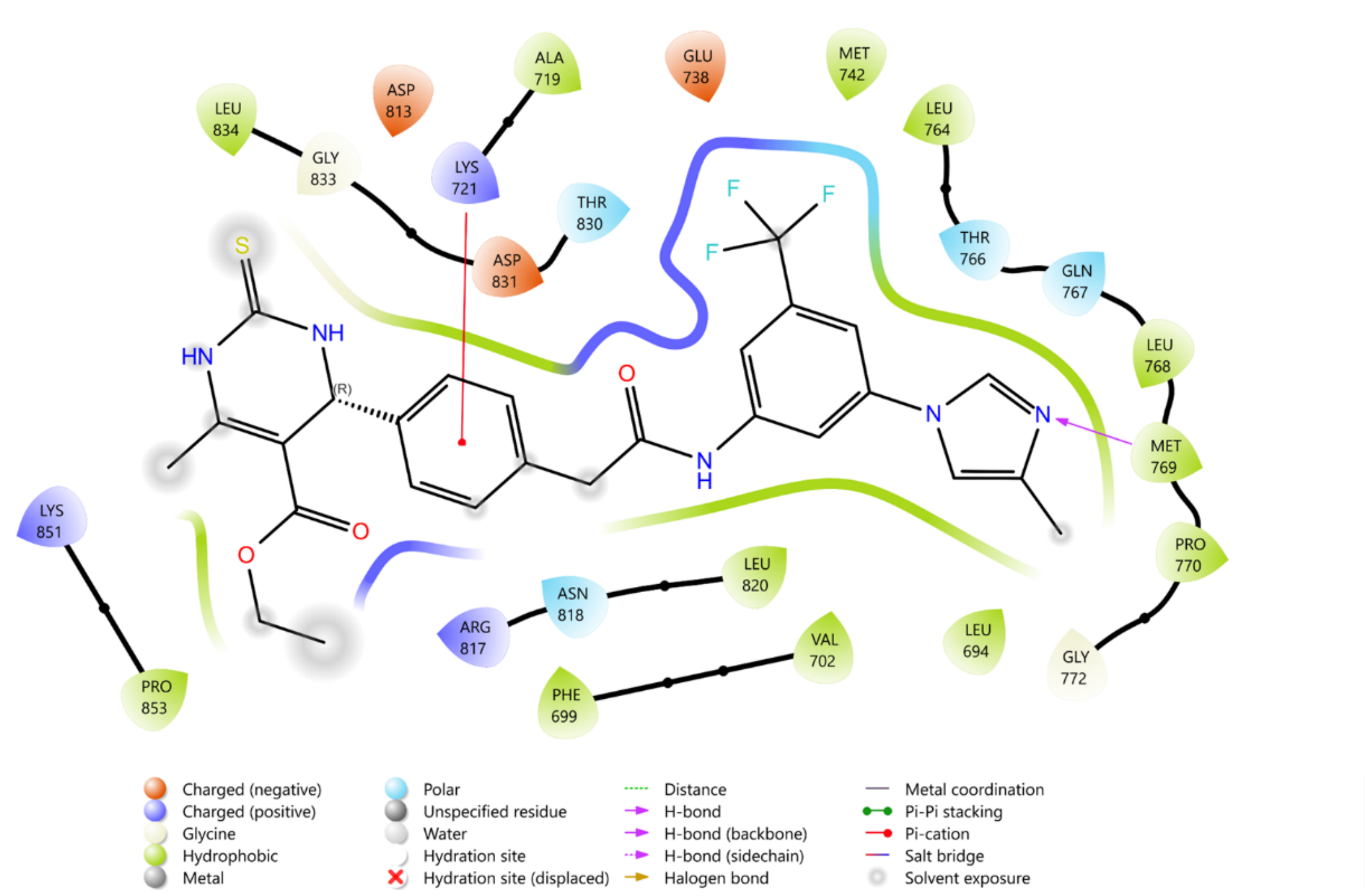
LEU 834
GLY 833
ASP 813
LYS 721
ALA 719
GLU 738
MET 742
LEU 764
THR 830
ASP 831
THR 766
GLN 767
LEU 768
MET 769
PRO 770
GLY 772
LEU 694
VAL 702
PHE 699
LEU 820
ASN 818
ARG 817
LYS 851
PRO 853
S
HN
NH
(R)
O
N
H
F
Charged (negative)
Charged (positive)
Glycine
Hydrophobic
Metal
Polar
Unspecified residue
Water
Hydration site
Hydration site (displaced)
Distance
H-bond
H-bond (backbone)
H-bond (sidechain)
Halogen bond
Metal coordination
Pi-Pi stacking
Pi-cation
Salt bridge
Solvent exposure

6c

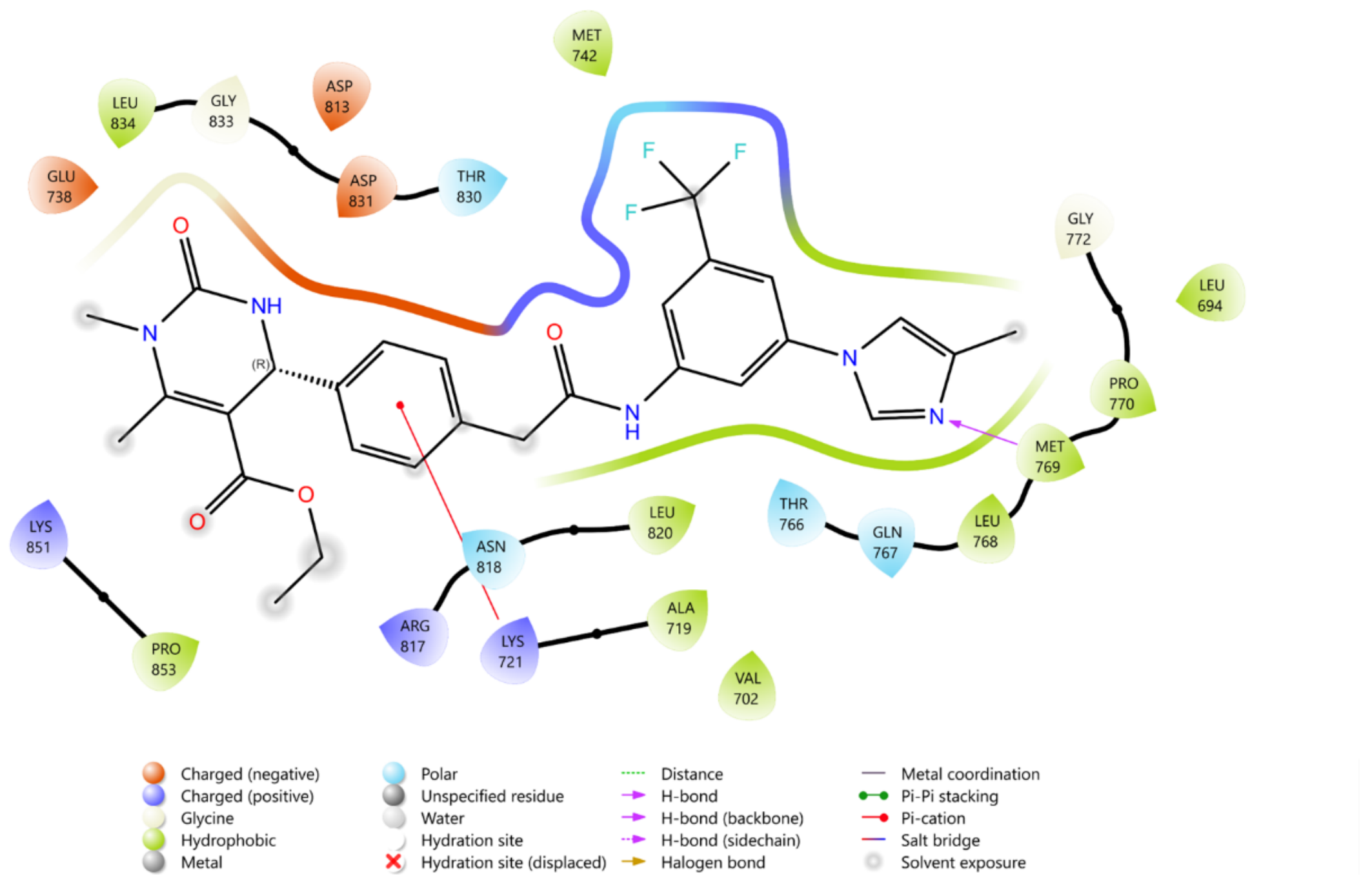
MET 742
LEU 834
GLY 833
ASP 813
GLU 738
ASP 831
THR 830
GLY 772
LEU 694
PRO 770
MET 769
LEU 768
GLN 767
THR 766
LEU 820
ASN 818
ARG 817
LYS 721
ALA 719
VAL 702
LYS 851
PRO 853
O
N
NH
(R)
H
F
Charged (negative)
Charged (positive)
Glycine
Hydrophobic
Metal
Polar
Unspecified residue
Water
Hydration site
Hydration site (displaced)
Distance
H-bond
H-bond (backbone)
H-bond (sidechain)
Halogen bond
Metal coordination
Pi-Pi stacking
Pi-cation
Salt bridge
Solvent exposure

8a

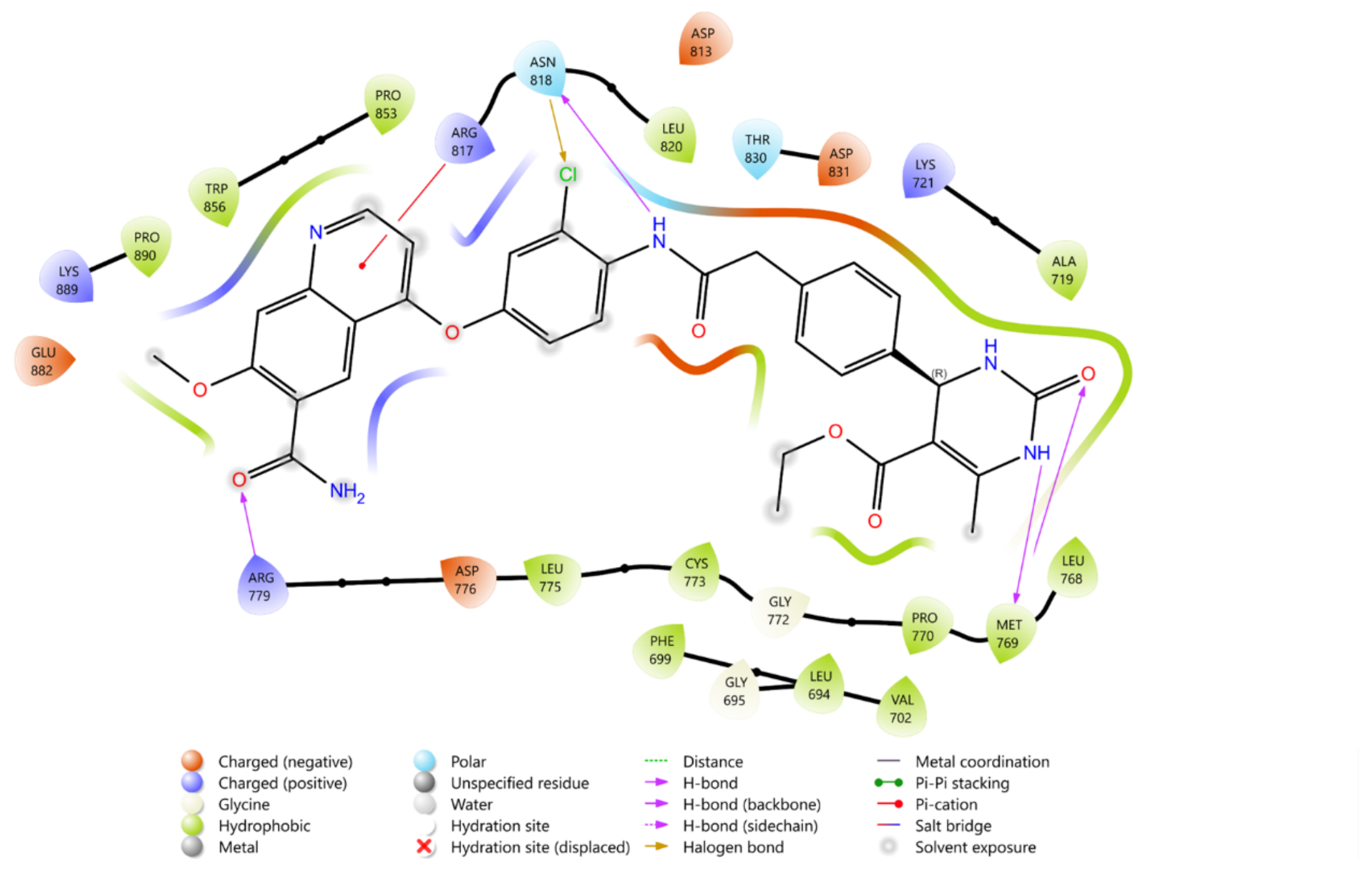
ASN 818
ASP 813
PRO 853
ARG 817
LEU 820
THR 830
ASP 831
LYS 721
TRP 856
PRO 890
LYS 889
ALA 719
GLU 882
ARG 779
ASP 776
LEU 775
CYS 773
GLY 772
PRO 770
MET 769
LEU 768
PHE 699
GLY 695
LEU 694
VAL 702
Charged (negative)
Charged (positive)
Glycine
Hydrophobic
Metal
Polar
Unspecified residue
Water
Hydration site
Hydration site (displaced)
Distance
H-bond
H-bond (backbone)
H-bond (sidechain)
Halogen bond
Metal coordination
Pi-Pi stacking
Pi-cation
Salt bridge
Solvent exposure

8b

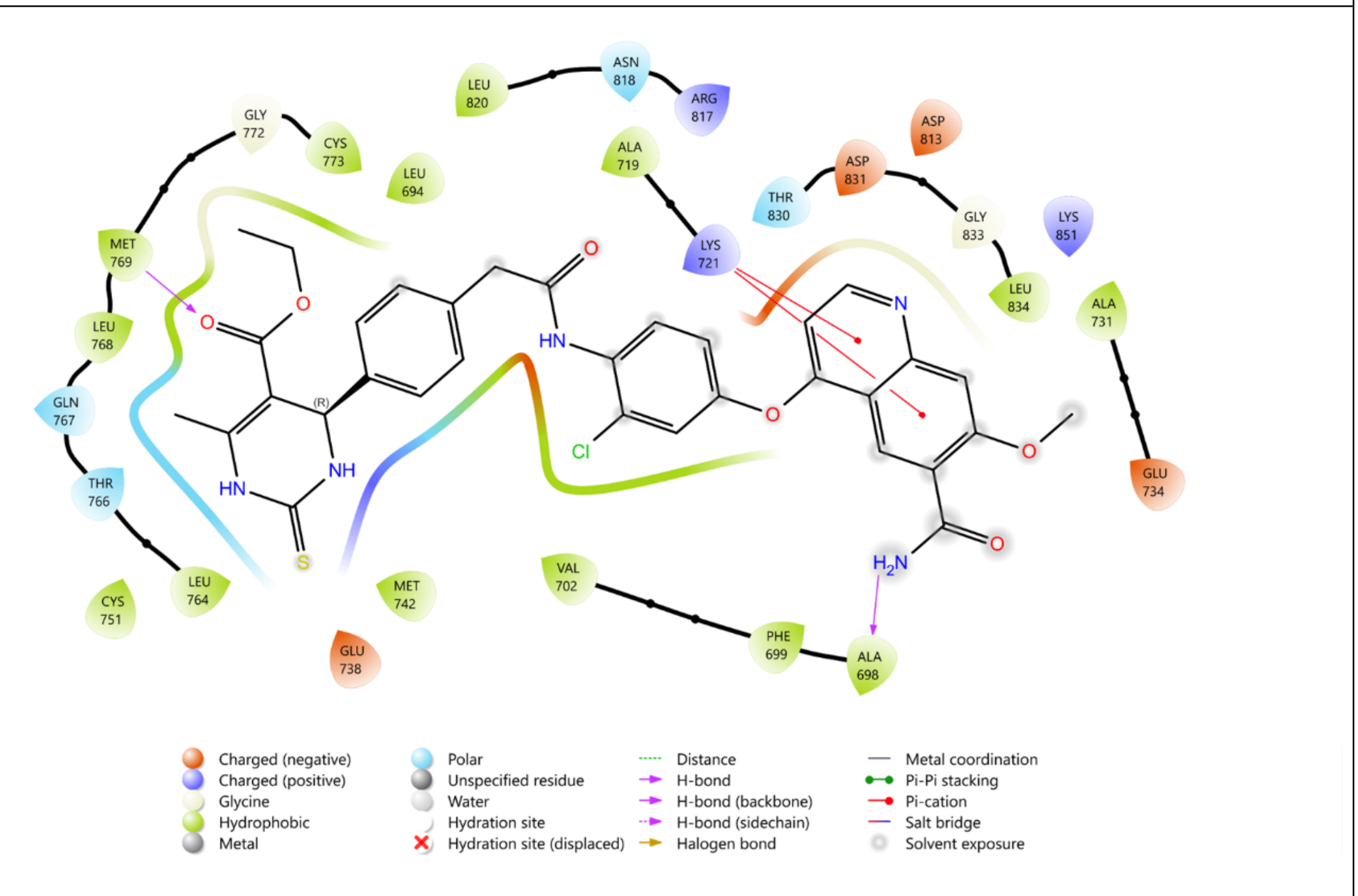
LEU 820
ASN 818
ARG 817
GLY 772
CYS 773
LEU 694
ALA 719
ASP 813
ASP 831
THR 830
GLY 833
LYS 851
MET 769
LYS 721
LEU 834
ALA 731
LEU 768
GLN 767
THR 766
GLU 734
LEU 764
CYS 751
MET 742
GLU 738
VAL 702
PHE 699
ALA 698
Charged (negative)
Charged (positive)
Glycine
Hydrophobic
Metal
Polar
Unspecified residue
Water
Hydration site
Hydration site (displaced)
Distance
H-bond
H-bond (backbone)
H-bond (sidechain)
Halogen bond
Metal coordination
Pi-Pi stacking
Pi-cation
Salt bridge
Solvent exposure

8c

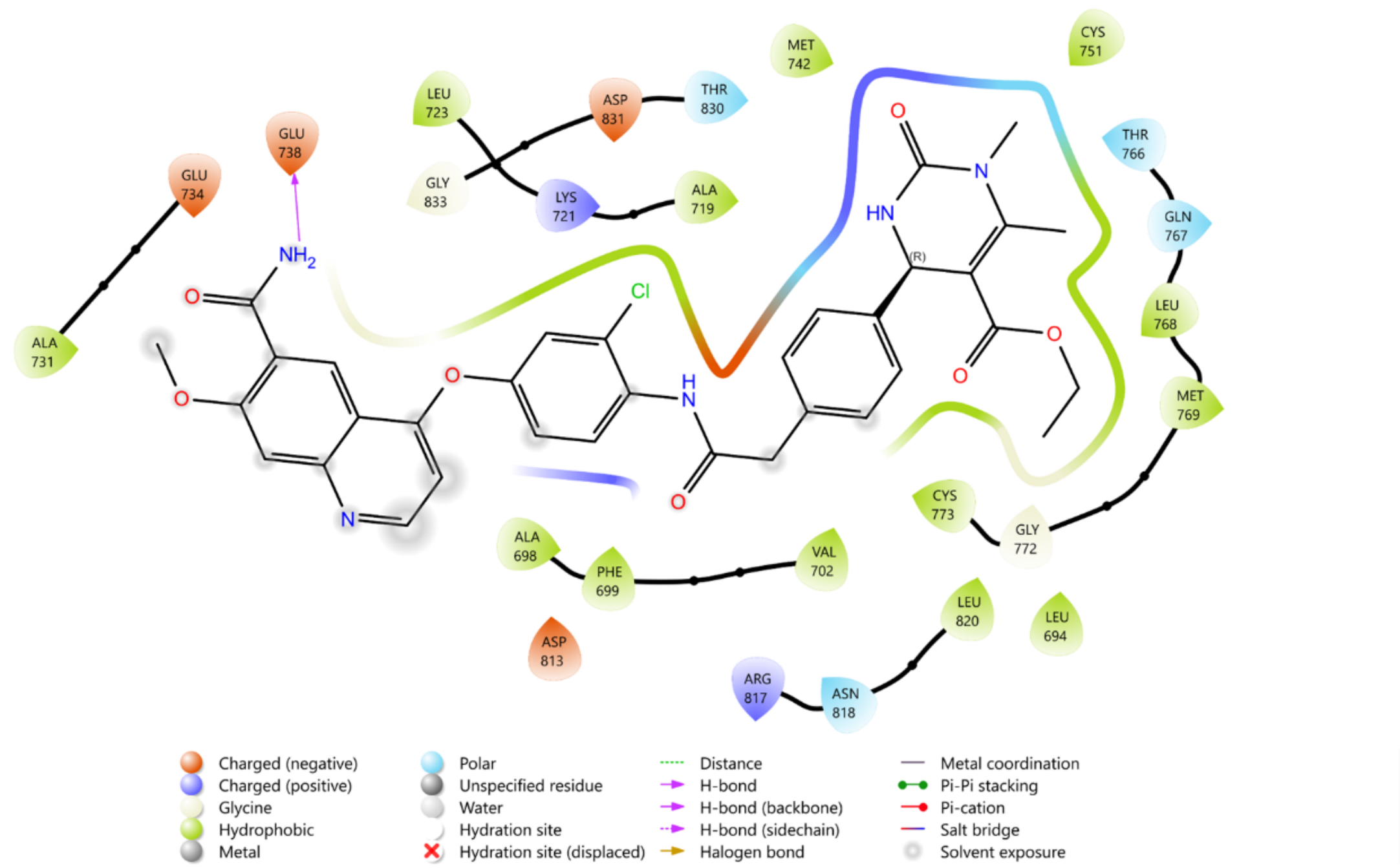
GLU 734
ALA 731
GLU 738
NH2
LEU 723
GLY 833
ASP 831
THR 830
LYS 721
ALA 719
MET 742
CYS 751
THR 766
GLN 767
LEU 768
MET 769
CYS 773
GLY 772
ALA 698
PHE 699
VAL 702
ASP 813
LEU 820
LEU 694
ARG 817
ASN 818
Cl
Charged (negative)
Charged (positive)
Glycine
Hydrophobic
Metal
Polar
Unspecified residue
Water
Hydration site
Hydration site (displaced)
Distance
H-bond
H-bond (backbone)
H-bond (sidechain)
Halogen bond
Metal coordination
Pi-Pi stacking
Pi-cation
Salt bridge
Solvent exposure

10a

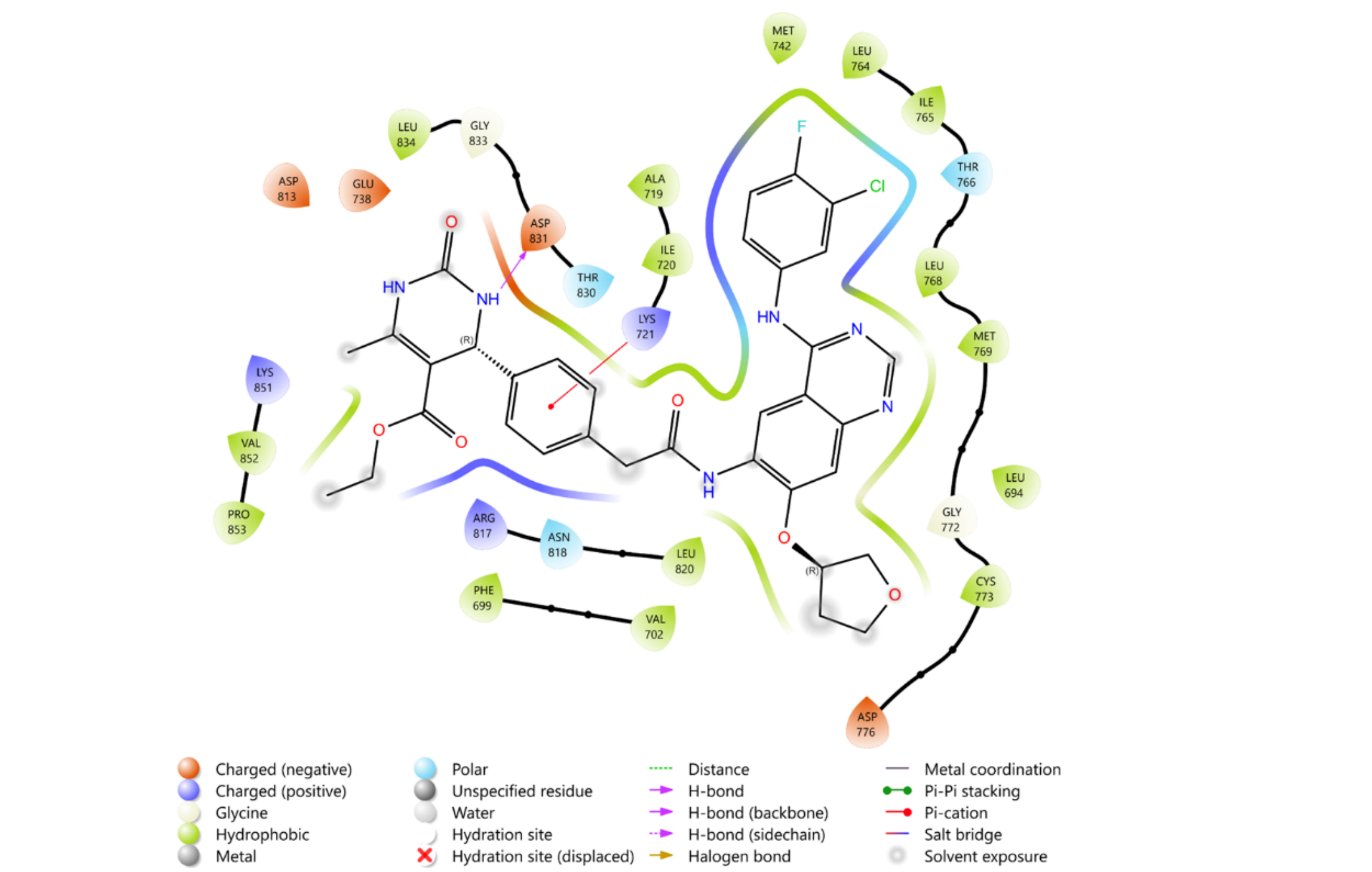
MET 742
LEU 764
ILE 765
THR 766
LEU 768
MET 769
LEU 694
GLY 772
CYS 773
ASP 776
LEU 834
GLY 833
ASP 813
GLU 738
ASP 831
THR 830
ALA 719
ILE 720
LYS 721
LYS 851
VAL 852
PRO 853
ARG 817
ASN 818
LEU 820
PHE 699
VAL 702
F
Cl
Charged (negative)
Charged (positive)
Glycine
Hydrophobic
Metal
Polar
Unspecified residue
Water
Hydration site
Hydration site (displaced)
Distance
H-bond
H-bond (backbone)
H-bond (sidechain)
Halogen bond
Metal coordination
Pi-Pi stacking
Pi-cation
Salt bridge
Solvent exposure

10b

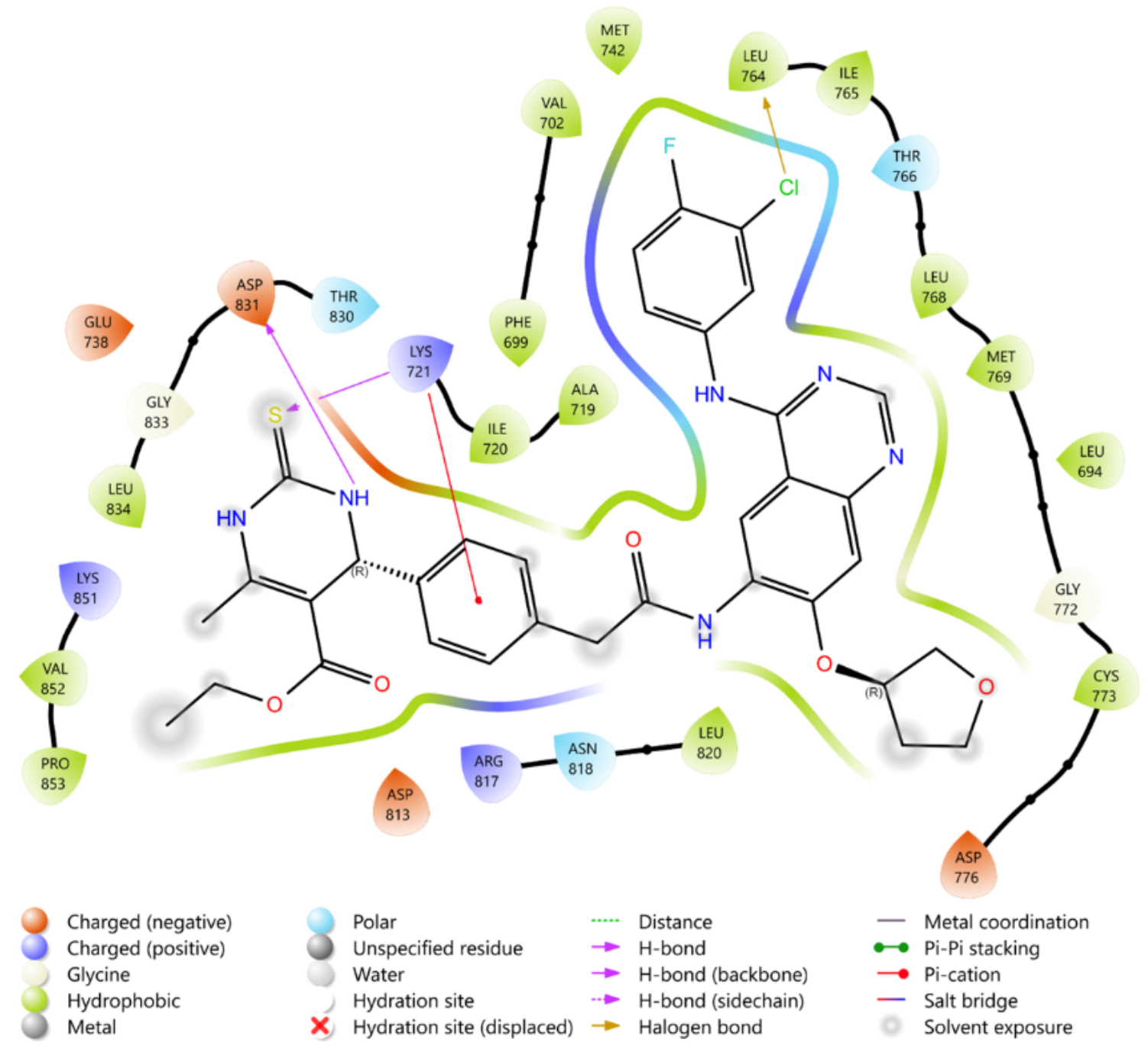

MET 742
VAL 702
LEU 764
ILE 765
THR 766
LEU 768
MET 769
LEU 694
GLY 772
CYS 773
ASP 776
LEU 820
ASN 818
ARG 817
ASP 813
PRO 853
VAL 852
LYS 851
LEU 834
GLY 833
GLU 738
ASP 831
THR 830
LYS 721
PHE 699
ALA 719
ILE 720
F
Cl
HN
N
N
S
NH
HN
O
N
H
O
O
O
O
(R)
(R)
Charged (negative)
Charged (positive)
Glycine
Hydrophobic
Metal
Polar
Unspecified residue
Water
Hydration site
Hydration site (displaced)
Distance
H-bond
H-bond (backbone)
H-bond (sidechain)
Halogen bond
Metal coordination
Pi-Pi stacking
Pi-cation
Salt bridge
Solvent exposure

10c

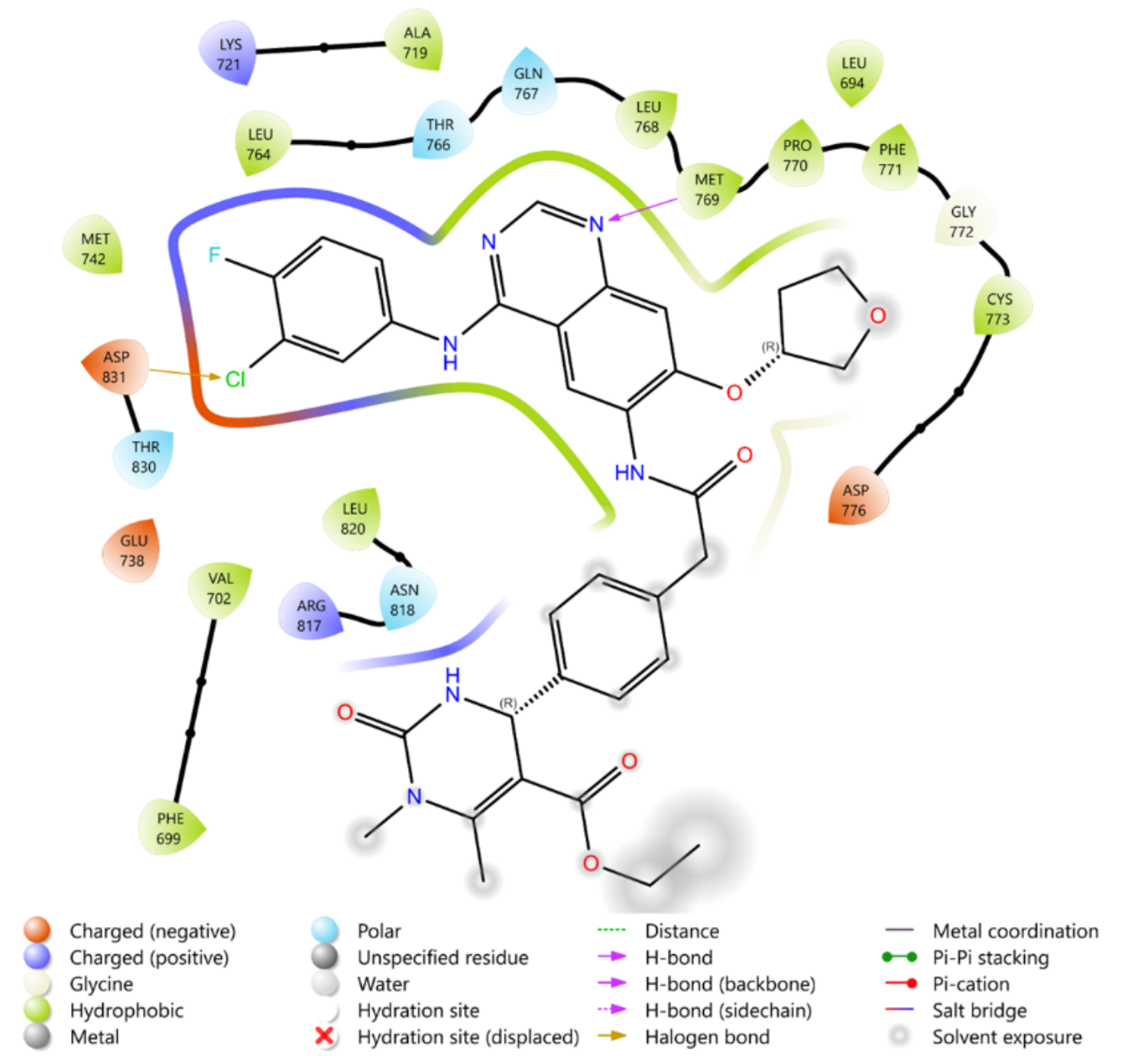

LYS 721
ALA 719
GLN 767
LEU 768
LEU 694
LEU 764
THR 766
PRO 770
PHE 771
MET 769
GLY 772
CYS 773
MET 742
ASP 831
THR 830
GLU 738
VAL 702
PHE 699
LEU 820
ASN 818
ARG 817
ASP 776
F
Cl
N
N
N
H
O
O
HN
O
H
N
O
N
O
O
(R)
(R)
Charged (negative)
Charged (positive)
Glycine
Hydrophobic
Metal
Polar
Unspecified residue
Water
Hydration site
Hydration site (displaced)
Distance
H-bond
H-bond (backbone)
H-bond (sidechain)
Halogen bond
Metal coordination
Pi-Pi stacking
Pi-cation
Salt bridge
Solvent exposure

12a

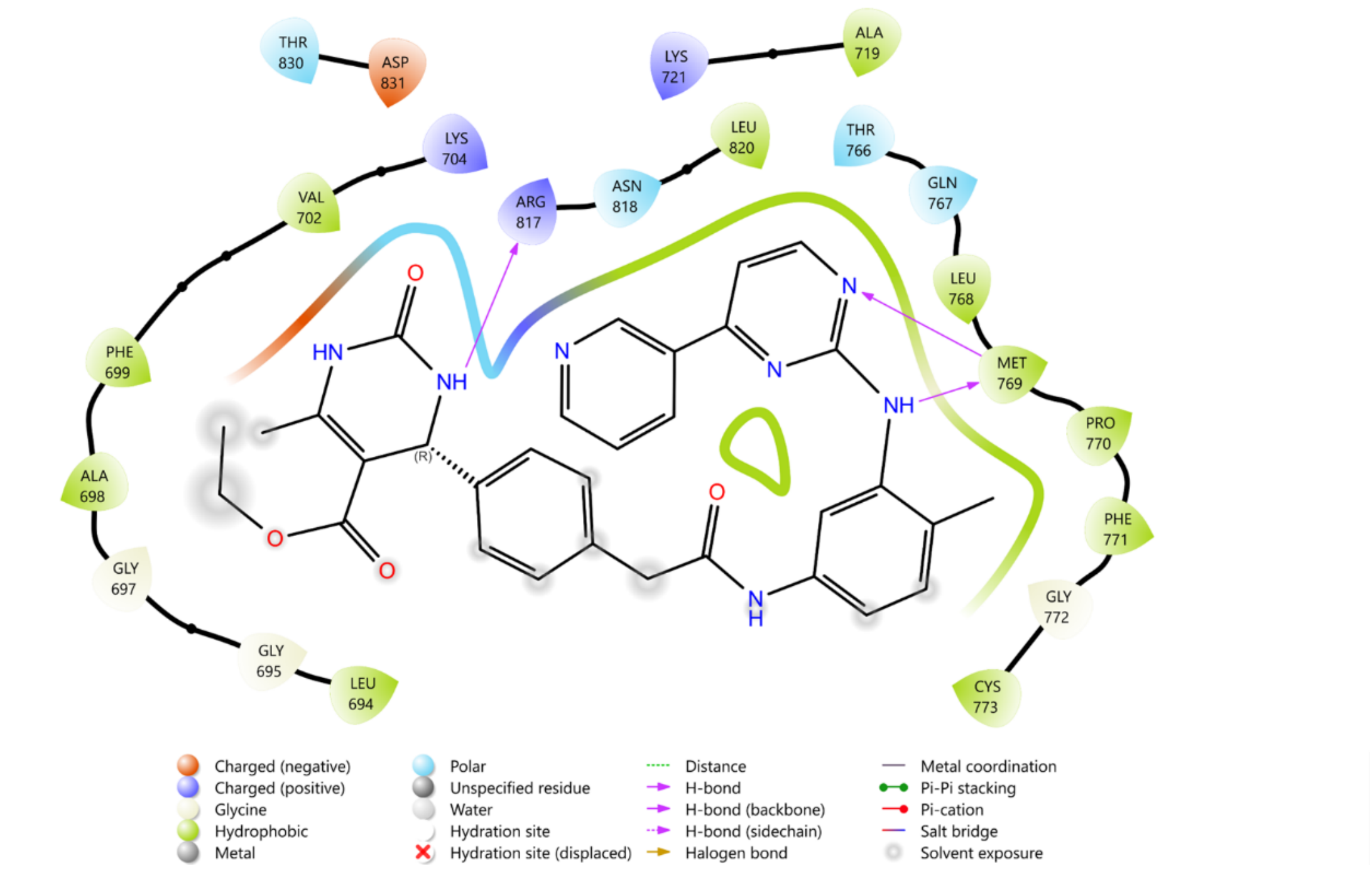
THR 830
ASP 831
LYS 721
ALA 719
LYS 704
VAL 702
ARG 817
ASN 818
LEU 820
THR 766
GLN 767
LEU 768
MET 769
PRO 770
PHE 771
GLY 772
CYS 773
PHE 699
ALA 698
GLY 697
GLY 695
LEU 694
Charged (negative)
Charged (positive)
Glycine
Hydrophobic
Metal
Polar
Unspecified residue
Water
Hydration site
Hydration site (displaced)
Distance
H-bond
H-bond (backbone)
H-bond (sidechain)
Halogen bond
Metal coordination
Pi-Pi stacking
Pi-cation
Salt bridge
Solvent exposure

12b

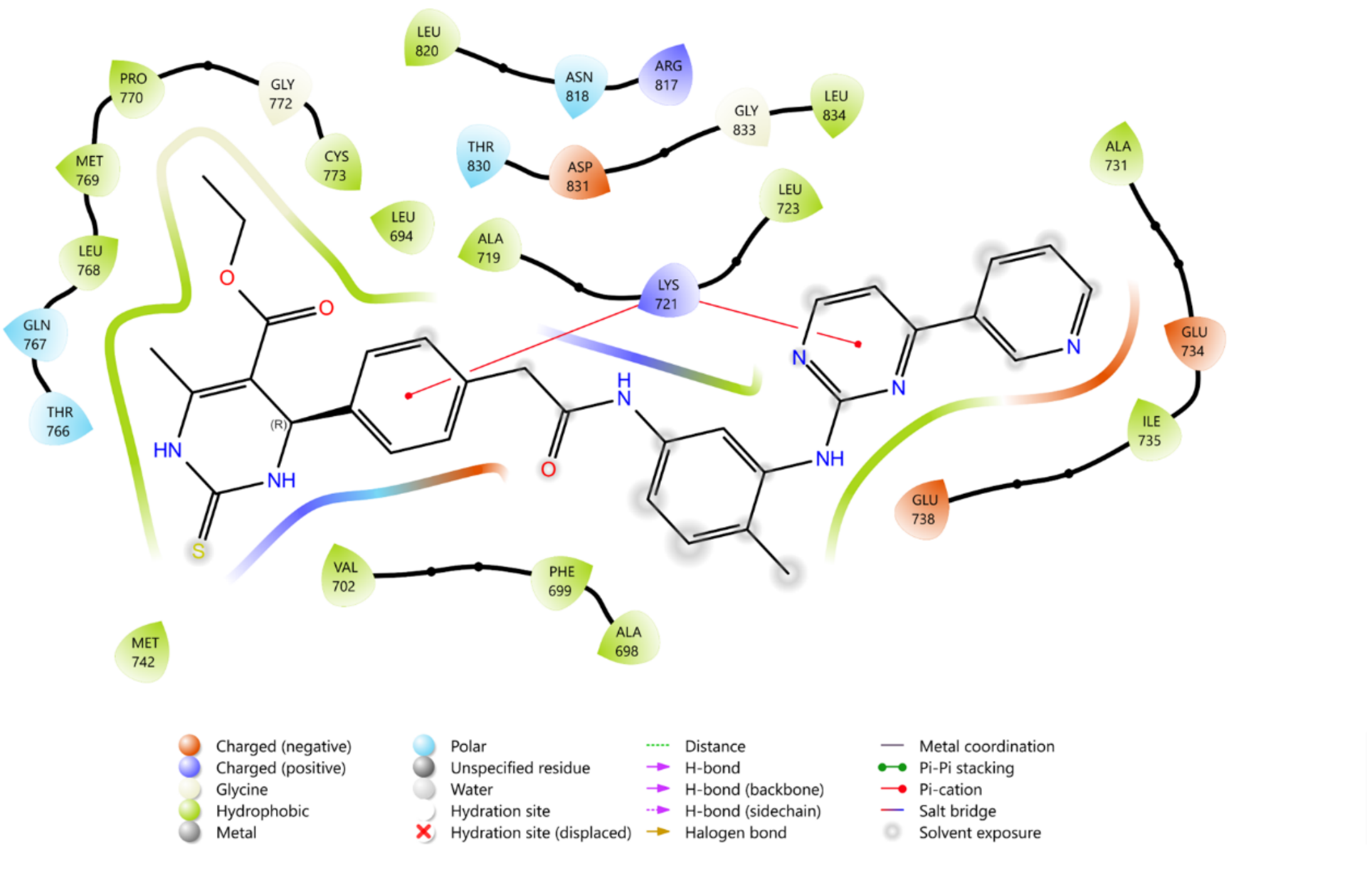
LEU 820
ASN 818
ARG 817
PRO 770
GLY 772
GLY 833
LEU 834
MET 769
CYS 773
THR 830
ASP 831
ALA 731
LEU 768
LEU 694
ALA 719
LEU 723
LYS 721
GLN 767
GLU 734
THR 766
ILE 735
GLU 738
VAL 702
PHE 699
ALA 698
MET 742
Charged (negative)
Charged (positive)
Glycine
Hydrophobic
Metal
Polar
Unspecified residue
Water
Hydration site
Hydration site (displaced)
Distance
H-bond
H-bond (backbone)
H-bond (sidechain)
Halogen bond
Metal coordination
Pi-Pi stacking
Pi-cation
Salt bridge
Solvent exposure

12c

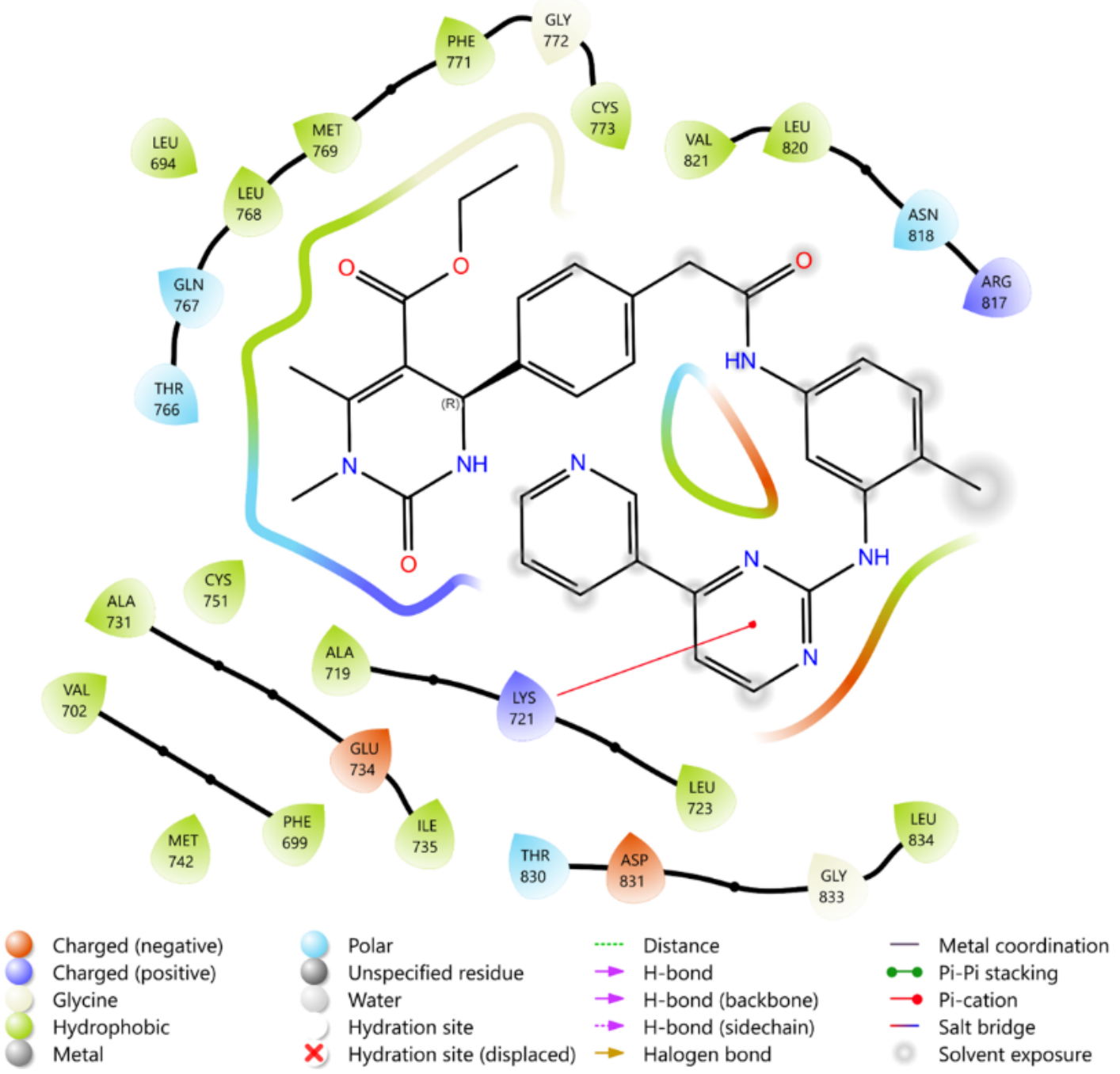

Methotrexate

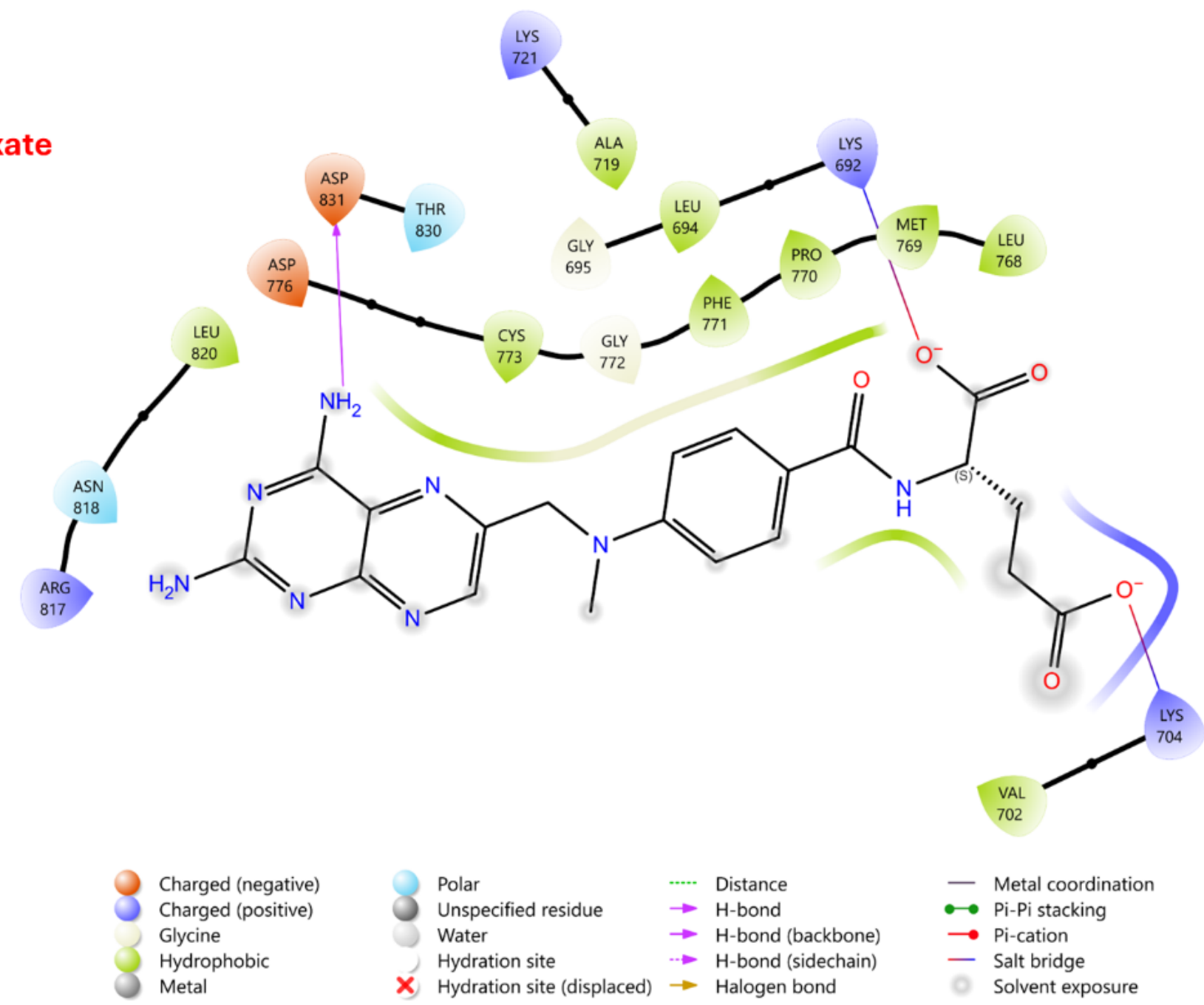

Mulliken charge distributions:

Table S3. Mulliken Atomic Charges of Selected Atoms in the Optimized Structure (Compound 10b)

Sr. No	Atom	Chagre (Q/e)	Sr. No	Atom	Chagre (Q/e)
1	N	-0.6552	41	F	-0.25215
2	C	0.22613	42	N	-0.84797
3	C	-0.1492	43	O	-0.58008
4	N	-0.76302	44	C	-0.00586
5	C	-0.08988	45	C	-0.11951
6	C	0.372138	46	C	-0.44992
7	C	0.057369	47	O	-0.4675
8	C	-0.20686	48	C	-0.1406
9	C	-0.20782	49	H	0.351053
10	C	-0.19525	50	H	0.258318
11	C	-0.18958	51	H	0.354932
12	C	0.033873	52	H	0.218331
13	C	-0.51926	53	H	0.206243
14	C	0.707009	54	H	0.209365
15	O	-0.49427	55	H	0.175794
16	S	-0.04916	56	H	0.251357
17	C	0.719942	57	H	0.191019
18	C	-0.59101	58	H	0.252163

19	O	-0.52302	59	H	0.18344
20	O	-0.52966	60	H	0.243045
21	C	-0.16436	61	H	0.210604
22	C	-0.59087	62	H	0.223175
23	C	0.238856	63	H	0.193273
24	C	-0.00202	64	H	0.217816
25	C	-0.18027	65	H	0.201983
26	C	-0.2279	66	H	0.211644
27	C	0.30755	67	H	0.26437
28	C	0.33572	68	H	0.21699
29	N	-0.59256	69	H	0.332971
30	C	0.213973	70	H	0.23805
31	C	0.605991	71	H	0.212417
32	N	-0.59843	72	H	0.230946
33	N	-0.83721	73	H	0.343321
34	C	0.255687	74	H	0.227722
35	C	-0.12945	75	H	0.220967
36	C	-0.16723	76	H	0.190703
37	C	-0.32323	77	H	0.222506
38	C	-0.20007	78	H	0.227505
39	C	0.33662	79	H	0.203194
40	Cl	0.132811	80	H	0.211457
